# Reoptimization of the Organocatalyzed Double Aldol Domino Process to a Key Enal Intermediate and Its Application to the Total Synthesis of Δ^12^‐Prostaglandin J_3_

**DOI:** 10.1002/chem.201802498

**Published:** 2018-06-06

**Authors:** Andrejs Pelšs, Narasimhulu Gandhamsetty, James R. Smith, Damien Mailhol, Mattia Silvi, Andrew J. A. Watson, Isabel Perez‐Powell, Sébastien Prévost, Nina Schützenmeister, Peter R. Moore, Varinder K. Aggarwal

**Affiliations:** ^1^ School of Chemistry University of Bristol Cantock's Close Bristol BS8 1TS U.K.; ^2^ Pharmaceutical Technology and Development AstraZeneca Silk Road Business Park, Charter Way Macclesfield SK10 2NA U.K.

**Keywords:** aldol reaction, organocatalysis, prostaglandins, total synthesis, Δ^12^-PGJ_3_

## Abstract

Re‐investigation of the l‐proline catalyzed double aldol cascade dimerization of succinaldehyde for the synthesis of a key bicyclic enal intermediate, pertinent in the field of stereoselective prostaglandin synthesis, is reported. The yield of this process has been more than doubled, from 14 % to a 29 % isolated yield on a multi‐gram scale (32 % NMR yield), through conducting a detailed study of the reaction solvent, temperature, and concentration, as well as a catalyst screen. The synthetic utility of this enal intermediate has been further demonstrated through the total synthesis of Δ^12^‐prostaglandin J_3_, a compound with known anti‐leukemic properties.

Interest in the chemistry and biology of prostanoids continues unabated, with as many publications (ca. 600 per year) in the last decade as during their heyday of the 1970s.^1^ Prostaglandins are an important class of potent lipid mediators that are involved in the regulation of many biological processes^2^ such as inflammation,^3^ pain response,^4^ and fever.^5^ Consequently, this class of compounds has found wide‐spread use as pharmaceuticals^6^ for the treatment of several diseases including pulmonary arterial hypertension^7^ and glaucoma.^8^ We recently described a dramatically short route to the prostaglandins, completing the total synthesis of PGF_2α_ in just seven steps (Scheme 1),^9^ and its subsequent application to the concise synthesis of the related pharmaceutical analogues latanaprost and bimatoprost,^10^ and alfaprostol.^11^ The key step in our synthesis is the double aldol dimerization of succinaldehyde with proline and dibenzylammonium trifluoroacetate (DBA) as catalysts to give the bicyclic enal intermediate (**1**) in high enantioselectivity (Scheme 1).^12^ Whilst this single step converts a simple starting material into a complex intermediate^13^ fully primed to enable rapid attachment of the two side chains required, its Achilles’ heel is its low yield (caused by the extensive oligomerization of succinaldehyde under the reaction conditions).

**Scheme 1:**
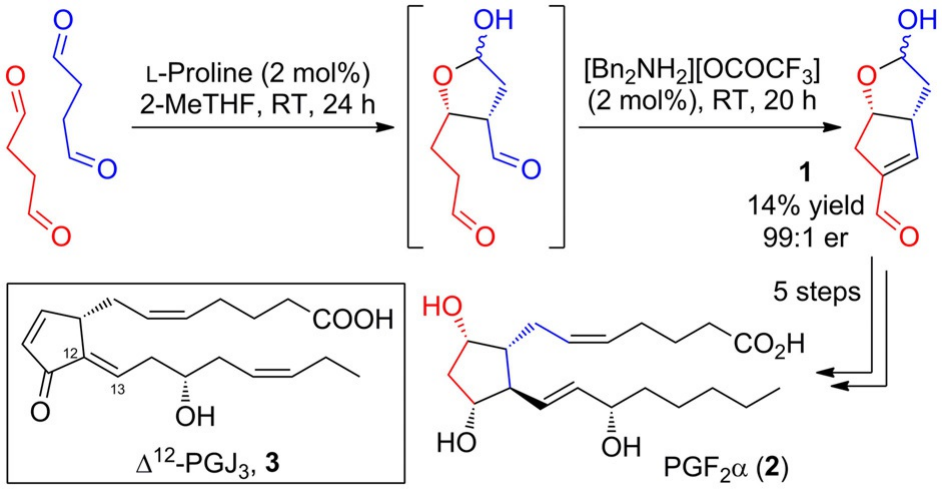
Previously reported l‐proline catalyzed double aldol domino reaction for the synthesis of a key bicyclic enal intermediate **1** and its application to the total synthesis of PGF_2α_ (**2**).

In this paper, we describe a full re‐investigation of this key step, which has culminated in an increase in yield from 14 %9a to a 29 % isolated yield on a multi‐gram scale, thereby enabling the enal **1** to be used not just in further, more efficient prostanoid syntheses, but also as a highly functionalized and useful building block more generally. Furthermore, we demonstrate its application to the concise synthesis of Δ^12^‐PGJ_3_, a prostanoid of considerable contemporary interest due to its high activity against cancer stem cells, a class of cells that are notoriously resistant toward conventional chemical therapies.^14^

The aldol dimerization of succinaldehyde is complex: l‐proline (2 %) is added to a 2 m solution of succinaldehyde in Me‐THF and after 24 h the mixture is diluted to 1 m and dibenzylammonium trifluoroacetate (**5**, 2 %) is added. The two catalysts perform different roles: l‐proline catalyzes the first aldol but does not catalyze the second aldol and **5** catalyzes the second aldol condensation and does not (and must not) catalyze the first aldol otherwise the reaction would occur with low enantioselectivity. The catalysts must be added sequentially because **5** inhibits the first aldol reaction with proline and the time of addition is important since reaction of succinaldehyde with l‐proline leads to oligomers over time. Under these conditions, a 14 % yield was achieved (Table 1, entry 1), and so we embarked on a further optimization program re‐investigating all the parameters. A small improvement in yield (16 %) was achieved by changing the solvent to acetonitrile (Table 1, entry 2). The yield was further increased to 19 % (NMR) by reducing the initial concentration to 1 m (entry 3), but under these reaction conditions, the enal was isolated in only 9 % yield. This disparity between the NMR and isolated yields was a consequence of the formation of a hemiaminal ether by‐product **4**, which formed upon addition of catalyst **5** to the lactol moiety of **1** during the isolation process. An extensive screening of the second catalyst was undertaken, and we found that the formation of the hemiaminal ether by‐product could be avoided using thiomorpholinium trifluoroacetate **6**, resulting in 20 % yield (both NMR and isolated) of the enal **1** (entry 4). Increasing the temperature of the second step led to an improved 23 % yield in just 2 h (entry 5). An extensive solvent study (see the Supporting Information for further examples) revealed that whilst ethyl acetate performed similarly to acetonitrile (21 % vs. 23 %, entries 5 and 6, respectively), it led to significantly less oligomerization of succinaldehyde. Reducing the concentration further in both the first and second phases of the reaction resulted in further improvements (entries 7 and 8) culminating in a 33 % NMR yield and 31 % isolated yield (entry 8). On scale, isolation was initially problematic. Evaporation followed by chromatography was ineffective, giving enal **1** contaminated by both oligomers and succinaldehyde. We were wary of carrying out an aqueous work‐up because we had previously found that the enal is partially water soluble and required multiple extractions even from brine solutions, resulting in excessively large volumes of solvent. However, a recent report highlighting the beneficial effects of using Na_2_SO_4_ to salt‐out water soluble compounds prompted us to reinvestigate aqueous work‐ups.^15^ Using this strategy, just two extractions enabled full recovery of enal **1**, which was purified by column chromatography; it was essential to pre‐treat the silica gel with water (50 wt %) to ensure efficient removal of any remaining succinaldehyde and oligomeric material during chromatography. Using our optimized conditions and improved work‐up and purification, 50 g of succinaldehyde was transformed into 12.8 g of enal **1** (29 % isolated yield) as an inconsequential 4:1 mixture of diastereomers, and with the same enantiomeric ratio (99:1 er) as previously reported.

**Table 1:** Re‐optimization of the synthesis of enal **1**.^[a]^

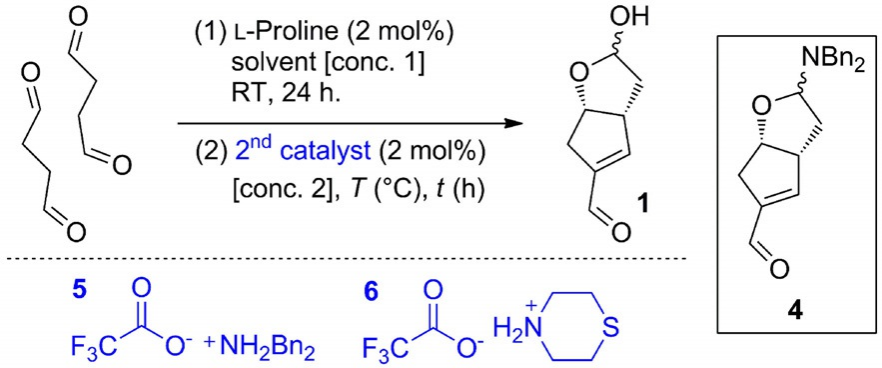							
Entry	Solvent	2nd Cat.	*T* [°C]	*t* [h]	Conc. **1** [m]	Conc. **2** [m]	Yield [%]^[b]^
1	THF	**5**	RT	14	2.0	1.0	14
2	MeCN	**5**	RT	20	2.0	1.0	16
3	MeCN	**5** ^[c]^	RT	24	1.0	1.0	19 (9)
4	MeCN	**6** ^[c]^	RT	24	1.0	1.0	20 (20)
5	MeCN	**6**	65	2	2.0	2.0	23
6	EtOAc	**6**	65	2	2.0	2.0	21
7	EtOAc	**6**	65	2	1.0	0.5	28
8^[d]^	EtOAc	**6**	65	2	0.75	0.2	33 (31)
9^[d,e]^	EtOAc	**6**	65	2	0.75	0.35	32 (29)

[a] Reaction conditions (unless otherwise stated): Succinaldehyde (5.81 mmol), l‐proline (2 mol %), solvent (*X* m), RT; then: 2nd catalyst (2 mol %). [b] Yield determined by ^1^H NMR spectroscopy using 1,3,5‐trimethoxybenzene as an internal standard (isolated yields following chromatographic purification are shown in parentheses). [c] 5 mol % of the 2nd catalyst was used. [d] 40 h reaction time for 1st step. [e] 50 g succinaldehyde (581 mmol) was used.

In order to further exemplify the synthetic utility of the enal **1**, we chose Δ^12^‐prostaglandin J_3_ (Δ^12^‐PGJ_3_, **3**, Scheme 1) as a target because of its considerable biological potency towards stem cell cancer.^14^ Nicolaou and co‐workers recently reported several strategies for the total syntheses of Δ^12^‐PGJ_3_.^16^ These strategies were subsequently applied to the development of a series of even more potent analogues as potential clinical candidates for stem cell cancer treatment, making this an even more sought‐after target.^17^ We have previously used **1** to form the C12−C13 bond (prostaglandin numbering) in the syntheses of a variety of prostanoids through conjugate addition of a nucleophile to the electrophilic enal moiety (Scheme 2 A). We reasoned that, by transforming enal **1** into enamide **7**, it would change the reactivity of the C12‐position from electrophilic to nucleophilic, further broadening the utility of our key enal (Scheme 2 A). In order to construct Δ^12^‐PGJ_3,_ disconnection of the C12−C13 bond through an aldol/dehydration reaction sequence would require β‐boryl aldehyde **8** and enone **9** (Scheme 2 B). This type of aldol/dehydration strategy was originally reported by Kobayashi^18^ and has subsequently been applied to the syntheses of Δ^12^‐PGJ_3_ **3** 16a,16b, 17 and other closely related prostaglandins.^19^ Boryl aldehyde **8** was selected as a masked hydroxy aldehyde equivalent because it can be easily prepared by catalytic enantioselective conjugate borylation,^20^ and subsequently unmasked later in the synthesis by stereospecific oxidation of the boronic ester.

**Scheme 2:**
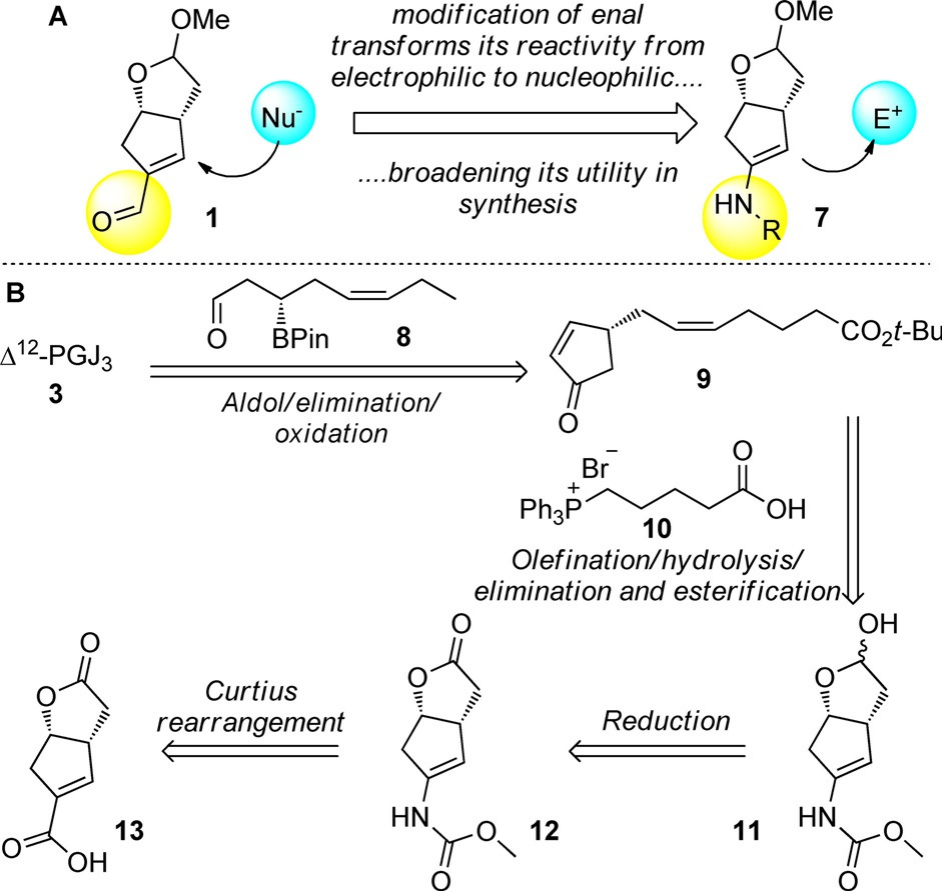
A) Changing reactivity mode of our bicyclic enal intermediate from electrophilic to nucleophilic. B) Retrosynthetic analysis of Δ^12^‐PGJ_3_ **3**.

Furthermore, it avoids potential elimination of a protected δ‐alkoxy enone to the dieneone. Enone **9** could be obtained by olefination of hemiacetal **11**, followed by hydrolysis of the carbamate group and elimination. The carbamate itself could be derived from carboxylic acid **13**.

The synthesis of Δ^12^‐PGJ_3_ **3** began through a double oxidation of enal **1** to the lactone‐acid **13** using standard Pinnick oxidation conditions in 74 % yield (Scheme 3). Carboxylic acid **13** was initially converted into acyl azide **14** in 80 % yield. Heating **14** in toluene effected a Curtius rearrangement to afford an intermediate isocyanate, which was trapped with benzyl alcohol to give carbamate **12** in 90 % yield. While it was possible to obtain **12** directly by adding benzyl alcohol to the cooled toluene solution, this led to sluggish nucleophilic capture reactions. It was crucial to remove toluene before alcohol addition to ensure a high yield of **12**. Ene‐carbamate **12** was then reduced to hemiacetal **11** with DIBAL‐H in 98 % yield. Initially, the Wittig reaction between hemiacetal **11** and phosphonium salt **10** was problematic due to poor conversion of **11** and the enamide product **15** was found to be difficult to isolate perhaps due to its limited stability. After considerable optimization, it was found that isolation of the enamide **15** could be avoided by first treating the hemiacetal **11** with excess phosphonium salt **10** and KO*t*‐Amyl in THF. Direct addition of degassed water and *para*‐toluenesulfonic acid to the reaction mixture effected the hydrolysis of the enamide group and dehydration to give enone **16** in 79 % yield. The enone acid **16** was subsequently converted into the *tert*‐butyl ester **9** in 83 % yield.

**Scheme 3:**
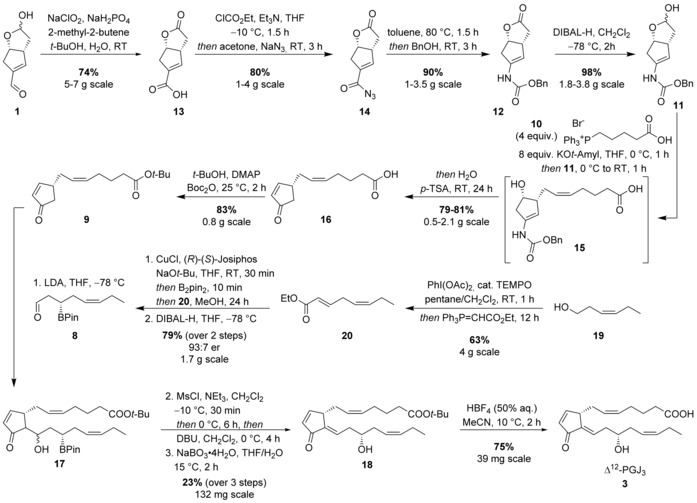
Synthetic route to Δ^12^‐PGJ_3_ (**3**) from enal **1** and leaf alcohol (**19**).

The lower side chain was prepared in a three‐step process from leaf alcohol **19** (Scheme 3). Following a known literature procedure,^21^ **19** was converted into α,β‐unsaturated ester **20** by initial oxidation to the corresponding alcohol, followed by a direct Wittig reaction in the same pot. Ester **20** was subjected to catalytic asymmetric conjugate addition with B_2_pin_2_,20a to provide a β‐boryl ester in high yield and enantiomeric ratio (85 %, 93:7 e.r.). The boronic ester was subsequently reduced to the required β‐boryl aldehyde **8** in 93 % yield upon treatment with DIBAL‐H.

To complete the synthesis of Δ^12^‐PGJ_3_ (**3**), we commenced the development of an aldol reaction between enantiomerically enriched fragments **8** and **9** (Scheme 3). Initially, 1.2 equivalents of LDA were added dropwise to a solution of **8** and **9** at −78 °C to give the expected aldol product **17** as a mixture of C13‐epimers in 23 % NMR yield (Scheme 3). Increasing the loading of LDA to 2.0 equivalents dramatically improved the reaction to provide **17** in a 75 % NMR yield. Due to the instability of β‐hydroxy boronic ester **17** towards column chromatography, the crude reaction mixture from the aldol reaction was treated with MsCl and NEt_3_ to give the corresponding mesylate, and subsequent elimination upon reaction with DBU produced exclusively the *E*‐configured elimination product. The resulting boronic ester was oxidized to secondary alcohol **18** using NaBO_3_⋅4 H_2_O in 23 % overall yield (over the three steps from **8** and **9**). Finally, treatment with HBF_4_ gave Δ^12^‐PGJ_3_ (**3**) in a 75 % yield. The total synthesis of Δ^12^‐PGJ_3_ (**3**) was achieved in 12 steps (longest linear sequence, LLS).

In conclusion, we have significantly improved the yield of our previously reported l‐proline catalyzed double aldol dimerization of succinaldehyde from 14 to 29 % for the synthesis of a key enal intermediate **1** that can be employed in the synthesis of a range of prostanoids. This has been achieved through a thorough re‐evaluation of all of the reaction parameters, which led us to make four key modifications of the reaction conditions: changing Me‐THF for EtOAc, changing dibenzylammonium trifluoroacetate **5** to thiomorpholinium trifluoroacetate **6**, the temperature of the second step from 25 °C to 65 °C, and the concentration has been decreased from 2 m to 0.75 m in the first step and from 1 m to 0.35 m in the second step. The synthesis, and the practical isolation and purification of enal **1** on a decagram scale has also been developed. Furthermore, we have exemplified the synthetic versatility our enal intermediate **1** through its application to the total synthesis of Δ^12^‐PGJ_3_ (**3**). This was achieved through an umpolung approach involving the conversion of the electrophilic enal moiety into an ene‐carbamate, which serves as a masked nucleophilic moiety.

## Conflict of interest

The authors declare no conflict of interest.

## Supporting information

As a service to our authors and readers, this journal provides supporting information supplied by the authors. Such materials are peer reviewed and may be re‐organized for online delivery, but are not copy‐edited or typeset. Technical support issues arising from supporting information (other than missing files) should be addressed to the authors.

Supplementary
