# Supplementary material for: Reoptimization of the Organocatalyzed Double Aldol Domino Process to a Key Enal Intermediate and Its Application to the Total Synthesis of Δ12‐Prostaglandin J3
Source: Chemistry. 2018 Jun 6;24(38):9542–5. doi: 10.1002/chem.201802498 (PMC6055629; doi:10.1002/chem.201802498)
Supplement: Supplementary file 1 — Supplementary [file CHEM-24-9542-s001.pdf]

CHEMISTRY
A European Journal

Supporting Information

Reoptimization of the Organocatalyzed Double Aldol Domino Process to a Key Enal Intermediate and Its Application to the Total Synthesis of Δ^{12}-Prostaglandin J_3

Andrejs Pelšs,[a] Narasimhulu Gandhamsetty,[a] James R. Smith,[a] Damien Mailhol,[a] Mattia Silvi,[a] Andrew J. A. Watson,[a] Isabel Perez-Powell,[a] Sébastien Prévost,[a] Nina Schützenmeister,[a] Peter R. Moore,[b] and Varinder K. Aggarwal*[a]

chem_201802498_sm_miscellaneous_information.pdf

Contents

1. General Information 2
2. Optimization Studies. 3
3. Detailed Synthetic Procedures for the Synthesis of Enal 1. 9
4. Experimental Procedures for the Total Synthesis of Δ^{12}-PGJ$_3$. 18
5. NMR Spectra 34
6. References 47

1. General Information

Anhydrous solvents were either dried using an Anhydrous Engineering alumina column drying system (THF, toluene, CH_2Cl_2) or obtained as Acroseal bottles and used directly (acetone), all other solvents used were reagent grade solvents and were used directly. Petroleum ether refers to the fraction collected between 40 – 60 °C. Reactions requiring anhydrous conditions (where specified) were conducted under a N_2 atmosphere using standard Schlenk techniques unless otherwise stated. All reagents were purchased from commercial sources and used as received, unless otherwise stated. 2,5-Dimethoxytetrahydrofuran (mixture of *cis*- and *trans*-isomers) was purchased from Acros Organics and was used as received. Morpholinium trifluoroacetate **6** (and all other analogous trifluoroacetate salts) was synthesised according to the literature procedure and the spectroscopic data were in agreement with the literature.[1] Methanesulfonyl chloride was purchased from Sigma-Aldrich and was distilled (Hickman apparatus) prior to use. Flash column chromatography was carried out using either Aldrich silica gel (40-63 μm) in a glass column, or using a Biotage Isolera™ Prime automated flash column chromatography system. All reactions were followed by thin-layer chromatography (TLC) when practical, using Merck Kieselgel 60 F254 fluorescent treated silica which was visualised under UV light (254 nm) or by staining with an aqueous basic potassium permanganate solution. A Mettler-Toledo OptiMax Synthesis Workstation was used for reactions conducted in a jacketed vessel. **^{1}H NMR** spectra were recorded using either Jeol ECS 400 MHz, Bruker 400 MHz, Bruker Cryo 500 MHz, or Varian VNMR (400 MHz or 500 MHz) spectrometers. Chemical shifts (δ) are given in parts per million (ppm) and coupling constants (*J*) are given in Hertz (Hz). **^{13}C NMR** spectra were recorded using either Varian VNMR 400 (101 MHz) or Bruker Cryo 500 (126 Hz) spectrometers. High resolution mass spectra (**HRMS**) were recorded on a Bruker Daltonics Apex IV by Electrospray Ionisation (ESI). **IR** spectra were recorded on a Perkin Elmer Spectrum One FT-IR as a thin film. Only selected absorption maxima (ν_{max}) are reported in wavenumbers (cm^{-1}). Melting points (**MP**) were recorded in degrees Celsius (°C), using a Stuart melting point apparatus (SMP3) and are reported uncorrected. Optical rotations ($[\alpha]_D^T$) were measured on a Bellingham and Stanley Ltd. ADO220 polarimeter, where *c* is given in g/100 mL. Chiral supercritical fluid chromatography (**SFC**) was performed using a Chiralpak® IA column (4.6 × 250 mm × 5μm) using a Waters TharSFC system and was monitored using a diode array detector (DAD). Chiral HPLC (**HPLC**) was performed on a HP Agilent 1100 using a Chiralpak® IC column (4.6 × 250 mm × 5μm) and was monitored using a diode array detector (DAD).

2. Optimization Studies.

We began our re-optimization by conducting a small solvent screen, employing the same reaction conditions as developed in a previous publication (Table S1, see entry 1 for our previously reported conditions).[2] Acetonitrile was identified as a slightly superior reaction solvent, providing enal **1** in a 16% NMR yield.

Table S1. Early solvent screen

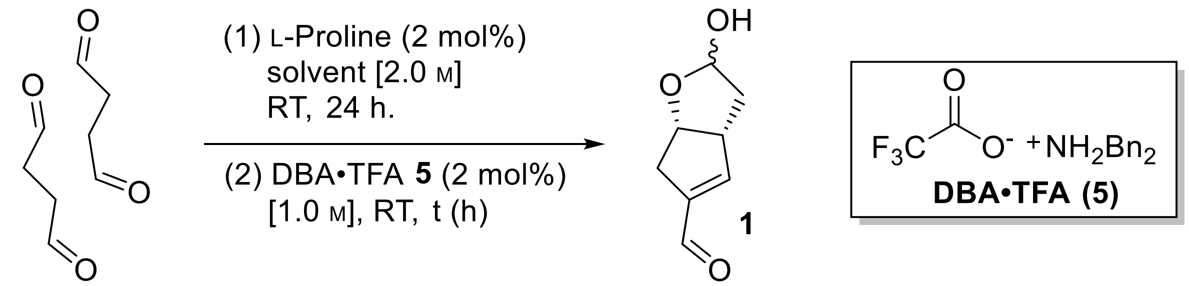

Entry	Solvent	t [h]	Yield [%][a]
1	THF	14	14
2	DMSO	20	0
3	MeCN	20	16
4	CH_3Cl	20	6

[a] Yield determined by ^{1}H NMR spectroscopy using 1,3,5-trimethoxybenzene as an internal standard.

We found that decreasing the reaction concentration for the first step from 2.0 M to 1.0 M further improved the NMR yield to 19% (Table S2, entry 1). However, due to the dibenzylamine catalyst **5** reacting with **1** during the isolation process, only a 9% isolated yield was achieved. This issue was alleviated by changing to thiomorpholine catalyst **6** for the second step of the reaction – this resulted in a 20% NMR and isolated yield (entry 2). Employing three further amine trifluoroacetate salts **A-C** did not improve the yield (entries 3-5).

Table S2. 2nd Catalyst Screen

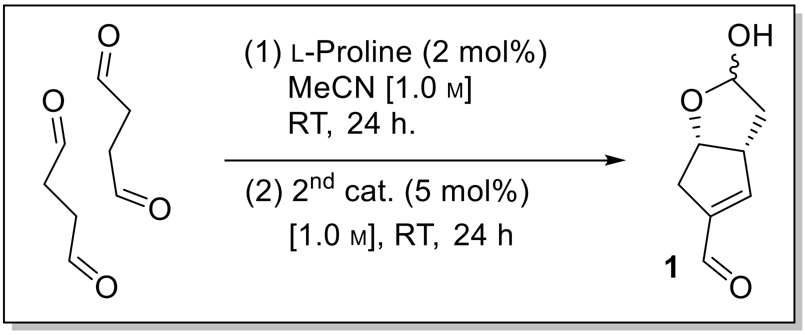

5
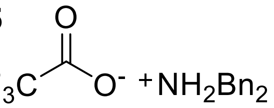

6
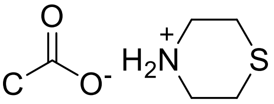

A
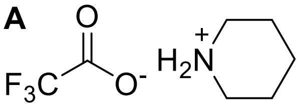

B
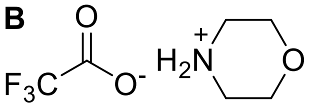

C
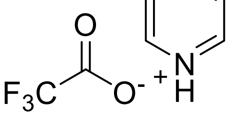

Entry	2nd Catalyst	Yield [%][a]
1	**5**	19 (9)
2	**6**	20 (20)
3	**A**	7
4	**B**	16
5	**C**	11

[a] Yield determined by ^{1}H NMR spectroscopy using 1,3,5-trimethoxybenzene as an internal standard – isolated yield shown in parentheses.

We then identified that the reaction can be improved by heating the reaction to 65 °C for the second step of the reaction – this allowed for a shorter reaction time for the second step (2 h) and a 23% NMR yield for **1** could be achieved when using acetonitrile as the reaction solvent (Table S3, entry 1). We conducted an extensive solvent screen with a second step temperature of 65 °C. Acetonitrile, THF, and 1,4-dioxane gave identical yields (Table S3, entries 3, 8 and 11). Using ethyl acetate as the reaction solvent provided a similar NMR yield (21%, entry 1), and a noticeable visual difference of the reaction mixture was observed. Typically large precipitation of oily oligomers is observed after the first step. However, less oligomeric precipitation was observed when using ethyl acetate as the solvent (see Figure S1). Notably, the oligomeric precipitate was a crystalline, water-soluble solid – we reasoned that this could allow for a simpler purification process at the end of the reaction. We therefore decided to use ethyl acetate as the reaction solvent in future optimization experiments.

Table S3. Extended solvent screen (65 °C for 2nd step)

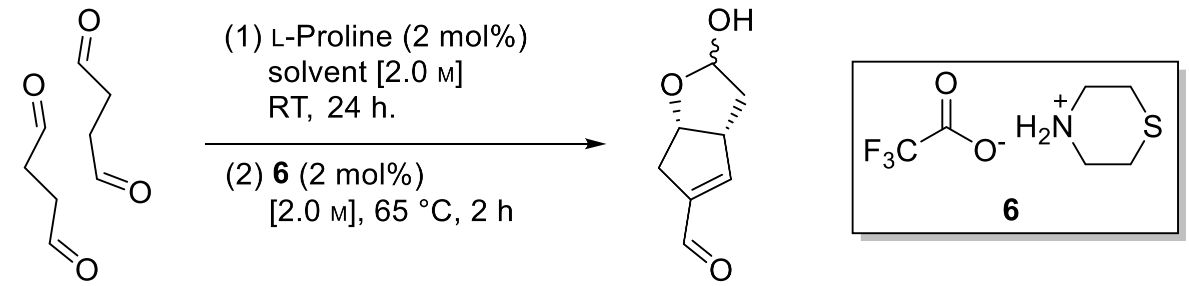

Entry	Solvent	Yield [%][a]	Succinaldehyde [%][b]
1	EtOAc	21	12
2	Et_2O	4	13
3	THF	23	6
4	CH_2Cl_2	19	13
5	CH_3Cl	16	13
6	*i*-PrOH	4	15
7	$MeNO_2$	22	6
8	Dioxane	23	5
9	1,2-DCE	18	15
10	*i*-Pr_2O	0	13
11	MeCN	23	6
12	DMF	17	3
13	DME	21	5
14	MTBE	6	13
15	2-MeTHF	19	7
16	Toluene	2	15

[a] Yield determined by ^1H NMR spectroscopy using 1,3,5-trimethoxybenzene as an internal standard. [b] Remaining succinaldehyde at the end of the reaction was determined by ^1H NMR spectroscopy using 1,3,5-trimethoxybenzene as an internal standard.

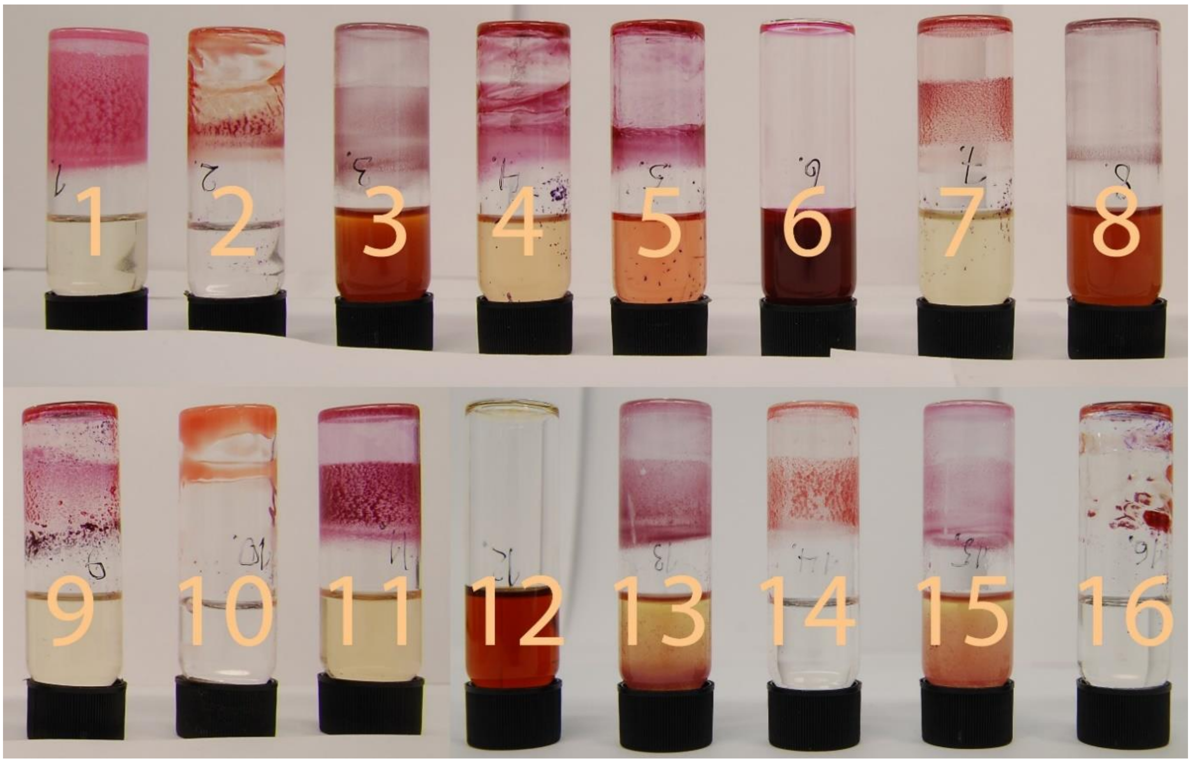

Figure S1. Images of inverted reaction vessels for the extended solvent screen after 20 h (first step) – the label numbers correspond to the entry numbers in Table S3.

The concentration of the first and second step of the reaction was explored next. The concentration of the first step was varied from 0.25 to 4.0 M using ethyl acetate as the reaction solvent, whilst using the same concentration for the second step (Table S4, entries 1-8). In general, the reaction yield improved as the reaction concentration was decreased, culminating in a 28% NMR yield being achieved with a 0.5 M reaction concentration (entry 2). However, further reducing the reaction concentration to 0.25 M only provided a 12% yield due to a poor conversion of succinaldehyde (entry 1). Again, there was a striking visual difference to the reaction mixtures at varying concentrations, with only minor yellow precipitate formation being observed at low concentrations, and large amounts of dark pink oligomeric precipitates forming at higher concentrations (see Figure S2). We saw further improvements to the reaction yield through dilution of the reaction mixture before the addition of the second catalyst for the second step from 0.5 M to 0.35 M (31%, entry 9). Finally, our optimal reaction conditions were obtained through increasing the concentration of the first step to 0.75 M, and decreasing the concentration of the second step to 0.2 M. This allowed for the formation enal **1** in a 33% NMR yield (entry 11).

Table S4. Concentration screen

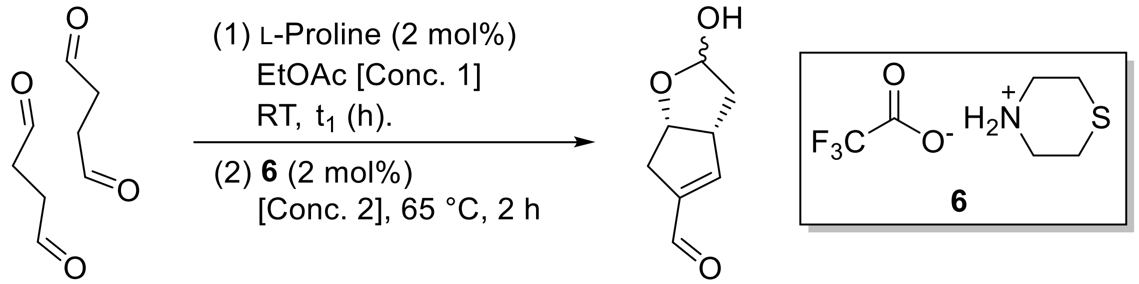

Entry	Conc. 1 [M]	Conc. 2 [M]	t_1 [h]	Yield [%][a]	Succinaldehdye [%][b]
1	0.25	0.25	24	12	40
2	0.5	0.5	24	28	19
3	0.75	0.75	24	25	15
4	1.0	1.0	24	26	12
5	1.5	1.5	24	24	11
6	2.0	2.0	24	21	11
7	3.0	3.0	24	18	8
8	4.0	4.0	24	14	7
9	0.5	0.35	40	31	22
10	0.75	0.35	40	32	17
11	0.75	0.2	40	33	17
12	2.0	0.35	47	29	16
13	1.0	0.35	47[c]	29	14

[a] Yield determined by ^{1}H NMR spectroscopy using 1,3,5-trimethoxybenzene as an internal standard. [b] Remaining succinaldehyde at the end of the reaction was determined by ^{1}H NMR spectroscopy using 1,3,5-trimethoxybenzene as an internal standard. [c] A second portion of L-proline (2 mol%) was added after 24 h.

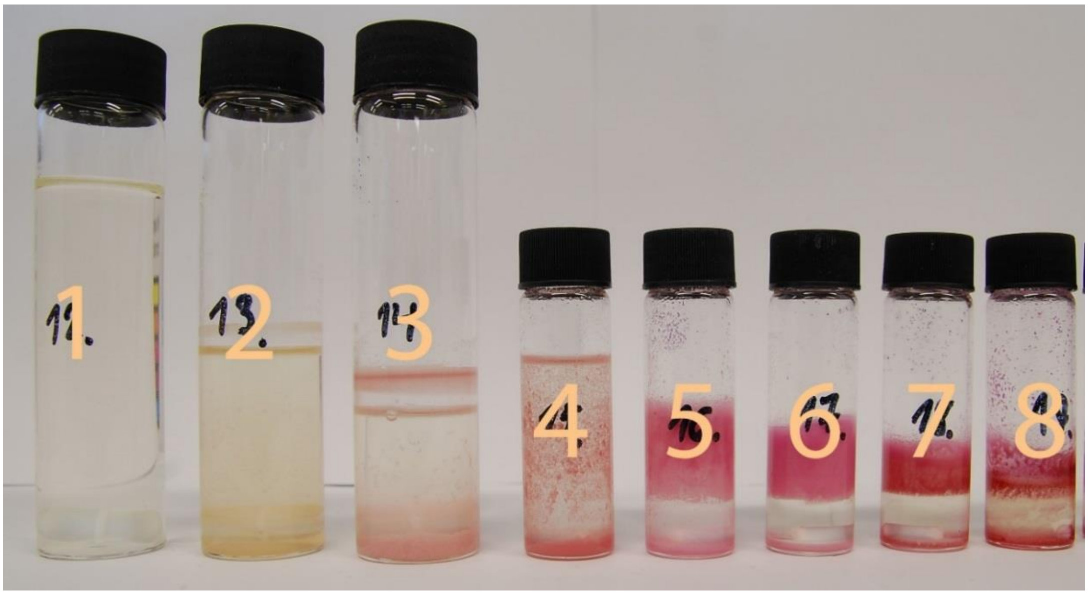

Figure S2. Images of reaction vessels for the concentration screen after 24 h (first step) – the label numbers correspond to the entry numbers in Table S4.

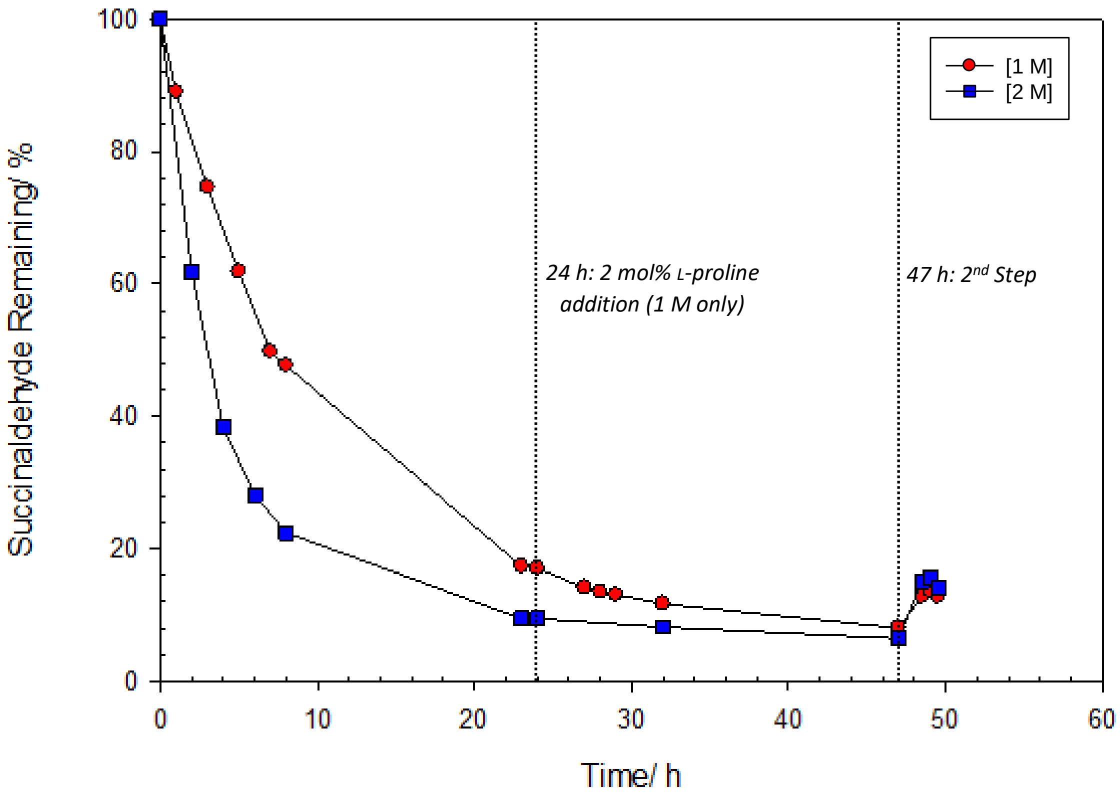

Figure S3. Succinaldehyde conversion versus time at 2 M (red points; Table S4, entry 12) and 1 M (blue points; Table S4, entry 13) reaction concentrations.

Our final optimization studies were aimed at determining the optimal time for the second step (Table S5). An aliquot of the reaction conducted under the optimized reaction conditions was removed at hourly time points for four hours, and then analyzed by 1H NMR. This revealed that the optimal reaction time for the second step is 2 hours, and that the reaction yield begins to drop after 3 hours.

Table S5. Effect of time for the 2nd step of the reaction

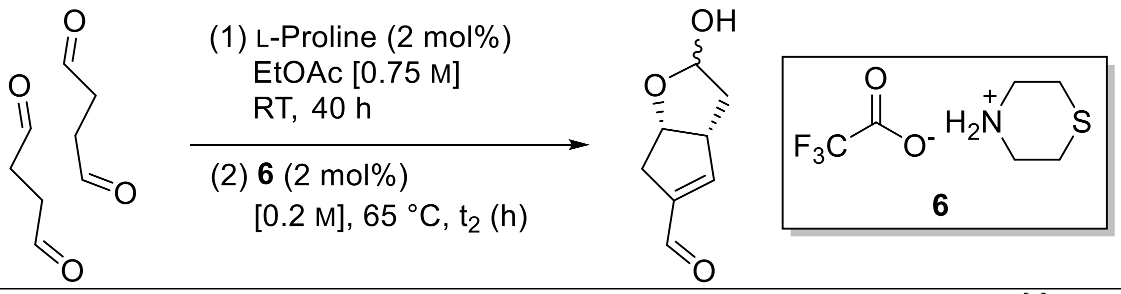

Entry	Time for Step 2 (t_2) [h]	Yield [%][a]
1	1	30
2	2	33
3	3	33
4	4	32

[a] Yield determined by ^{1}H NMR spectroscopy using 1,3,5-trimethoxybenzene as an internal standard.

3. Detailed Synthetic Procedures for the Synthesis of Enal 1.

We have previously reported the preparation of succinaldehyde on scales ranging from 5g-200g using standard lab glassware.[2] The procedure below was carried out in a jacketed Optimax vessel which enables greater control and monitoring of internal reaction temperature.

Succinaldehyde synthesis

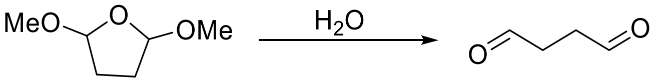

To a 1 L jacketed vessel, equipped with an overhead stirrer and a temperature probe, was added 2,5-dimethoxytetrahydrofuran (mixture of *cis* and *trans*-isomers, 200 mL, 1.55 mol), and H_2O (400 mL). The reaction mixture was stirred at 300 RPM and the jacket temperature was warmed to 115 °C, over a 10 min period – the reaction mixture (internal temperature) increased to 90 °C over a 40 min period, and the reaction was held[i] at this temperature for a further 2 h. The reflux condenser was moved into a distillation position, a 500 mL receiving flask was added and the flask was placed into an ice-bath.[ii] The jacket temperature was increased to 135 °C over a 15 min period, and the distillate[iii] was collected for 90 min.[iv] The jacket temperature was decreased to 90 °C and the pressure of the reaction vessel was reduced to ca. 100 mbar.[v] The distillation was continued until a further 190 mL of distillate was collected (430 mL in total).[vi] At this point, the pressure was returned to atmospheric pressure, toluene (200 mL) was added and the reaction temperature was warmed to 50 °C. The pressure was reduced to ca. 100 mbar and the reaction mixture was concentrated to remove the toluene (with concomitant azeotropic removal of water).[vii] This azeotropic removal of water with toluene was repeated twice more (2 x 200 mL), and the resultant yellow oil (ca. 100 g) was transferred to a 500 mL round bottom

[i] The jacket temperature was maintained at 115 °C.
[ii] All exposed (i.e. not covered by the heated jacket) hot surfaces were insulated with cotton wool and aluminium foil to ensure a consistent distillation.
[iii] This distillate contains a mixture of H_2O and MeOH, generated from the reaction – see Figure S4 for set-up.
[iv] The temperature of the reaction slowly increased to ca. 100 °C during this time, and a minimum of 240 mL distillate was collected – if this volume was not achieved, then wait for a further 30 min (or until this volume has been achieved).
[v] The reduction of pressure should be conducted in a controlled and gradual manner to ensure the reaction mixture does not excessively bump – once the pressure has been reduced to ca. 100 mbar, the reaction temperature will drop to ca. 40 °C.
[vi] This usually requires a ca. 2 h distillation time (reaction scale dependent).
[vii] The reaction temperature dropped to ca. 40 °C at this reduced pressure, and the jacket temperature increased to ca. 95 °C – when no further distillate was being produced, the reaction temperature increased back to 50 °C, indicating that the distillation was complete.

flask for purification by distillation.[viii] Distillation under reduced pressure (38-40 °C at 1.5 mbar)[ix] afforded succinaldehyde as a colourless oil (79.8 g, 60% yield).

^{1}H NMR ($CDCl_3$, 400 MHz) δ (ppm) 9.81 (s, 2H), 2.80 (s, 4H).

^{13}C NMR ($CDCl_3$, 101 MHz) δ (ppm) 199.7, 35.9.

Analytical data were consistent with those reported in the literature.[2]

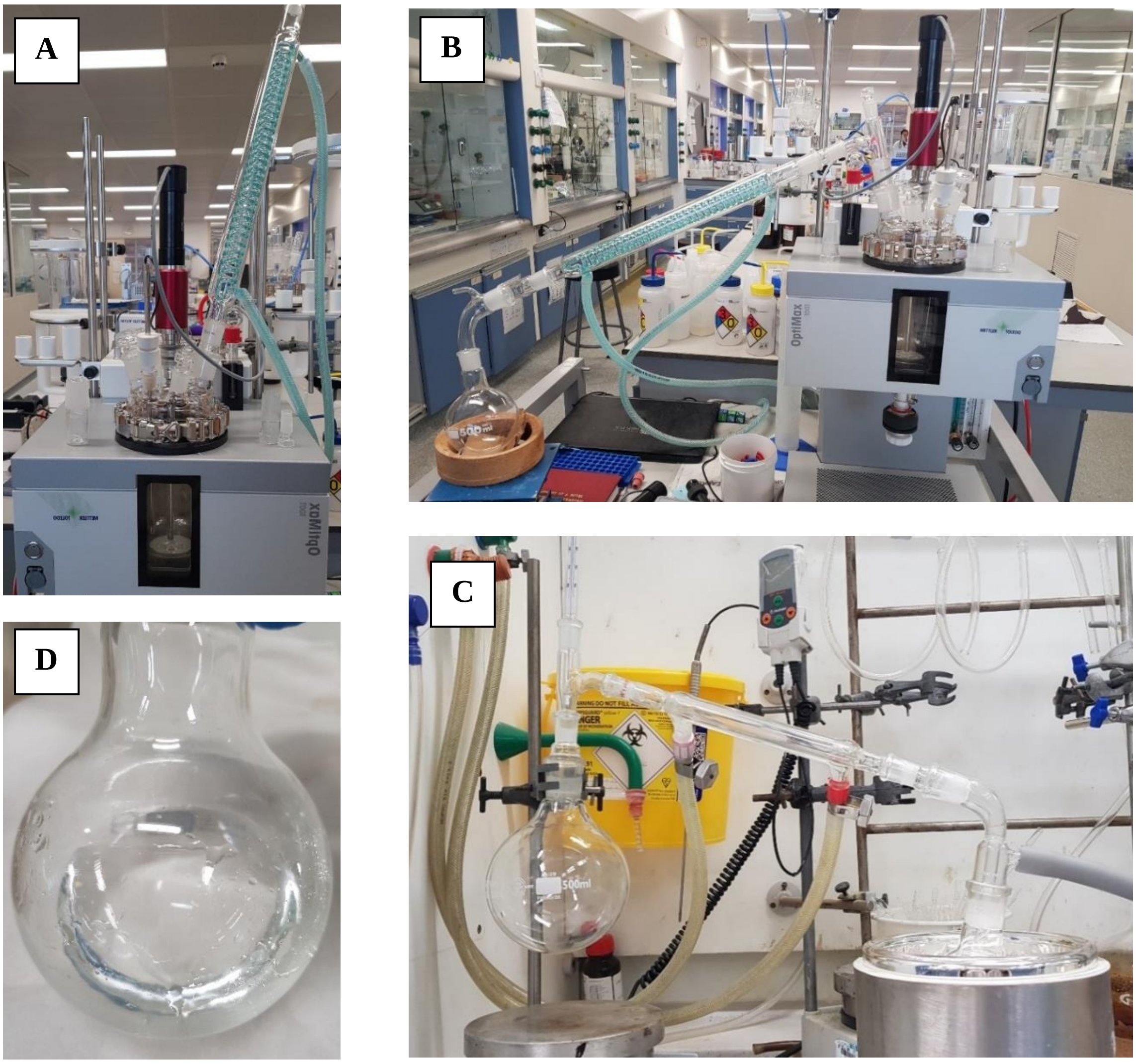

Figure S4. Images from the succinaldehyde synthetic procedure: A) Jacketed vessel equipped with overhead stirrer and temperature probe (no reaction present in image). B) Jacketed vessel with distillation set-up (minus cotton wool and foil insulation). C) Succinaldehyde distillation set-up (minus oil bath and foil). D) Freshly distilled succinaldehyde appearance.

[viii] The crude reaction mixture containing succinaldehyde can be stored in a freezer as a solution in CH_2Cl_2 (ca. 4 mL g^{-1}) for 2-3 weeks. The CH_2Cl_2 solution should be concentrated to dryness on a rotary evaporator prior to distillation of the succinaldehyde.

[ix] The oil bath temperature was set to 75 °C. The hot surfaces were covered with aluminium foil to ensure an efficient distillation. The receiving flask was cooled in a dry ice/acetone bath. At this temperature the succinaldehyde is collected as a colourless solid – at the end of the distillation the ice bath was removed, and the receiving flask was allowed to naturally warm to RT under reduced pressure (0.1 mbar).

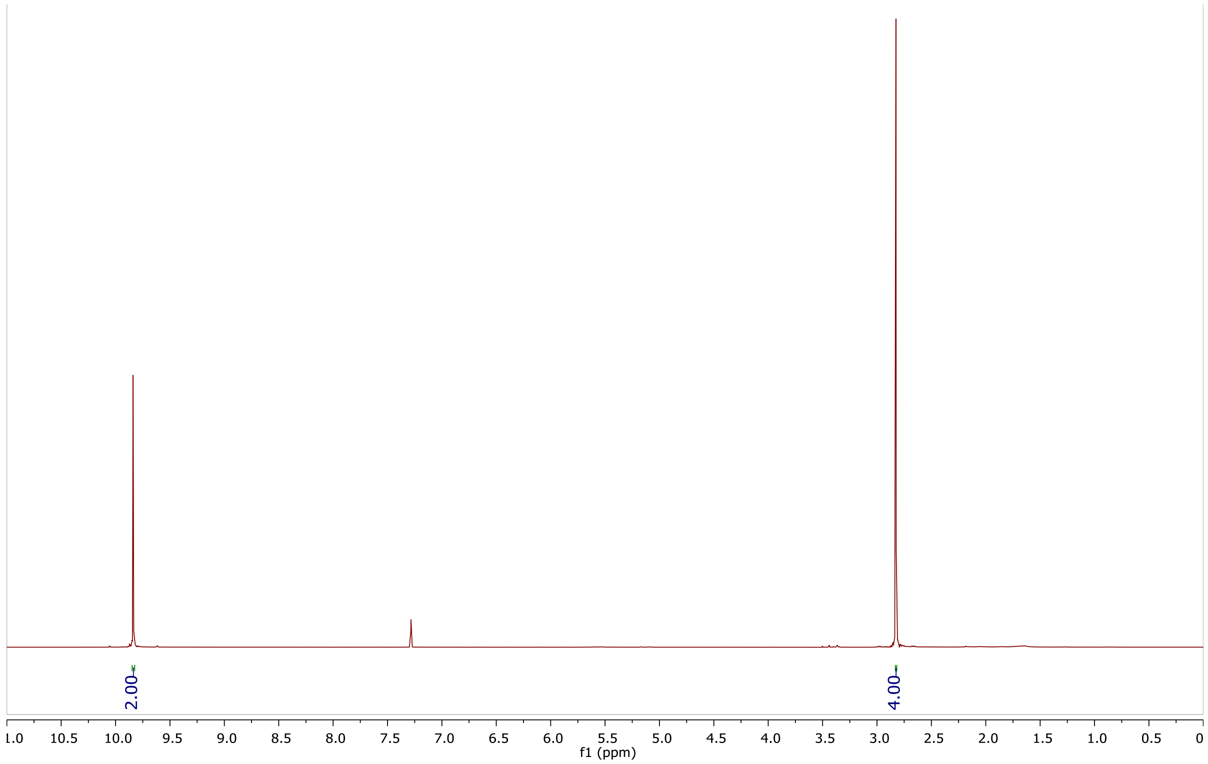

Figure S5. ^{1}H NMR spectrum of freshly distilled succinaldehyde (good quality).

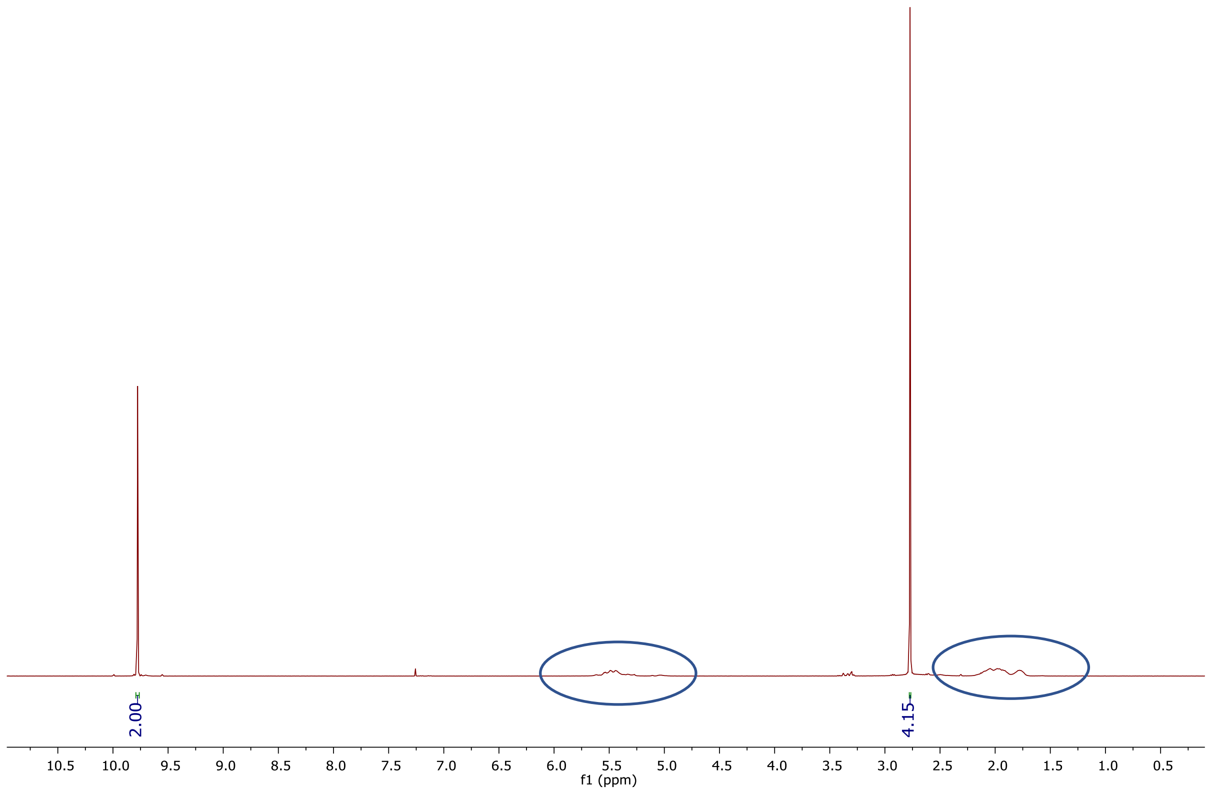

Figure S6. ^{1}H NMR spectrum of freshly distilled succinaldehyde (poor quality – oligomer formation observed, not suitable for use in subsequent aldol [signals highlighted with elipsoids]).

500 mg Scale Enal 1 Synthesis

(3aR,6aS)-2-hydroxy-3,3a,6,6a-tetrahydro-2H-cyclopenta[b]furan-5-carbaldehyde (1)

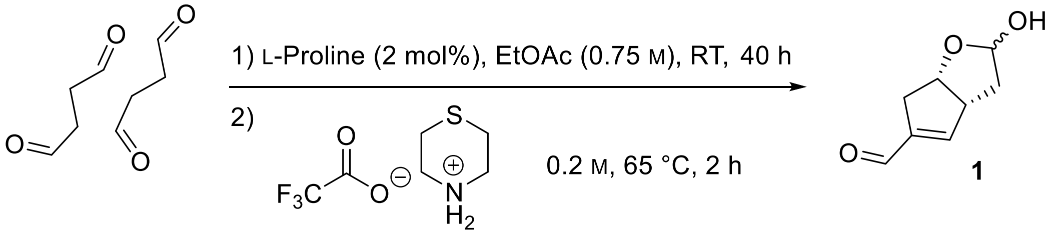

To a 50 mL round bottom flask equipped with a small (12 × 4.5 mm) stirrer bar was added freshly distilled succinaldehyde (500 mg, 5.81 mmol). Ethyl acetate (7.8 mL, 0.75 M) was added and the mixture was briefly stirred (ca. 30 seconds) to ensure full dissolution of the succinaldehyde.[x] To the reaction mixture was added 1,3,5-trimethoxybenzene (internal standard, 24.4 mg, 0.145 mmol, 2.5 mol%) followed by L-proline (13.4 mg, 0.116 mmol, 2 mol%). The reaction mixture was stirred at 700 rpm for 40 h at room temperature.[xi] An aliquot of the reaction mixture obtained at this point was analysed by ^{1}H NMR to show that 17% succinaldehyde remained in the reaction mixture.[xii] The reaction mixture was diluted to 0.2 M by addition of EtOAc (21.3 mL). Thiomorpholine trifluoroacetate (25.2 mg, 0.116 mmol, 2 mol%) was added to the reaction, the reaction vessel was placed into a pre-heated (65 °C) oil bath and the reaction was stirred at 700 rpm for 2 h at this temperature.[xiii] An aliquot of the reaction mixture obtained at this point showed a 33% NMR yield (as an approximately 2:1 mixture of diastereomers) for enal **1**.[xii] The reaction mixture was removed from the oil bath and the reaction was allowed to cool to RT. An aq. Na_2SO_4 (15 mL, 17% *w*/*w*) solution was added and the biphasic mixture was stirred vigorously for 10 min. The biphasic mixture was transferred to a 100 mL separating funnel and the phases were separated. The aqueous phase was extracted with EtOAc (2 × 30 mL) and the combined organic layers (29 mL + 2 × 30 mL) were dried ($MgSO_4$), filtered and concentrated *in vacuo* to dryness. The crude reaction mixture was purified by flash column chromatography using a Biotage automated chromatography

[x] It is essential to ensure full dissolution of succinaldehyde before the addition of L-proline – the formation of pink/purple oligomers is rapidly observed if L-proline is added to a non-homogeneous mixture of ethyl acetate and succinaldehyde.

[xi] Typically, 23-25 °C.

[xii] An aliquot of ca. 50 µL was added to a standard NMR tube and was diluted with $CDCl_3$ (0.5 mL). A ^{1}H NMR spectrum of this solution was obtained using a Varian 400 MHz NMR spectrometer (25 °C, 8 scans, 30 ° pulse angle, 30 s relaxation delay). The integration of the aromatic signal corresponding to 1,3,5-trimethoxybenzene [6.05 (3H, s)] was compared with the integration of either the aldehydic signal corresponding to succinaldehyde [9.78 (2H, s)] or the combined integration of the alkenyl signals corresponding to the two diastereoisomers of enal **1** [6.79 (1H, q) 6.64 (1H, q)], to quantify the amount of succinaldehyde and enal **1**, respectively.

[xiii] During this heating period, the reaction will turn from light pink, through to purple, and will end as a dark brown heterogeneous mixture – see Figure S10.

machine: the crude reaction mixture was added to a pre-equilibrated column (50:50 EtOAc:pentane, 30 g Biotage ZIP® cartridge) with CH_2Cl_2 (3 × 2 mL) and the following eluent program was used: 1 column volume (CV) isocratic (50:50 EtOAc:pentane); 10 CV gradient (50:50 to 90:10 EtOAc:pentane); 2 CV isocratic (90:10 EtOAc:pentane). The compound began eluting from the column at an eluent strength of ca. 70:30 EtOAc:pentane. The title compound **1** was afforded as a light brown solid (138 mg, 31% yield, as an approximately 2:1 mixture of diastereomers).

R_f: 0.16 (pentane/EtOAc, 50:50).

^{1}H NMR ($CDCl_3$, 400 MHz) δ (ppm) 9.76 (s, 1H*), 9.75 (s, 1H), 6.78 (q, *J* = 2.0 Hz, 1H*), 6.64 (q, *J* = 2.0 Hz, 1H), 5.57 – 5.48 (m, 1H + 1H*), 4.95 (td, *J* = 6.0, 1.7 Hz, 1H), 4.88 (dt, *J* = 7.0, 3.9 Hz, 1H*), 3.70 – 3.61 (m, 1H), 3.61 – 3.50 (m, 1H*), 3.29 (br. s, 1H), 3.09 (br. s, 1H*), 2.82 – 2.62 (m, 2H + 2H*), 2.24 (ddd, J = 13.3, 9.9, 1.5 Hz, 1H), 2.19 (ddd, J = 9.5, 4.7, 1.5 Hz, 1H*), 2.08 (d, *J* = 13.2 Hz, 1H*), 1.93 (dt, *J* = 13.3, 5.1 Hz, 1H). *The signals corresponding to the minor diastereomer are highlighted with an asterisk (*).*

Major diastereoisomer: **^{13}C NMR** (DMSO-d_6, 126 MHz, 80 °C) δ (ppm) 190.0, 152.4, 144.7, 98.5, 80.8, 49.4, 37.88, 35.4.

Minor diastereoisomer: **^{13}C NMR** (DMSO-d_6, 126 MHz, 80 °C) δ (ppm) 190.1, 153.2, 144.7, 98.9, 82.8, 50.0, 38.1, 37.92.

Analytical data were consistent with those reported in the literature.[2-3]

50 g Scale Enal 1 Synthesis

To a 2 L round bottom flask equipped with a large (40 × 8 mm) stirrer bar was added freshly distilled succinaldehyde (50.0 g, 581 mmol). Ethyl acetate (774 mL, 0.75 M) was added and the mixture was briefly stirred (ca. 30 seconds) to ensure full dissolution of the succinaldehyde.[x] To the reaction mixture was added 1,3,5-trimethoxybenzene (internal standard, 2.44 g, 14.5 mmol, 2.5 mol%) followed by L-proline (1.34 g, 11.6 mmol, 2 mol%). The reaction mixture was stirred at 700 rpm for 40 h at room temperature.[xi] An aliquot of the reaction mixture obtained at this point was analysed by ^{1}H NMR to show that 16% succinaldehyde remained in the reaction mixture.[xii] The reaction mixture was diluted to 0.35 M by addition of EtOAc (885 mL). Thiomorpholine trifluoroacetate (2.52 g, 11.6 mmol, 2 mol%) was added to the reaction, and the reaction vessel was placed into a pre-heated (68 °C) oil bath. The reaction mixture reached 65 °C after ca. 20 min and the reaction was then stirred for a further 2 h at this temperature.[xiii] An aliquot of the reaction mixture obtained at this point showed a 32% NMR yield (as an approximately 2:1 mixture of diastereomers; 19% succinaldehyde remaining) for enal **1**.[xii] The reaction mixture was removed from the hot oil bath and was quickly cooled to 30 °C with an ice bath. The ice bath was removed, and the reaction was transferred to a 3 L conical flask equipped with a large stirrer bar. An aq. Na_2SO_4 (1.0 L, 17% *w*/*w*)[xiv] solution was added to the reaction mixture, the biphasic mixture was stirred vigorously for 10 min, and the mixture was transferred to a 5 L separating funnel. The phases were separated, and the aqueous phase was returned to the 3 L conical flask. This extraction process was repeated twice more (each with 10 min vigorous stirring) to further extract the aqueous phase with with EtOAc (2 x 1.0 L), and the combined organic layers (1.66 L + 2 × 1.0 L) were dried ($MgSO_4$), filtered and concentrated *in vacuo* to a ca. 1 L volume.[xv] Pre-treated wet silica[xvi] (100 g SiO_2 + 75 mL H_2O) was added, and the mixture was stirred vigorously for 30 mins. The mixture was filtered through a Buchner funnel equipped with filter paper – the silica was washed with EtOAc (2 x 250 mL) and the combined mother liquors were concentrated *in vacuo* to dryness.[xvii] The crude reaction mixture was further

[xiv] The aq. Na_2SO_4 (17% *w*/*w*) solution was prepared by adding $Na_2SO_4{\cdot}10H_2O$ (385 g) portionwise to vigorously stirred H_2O (785 mL). The stirring was continued until complete dissolution of Na_2SO_4 was observed – gentle heating was required.

[xv] An aliquot of the combined organic phases was analysed by ^{1}H NMR to reveal that 7% succinaldehyde was remaining following the aqueous work up.

[xvi] The pre-treated wet silica was prepared by adding water to the dry silica in a sealable container – the container was closed with a lid, and the mixture was shaken vigorously until the consistency of the wet silica became uniform, and the silica was then left to stand for 1 h.

[xvii] An aliquot of the combined organic phases was analysed by ^{1}H NMR to reveal that 4% succinaldehyde was remaining following the silica filtration.

purified by flash column chromatography using a Biotage automated chromatography machine. The crude reaction mixture added to a pre-equilibrated column (50:50 EtOAc:pentane, wet silica[xvi] [300 g SiO_2 + 150 mL H_2O])[xviii] with CH_2Cl_2 (3 × 30 mL) and the following eluent program was used: 1 column volume (CV) isocratic (50:50 EtOAc:pentane); 8 CV gradient (50:50 to 90:10 EtOAc:pentane); 1 CV isocratic (90:10 EtOAc:pentane). Enal **1** began eluting from the column at an eluent strength of ca. 70:30 EtOAc:pentane and was afforded as a brown solid (12.8 g, 29% yield, as an approximately 4:1 mixture of diastereomers, 99:1 er).

The er was determined following a Stahl[4] aerobic oxidation of purified lactol ***1*** *to the corresponding lactone* ***S1*** *and subsequent analysis by chiral SFC. Lactone* ***S1*** *was prepared according to the literature and the characterisation data matched the literature.[3]*

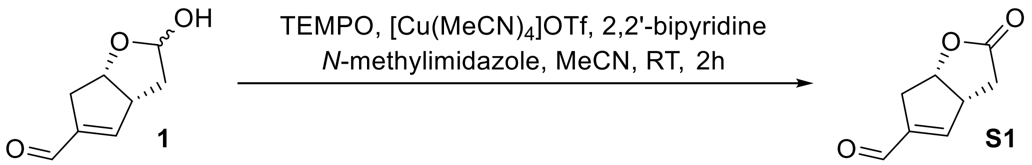

Chrial SFC Conditions: Chiralpak® IA, 125 bar, 40 °C, 4 ml min^{-1}, 5% co-solvent (*i*-PrOH); t_R (minor) = 7.3 min, t_R (minor) = 8.7 min; er = 99.1:0.9.

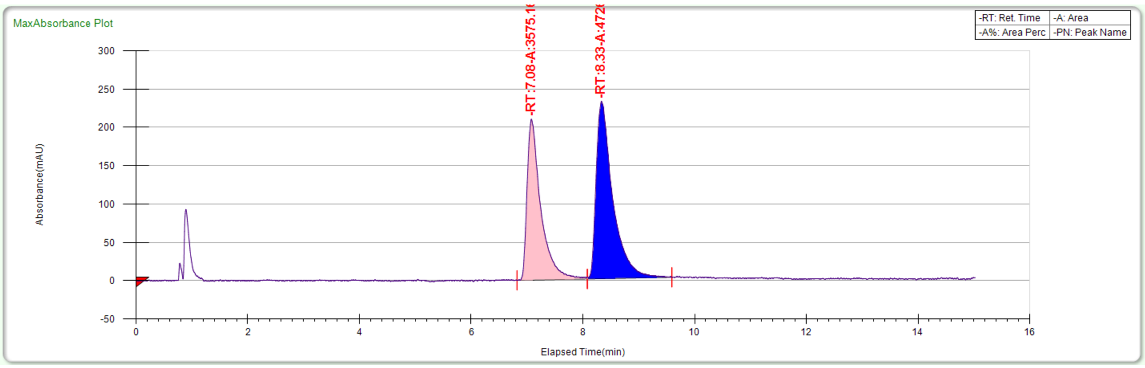

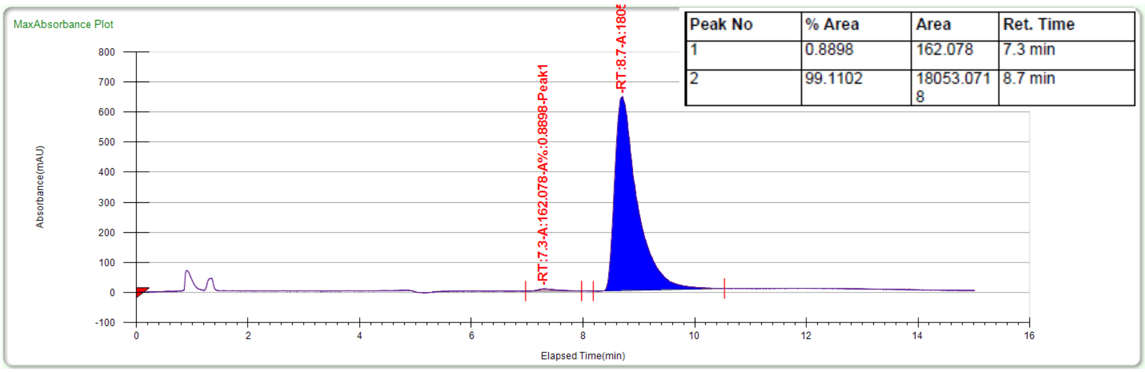

Peak No	% Area	Area	Ret. Time
1	0.8898	162.078	7.3 min
2	99.1102	18053.071 8	8.7 min

[xviii] The pre-treated wet silica was manually packed into an emptied 340 g Biotage SNAP® KP-SIL cartridge.

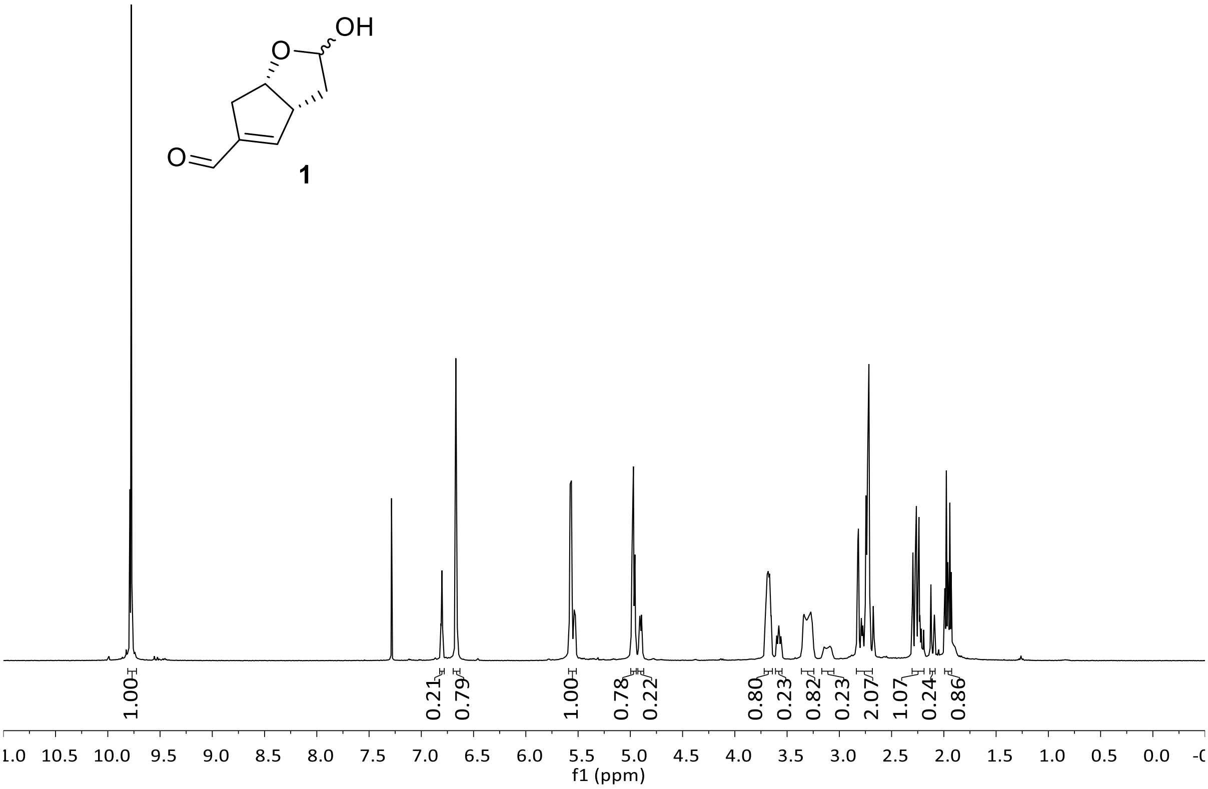

Figure S7. ^{1}H NMR of Enal **1** isolated from a 50 g scale reaction.

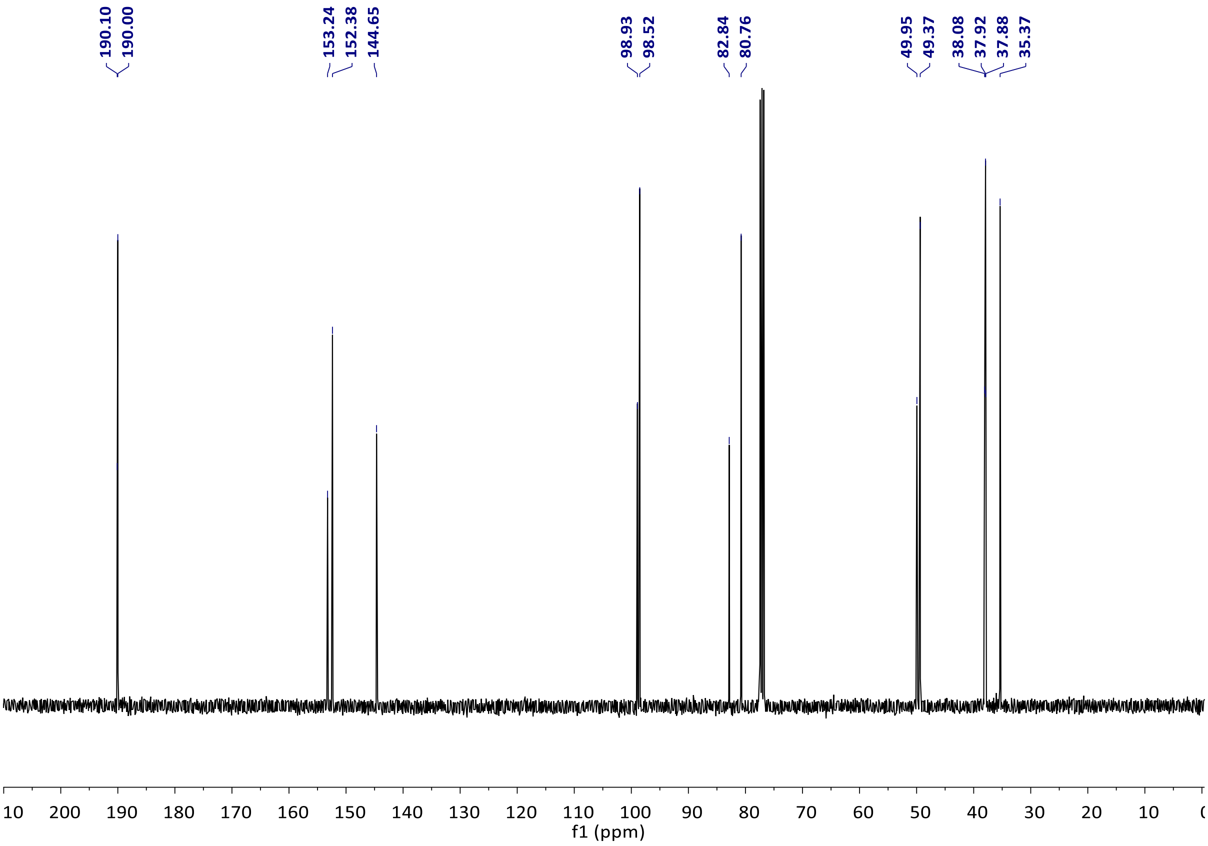

Figure S8. ^{13}C NMR of Enal **1** isolated from a 50 g scale reaction.

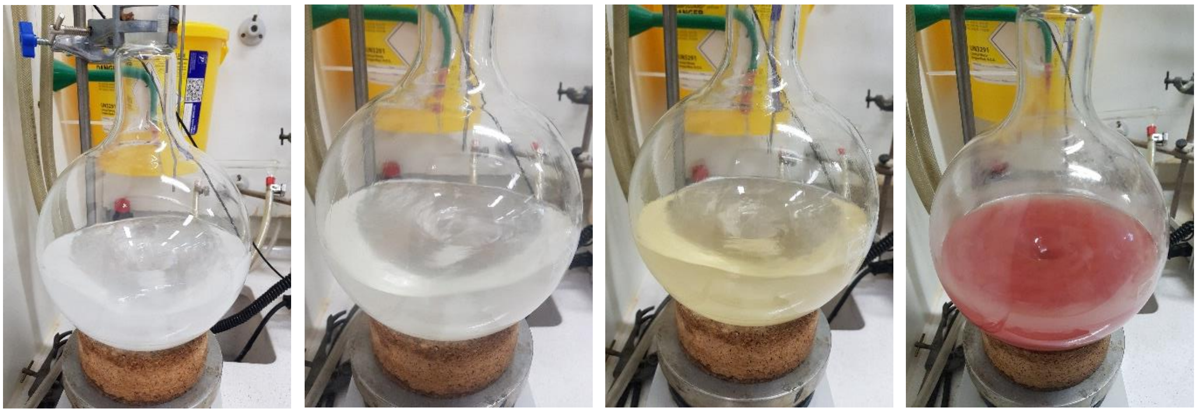

0 h 4 h 8 h 40 h

Figure S9. Images of the 1st step of the Aldol domino process for the synthesis of enal **1** – time labels indicate the time elapsed since the start of the reaction.

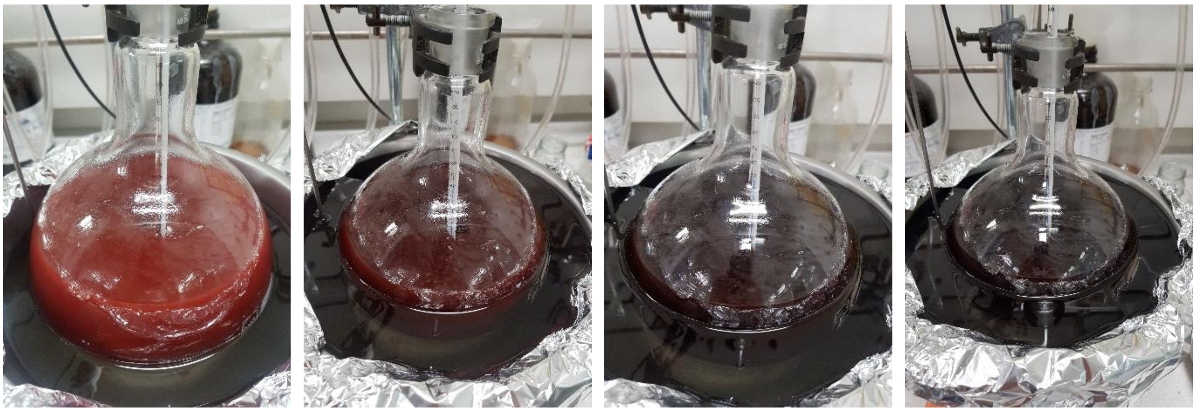

15 min 30 min 45 min 1 h

Figure S10. Images of the 2nd step of the Aldol domino process for the synthesis of enal **1** – time labels indicate the time elapsed since the reaction was added to the pre-heated oil bath (N.B. there were no further visual changes after 1 h of heating).

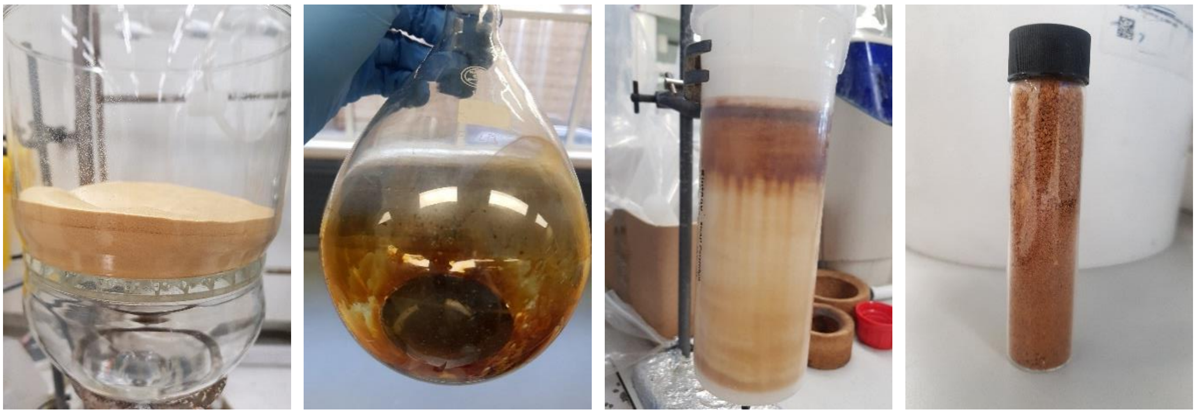

A B C D

Figure S11. Images illustrating the purification process for the synthesis of enal **1**. A) Large sinter funnel containing silica (yellow/ brown colorization due to adherance of oligomers) from the filtration of the crude reaction mixture following treatment with wet silica and B) the resultant viscous dark brown oil obtained after concentrating the mother liquour to dryness. C) Visual appearance of the silica gel column after being air-purged following purification of the crude reaction mixture – a clear visible dark brown band caused by adherance of oligomeric material can be observed at the top of the column. D) Visual appearnce of enal **1** (brown solid).

4. Experimental Procedures for the Total Synthesis of Δ¹²-PGJ₃.

(3a*R*,6a*S*)-2-oxo-3,3a,6,6a-tetrahydro-2*H*-cyclopenta[*b*]furan-5-carboxylic acid (13).

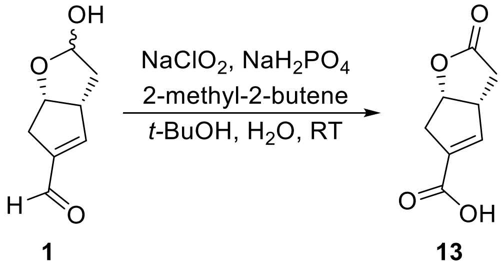

Enal **1** (7.00 g, 45.4 mmol, 1.0 eq.) was dissolved in *t*-BuOH (700 mL) and 2-methyl-butene (200 mL) was added. $NaClO_2$ (80% technical grade, 46.2 g, 409 mmol, 9.0 equiv.) and NaH_2PO_4 (37.6 g, 313 mmol, 6.9 equiv.) were dissolved in water (300 mL), transferred to an addition funnel and added dropwise to the enal solution with intense stirring (On large scales, an exothermic reaction is observed and so addition speed should be carefully controlled and cooling with an ice water bath should be applied if necessary to avoid excessive boiling of 2-methyl-butene). The reaction was stirred for 2 h at room temperature, *t*-BuOH was removed with rotary evaporation, and the aqueous residue was extracted with EtOAc (2 × 150 mL). The aqueous layer was acidified with 3M HCl to pH~3 and extracted with EtOAc (4 × 200 mL). The combined organic layers were washed with brine, dried ($MgSO_4$), filtered, and concentrated *in vacuo* to give acid **13** (5.64 g, 74%) as an off-white solid.

^{1}H NMR (DMSO-d_6, 400 MHz) δ (ppm) 12.58 (s, 1H), 6.52 (dt, *J* = 3.1, 1.7 Hz, 1H), 5.15 (t, *J* = 5.8 Hz, 1H), 3.68 (ddp, *J* = 9.6, 5.6, 1.8 Hz, 1H), 2.94 – 2.81 (m, 2H), 2.65 (d, *J* = 18.2 Hz, 1H), 2.50 (dd, *J* = 18.0, 1.7 Hz, 1H).

^{13}C NMR (DMSO-d_6, 101 MHz) δ (ppm) 176.5, 165.8, 142.7, 135.3, 83.1, 46.5, 38.7, 32.3.

$[\alpha]_D^{24}$: −86° (*c* 1.00, MeOH).

HRMS: (ESI) calculated for $C_8H_7O_4$ $(M-H)^-$ requires: 167.0344, found: 167.0355.

IR: (film) ν_{max}/cm^{-1}: 2958, 2929, 2857, 1783, 1729, 1462, 1252, 1181, 1027, 835, 774.

(3a*R*,6a*S*)-2-oxo-3,3a,6,6a-tetrahydro-2*H*-cyclopenta[*b*]furan-5-carbonyl azide (14).

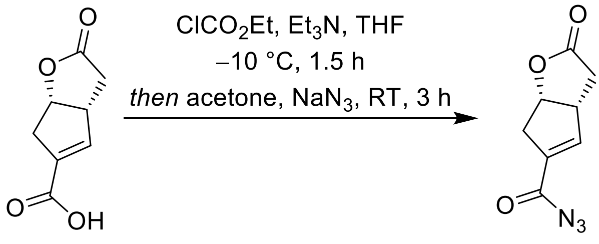

Acid **13** (1.45 g, 8.66 mmol, 1.0 equiv.) was dissolved in dry THF (50 ml) and Et_3N (2.42 mL, 17.3 mmol, 2.0 equiv.) was added dropwise at room temperature in a round bottom flask equipped with a large stirrer bar to enable efficient stirring of the heterogeneous mixture. The resulting solution was cooled to −15 °C bath temperature (ice/NaCl mixture) and ethyl chloroformate (1.24 mL, 13.0 mmol, 1.5 equiv.) was added dropwise with intense stirring. Precipitation occurred, and the reaction colour changed to pale orange-red. After 1.5 h, NaN_3 (1.41 g, 21.7 mmol, 2.5 equiv.) was added followed by dry acetone (50 ml). The heterogeneous mixture was stirred for 3 h, during which time the cooling bath naturally reached 15 °C. The reaction was quenched with water (100 mL) and EtOAc (100 mL) was added leading to complete dissolution of solids. The aqueous phase was further extracted with EtOAc (4 × 50 mL). The combined organic phases were washed with water (200 mL) and brine (200 mL), dried ($MgSO_4$), filtered, and concentrated to give a brown solid (1.39 g). The solid was decolorized from a brown impurity by a short silica gel plug filtration using 70% EtOAc/petrol ether to give azide **14** (1.36 g, 82%) as a pale-yellow solid.

^{1}H NMR ($CDCl_3$, 400 MHz) δ (ppm) 6.65 – 6.60 (m, 1H), 5.18 (ddd, J = 6.6, 5.4, 1.5 Hz, 1H), 3.77 – 3.65 (m, 1H), 3.05 – 2.90 (m, 2H), 2.85 (dd, J = 18.1, 10.3 Hz, 1H), 2.53 (dd, J = 18.2, 2.0 Hz, 1H).

^{13}C NMR ($CDCl_3$, 101 MHz) δ (ppm) 175.1, 169.4, 143.7, 136.9, 82.3, 46.5, 38.3, 32.1.

$[\alpha]_D^{24}$: −84° (*c* 1.00, $CHCl_3$).

IR: (film) ν_{max}/cm^{-1}: 2936, 2130, 1771, 1669, 1355, 1285, 1201, 1169, 929, 718.

Benzyl ((3a*R*,6a*S*)-2-oxo-3,3a,6,6a-tetrahydro-2*H*-cyclopenta[*b*]furan-5-yl)carbamate (12).

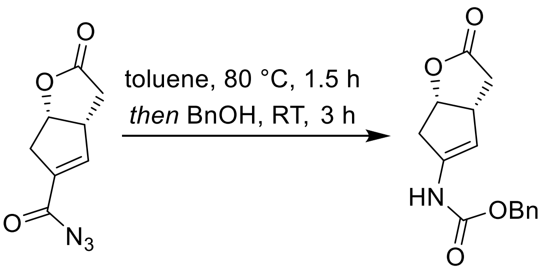

14 **12**

Azide **14** (3.25 g, 16.8 mmol, 1.0 equiv.) was dissolved in dry toluene (50 mL) and heated to 80 °C under an argon atmosphere. Slow gas evolution was observed. After 1.5 h, the reaction was cooled to room temperature and toluene was removed with rotary evaporation.[xix] Benzyl alcohol (8.70 mL, 84.1 mmol, 5.0 equiv.) was added to the resulting oily isocyanate with intense stirring to obtain a homogenous viscous pale-yellow oil, which solidified after 30 min. The oily solids were further aged for 2 h and directly purified using silica gel chromatography to remove residual benzyl alcohol to give lactone **12** (4.13 g, 90%) as a colourless solid.

^{1}H NMR (CD_2Cl_2, 500 MHz) δ (ppm) 7.47 – 7.27 (m, 5H), 6.55 (s, 1H), 5.46 (s, 1H), 5.16 (s, 2H), 5.04 (t, J = 5.9 Hz, 1H), 3.64 – 3.55 (m, 1H), 3.04 – 2.91 (m, 1H), 2.83 – 2.66 (m, 2H), 2.42 (dd, J = 17.9, 1.6 Hz, 1H).

^{13}C NMR (CD_2Cl_2, 126 MHz) δ (ppm) 176.6, 153.1, 136.1, 135.0, 128.5, 128.2, 128.1, 107.6, 80.9, 67.0, 44.1, 40.1, 34.1.

$[\alpha]_D^{24}$: −22.6° (*c* 1.00, CH_2Cl_2).

MP: 143-145 °C

HRMS: (ESI) calculated for $C_{15}H_{15}NO_4Na$ $(M+H)^+$ requires: 296.0893, found: 296.0905.

IR: (film) ν_{max}/cm^{-1}: 3328, 3034, 2955, 1700, 1605, 1534, 1454, 1403, 1342, 1259, 1182, 1048, 1027, 910.

[xix] It is crucial to remove toluene before benzyl alcohol addition to enable complete and fast conversion of the isocyanate to ene-carbamate **12**.

Benzyl ((3a*R*,6a*S*)-2-hydroxy-3,3a,6,6a-tetrahydro-2*H*-cyclopenta[*b*]furan-5-yl)carbamate (11)

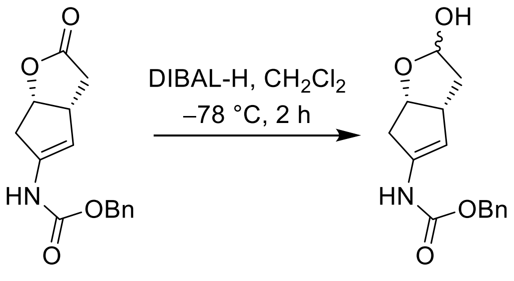

Ene-carbamate **12** (3.80 g, 13.9 mmol, 1.0 eq.) was dissolved in dry CH_2Cl_2 (75 mL) and cooled to −78 °C. DIBAL-H solution in CH_2Cl_2 (1 M, 30.6 mL, 30.6 mmol, 2.2 equiv.) was added dropwise immediately.[xx] After 2 h the reaction was quenched at −78 °C with MeOH (20 mL) and the cooling bath was removed. A saturated aqueous Rochelle salt solution (100 mL) and water (50 mL) were added and the mixture was stirred at maximum speed (1200 rpm) for 1 h, allowing the mixture to reach room temperature. The phases were separated, and the aqueous phase was further extracted with CH_2Cl_2 (3 × 75 mL). The combined organic phases were dried ($MgSO_4$), filtered, and concentrated *in vacuo* to give an inseparable diastereomeric mixture of lactol **11** (3.75 g, 98%; dr: 65:35) as a yellow oil.

^{1}H NMR (DMSO-d_6, 500 MHz, 80 °C) δ (ppm) 9.05 (s, 1H), 9.02 (s, 1H*), 7.59 – 7.20 (m, 5H + 5H*), 5.78 (d, *J* = 4.8 Hz, 1H), 5.63 (d, *J* = 5.1 Hz, 1H*), 5.40 (s, 1H*), 5.37 – 5.33 (m, 2H), 5.33 – 5.27 (m, 1H*), 5.11 (s, 2H + 2H*), 4.62 (t, *J* = 6.3 Hz, 1H), 4.53 (t, *J* = 1.5 Hz, 1H*), 3.38 – 3.29 (m, 1H), 3.28 – 3.17 (m, 1H*), 2.75 – 2.38 (m, 2H + 2H*), 2.05 (ddd, *J* = 12.6, 9.4, 5.4 Hz, 1H*), 1.91 (ddd, *J* = 12.6, 8.9, 2.5 Hz, 1H), 1.74 (dt, *J* = 12.8, 4.7 Hz, 1H), 1.60 (dt, *J* = 12.8, 2.8 Hz, 1H*). *The signals corresponding to the minor diastereomer are highlighted with an asterisk (*).*

Major diastereoisomer: **^{13}C NMR** (DMSO-d_6, 126 MHz, 80 °C) δ (ppm) 153.9, 137.3, 135.4, 128.8, 128.4, 128.3, 109.9, 98.5, 78.4, 66.1, 46.9, 40.7, 40.4.

Minor diastereoisomer: **^{13}C NMR** (DMSO-d_6, 126 MHz, 80 °C) δ (ppm) 153.9, 137.4, 135.1, 128.8, 128.3, 128.3, 110.3, 99.3, 79.5, 66.1, 47.1, 41.9, 40.0.

[xx] The substrate begins to precipitate out of solution at −78 °C if the addition of DIBAL-H is not performed immediately. However, in such case addition of DIBAL-H gradually re-dissolves the precipitated substrate.

$[\alpha]_D^{24}$: +24° (*c* 0.5, CH_2Cl_2).

HRMS: (ESI) calculated for $C_{15}H_{17}NO_4Na$ $(M+Na)^+$ requires: 298.1050, found: 298.1055.

IR: (film) ν_{max}/cm^{-1}: 3311, 3065, 2938, 1710, 1660, 1546, 1454, 1344, 1259, 1222, 1057, 1022, 830, 697.

(*R*, *Z*)-7-(4-Oxocyclopent-2-en-1-yl)hept-5-enoic acid (16)

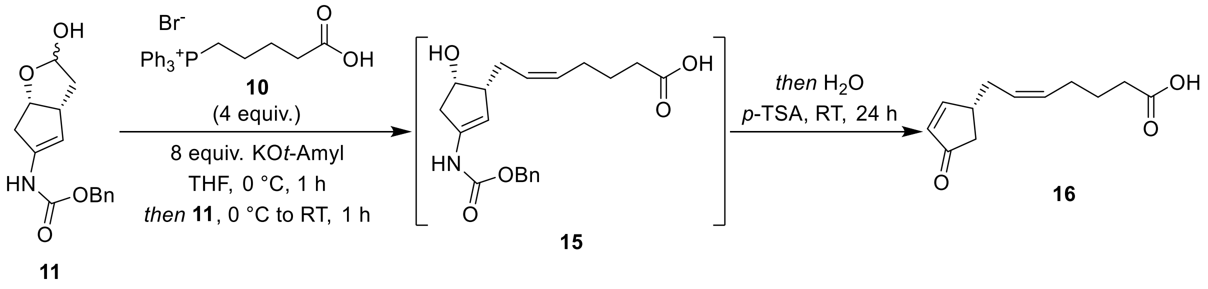

To phosphonium salt **10** (9.66 g, 21.8 mmol, 4.0 equiv.) under argon was added dry THF (50 mL). The resulting suspension was cooled to 0 °C. A potassium *tert*-amylate solution in cyclohexane (0.83 M, 52.5 mL, 43.6 mmol, 8.0 equiv.) was added dropwise (~2 drops/sec.) to the suspension, resulting in the appearance of a bright orange colour. After 2 h, all solids had dissolved, leaving behind a bright orange solution. Lactol **11** (1.50 g, 5.45 mmol, 1.0 equiv.) was dissolved in THF (20 mL) and the solution was degassed by sparging with argon for 30 min. During this time some THF evaporated to leave behind a ~17 mL solution. The lactol **11** solution was added to the orange ylid solution at 0 °C dropwise via syringe. To ensure complete substrate addition, further THF (5 mL) was added to the flask containing lactol **11**, and this solution was added to the reaction. The cooling bath was removed, and the mixture was stirred for 1 h. The mixture was again cooled to 0 °C and quenched with degassed water (50 mL, 3 × freeze thaw cycles) followed by addition of solid *p*-TSA·H_2O (16.6 g, 87.2 mmol, 16 equiv.). The resulting pale yellow two-layer mixture was stirred at 1200 rpm for 24 h at room temperature. The mixture was cooled to 0 °C and carefully basified with NaOH (2 M, aq.) to ~pH 12-13 and extracted with EtOAc (2 × 80 mL). The aqueous layer was acidified with HCl (3 M, aq.) to ~pH 3 and extracted with EtOAc (5 × 70 mL). The combined organic layers were washed with brine, dried ($MgSO_4$), filtered and concentrated *in vacuo* to give a pale yellow oily solid residue. The residue was triturated with 5% CH_2Cl_2/Ether mixture and filtered to remove most of the residual crystalline Wittig salt. The mother liquor was concentrated and purified on silica gel (hexanes/EtOAc, 40:60) to give acid **16** (0.89 g, 79%) as a colorless oil.

^{1}H NMR ($CDCl_3$, 400 MHz) δ (ppm) 7.62 (dd, *J* = 5.6, 2.5 Hz, 1H), 6.17 (dd, *J* = 5.6, 2.0 Hz, 1H), 5.54 – 5.32 (m, 2H), 3.06 – 2.93 (m, 1H), 2.52 (dd, *J* = 18.9, 6.3 Hz, 1H), 2.35 (t, *J* = 7.4 Hz, 2H), 2.32 – 2.14 (m, 2H), 2.12 – 2.05 (m, 2H), 2.01 (dd, *J* = 18.9, 2.2 Hz, 1H), 1.70 (p, *J* = 7.4 Hz, 2H).

^{13}**C NMR** ($CDCl_3$, 101 MHz) δ (ppm) 210.0, 179.2, 168.0, 134.1, 131.2, 126.8, 41.3, 40.4, 33.2, 31.8, 26.5, 24.4.

$[\alpha]_D^{24}$: +116° (*c* 0.95, $CHCl_3$)

HRMS: (ESI) calculated for $C_{12}H_{16}O_3Na$ $(M+Na)^+$ requires: 231.0997, found: 231.0989.

IR: (film) ν_{max}/cm^{-1}: 3009, 2925, 1704, 1666, 1583, 1405, 1350, 1310, 1183, 1086, 1039, 878.

tert-Butyl (_R_,_Z_)-7-(4-oxocyclopent-2-en-1-yl)hept-5-enoate (9)

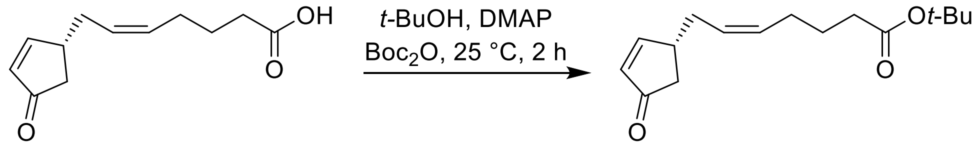

To a stirred solution of acid **16** (832 mg, 4.00 mmol, 1.0 equiv.) in *t*-BuOH (20 mL), was added 4-dimethylaminopyridine (147 mg, 1.20 mmol, 0.3 equiv.) and di-*tert*-butyl dicarbonate (1.74 g, 7.97 mmol, 2.0 equiv.) at room temperature. The reaction mixture was stirred at room temperature for 2 h. The solvent was evaporated under reduced pressure and the crude residue was purified by flash chromatography (hexanes/EtOAc, 85:15) to give *tert*-butyl ester **9** (876 mg, 83%) as a colorless oil.

R_f: 0.5 (hexanes/EtOAc, 70:30).

^{1}H NMR ($CDCl_3$, 400 MHz) δ (ppm) 7.62 (dd, *J* = 5.6, 2.4 Hz, 1H), 6.16 (dd, *J* = 5.6, 2.0 Hz, 1H), 5.54 – 5.44 (m, 1H), 5.44 – 5.32 (m, 1H), 3.06 – 2.93 (m, 1H), 2.51 (dd, *J* = 18.8, 6.4 Hz, 1H), 2.36 – 2.14 (m, 4H), 2.09 – 1.94 (m, 3H), 1.74 – 1.56 (m, 2H), 1.43 (s, 9H).

^{13}C NMR ($CDCl_3$, 101 MHz) δ (ppm) 209.7, 172.8, 167.8, 167.8, 134.1, 131.6, 126.4, 41.3, 40.4, 34.9, 31.8, 28.1, 26.6, 24.8.

$[\alpha]_D^{24}$: +110° (*c* 1.0, C_6H_6) [Lit. value: $[\alpha]_D^{25}$: +109° (*c* 1.0, C_6H_6)].[5]

Analytical data were consistent with those reported in the literature.[5]

(2*E*, 5*Z*)-Ethyl-octa-2,5-dienoate (20)

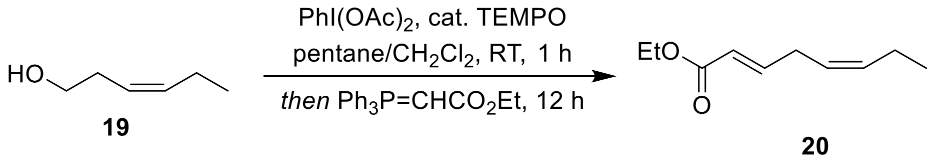

2,2,6,6-Tetramethyl-1-piperidinyloxy (TEMPO, 1.25 g, 8.00 mmol, 0.2 equiv.) was added to a stirred solution of (*Z*)-3-hexen-1-ol **19** (4.01 g, 40.0 mmol, 1.0 equiv.) and $PhI(OAc)_2$ (16.8 g, 52.0 mmol, 1.3 equiv.) in CH_2Cl_2:pentane (2:9, 80 mL) at 25 °C. The resulting reaction mixture was stirred for 1 h and ethyl(triphenylphosphoranylidene)acetate (20.9 g, 60.0 mmol, 1.5 equiv.) was added portionwise and the vigorous stirring was continued at the same temperature for 12 h. The volatiles were removed under reduced pressure and the crude reaction mixture was subjected to flash column chromatography (SiO_2, pentane:EtOAc, 97:3) to give dienoate **20** (4.24 g, 63%) as a colorless oil.

R_f: 0.8 (hexanes/EtOAc, 90:10).

^{1}H NMR ($CDCl_3$, 400 MHz) δ (ppm) 6.92 (dt, *J* = 15.6, 6.3 Hz, 1H), 5.80 (dt, *J* = 15.6, 1.8 Hz, 1H), 5.56 – 5.47 (m, 1H), 5.36 – 5.27 (m, 1H), 4.15 (q, *J* = 7.1 Hz, 2H), 2.91 (t, *J* = 8.1 Hz, 2H), 2.01 (p, *J* = 7.5 Hz, 2H), 1.25 (t, *J* = 7.1 Hz, 3H), 0.94 (t, *J* = 7.6 Hz, 3H).

^{13}C NMR ($CDCl_3$, 101 MHz) δ (ppm) 166.6, 147.2, 134.4, 123.4, 121.3, 60.1, 29.8, 20.4, 14.2, 14.0

Analytical data were consistent with those reported in the literature.[6]

Ethyl-(*S*,*Z*)-3-(4,4,5,5-tetramethyl-1,3,2-dioxaborolan-2-yl)oct-5-enoate (S2)

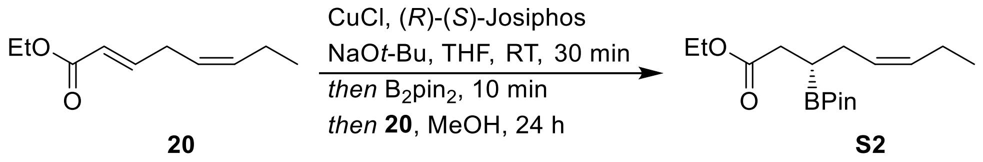

An oven-dried Schlenk flask was charged with CuCl (19.8 mg, 0.200 mmol, 0.02 equiv.), NaO*t*-Bu (28.8 mg, 0.300 mmol, 0.03 equiv.), and (*R*)-(*S*)-Josiphos ligand (256 mg, 0.375 mmol, 0.04 equiv.) and the solids were dissolved in THF (9.0 mL) under a nitrogen atmosphere at 25 °C and stirred for 30 min. Bis(pinacolato)diboron (2.79 g, 11.0 mmol, 1.1 equiv.) and THF (6.0 mL) were added and the stirring was continued for 10 min. *α,β*-Unsaturated ester **20** (1.68 g, 10.0 mmol, 1.0 equiv.) and then MeOH (1.0 mmol, 0.04 mL) were added. The Schlenk flask was washed with THF (4.0 mL), and stirred for 24 h. The reaction mixture was passed through a small pad of celite and concentrated. The crude reaction mixture was purified by flash column chromatography (SiO_2, pentane:EtOAc, 97:3) to give boronic ester **S2** (2.52 g, 85%, 93:7 er) as a colourless oil.

[The racemate (±)-**S2** was prepared using DPEPhos in place of (*R*)-(*S*)-Josiphos]

R*f*: 0.55, EtOAc:Hexanes (10:90).

^{1}H NMR ($CDCl_3$, 400 MHz) δ (ppm) 5.44 – 5.32 (m, 1H), 5.32 – 5.19 (m, 1H), 4.14 – 4.01 (m, 2H), 2.46 – 2.30 (m, 2H), 2.22 – 2.06 (m, 2H), 2.06 – 1.92 (m, 2H), 1.42 – 1.30 (m, 1H), 1.25 – 1.18 (m, 15H), 0.91 (t, *J* = 7.5 Hz, 3H).

^{13}C NMR ($CDCl_3$, 101 MHz) δ (ppm) 174.0, 132.8, 127.9, 83.3, 60.2, 35.0, 27.6, 24.9, 24.7, 20.5, 14.3, 14.3.

$[\alpha]_D^{24}$: −7° (*c* 1.0, $CHCl_3$).

HRMS: (ESI) calculated for $C_{16}H_{29}BO_4Na$ $(M+Na)^+$ requires: 319.2051, found: 319.2051.

IR: (film) ν_{max}/cm^{-1}: 2976, 2933, 1731, 1445, 1417, 1370, 1317, 1140, 1028, 966.

*The ee was determined following oxidation to alcohol **S3** and subsequent benzoylation to benzoate **S4**.*

(*S*, *Z*)-1-Ethoxy-1-oxooct-5-en-3-yl benzoate (S3)

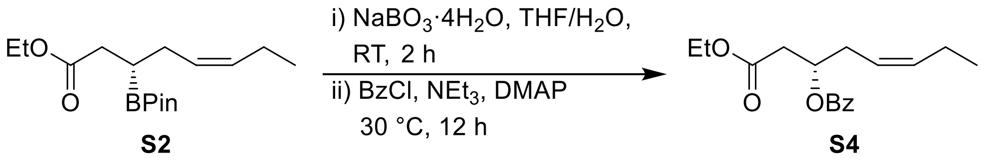

(i). **Oxidation**: To a stirred solution of secondary *β*-boronyl-ethyl ester **S2** (29.6 mg, 0.10 mmol, 1.0 equiv.) in THF:H_2O (1:1, 2.0 mL) was added a $NaBO_3 \cdot 4H_2O$ (77 mg, 0.50 mmol, 5.0 equiv.) in one portion and the resulting mixture was vigorously stirred for 2 h at 25 °C under open air. The reaction mixture was diluted with H_2O (3 mL) and extracted with ethyl acetate (3 × 5 mL). The combined organic phases were dried over anhydrous Na_2SO_4 and concentrated *in vacuo*. The crude residue was purified by flash column chromatography (R_f: 0.2, EtOAc:Hexanes (10:90); SiO_2, pentane:EtOAc, 92:8) to give the desired hydroxy compound **S3** (17.1 mg, 92%) as a colourless liquid. The hydroxy compound was utilized directly in the next step (benzoylation) without characterization.

(ii). ***O*-Benzoylation**: The hydroxy compound **S3** was dissolved in anhydrous dichloromethane (2 mL) and cooled to 0 °C. Triethylamine (28 mg, 0.276 mmol, 3.0 equiv.) and *N*,*N*-dimethylaminopyridine (DMAP, 0.1 mg, 1.0 mol %) were added, and benzoyl chloride (19 mg, 0.14 mmol, 1.5 equiv.) was slowly added at 0 °C. The reaction mixture was allowed to react at 30 °C. After 12 h, the reaction was quenched by adding water (1.0 mL) and the reaction mixture was extracted with CH_2Cl_2 (3 × 3 mL). The combined organic layers were dried over Na_2SO_4, evaporated under reduced pressure, and purified by flash column chromatography on silica gel (pentane:EtOAc, 95:5) to give benzoate **S4** (25 mg, 95%, 86% ee) as a colourless oil.

[The racemate was prepared using (±)-**S2** in place of (−)-**S4**]

R_f: 0.5 (EtOAc:Hexanes 10:90).

^{1}H NMR ($CDCl_3$, 400 MHz) δ (ppm) 8.02 (d, *J* = 6.9 Hz, 2H), 7.54 (t, *J* = 7.5 Hz, 1H), 7.43 (t, *J* = 7.1 Hz, 2H), 5.60 – 5.44 (m, 2H), 5.44 – 5.32 (m, 1H), 4.11 (q, *J* = 7.1 Hz, 2H), 2.81 – 2.63 (m, 2H), 2.62 – 2.42 (m, 2H), 2.07 (p, *J* = 7.2 Hz, 2H), 1.19 (t, *J* = 7.1 Hz, 3H), 0.95 (t, *J* = 7.5 Hz, 3H).

^{13}C NMR ($CDCl_3$, 101 MHz) δ (ppm) 170.3, 165.7, 135.5, 132.9, 130.2, 129.6, 128.3, 122.4, 70.9, 60.6, 38.7, 31.6, 20.6, 14.1, 14.1.

$[\alpha]_D^{24}$: −17.2° (*c* 1.0, $CHCl_3$).

HRMS: (ESI) calculated for $C_{16}H_{29}BO_4Na$ $(M+Na)^+$ requires: 313.1410, found: 313.1414.

IR: (film) ν_{max}/cm^{-1}: 2964, 2874, 1717, 1602, 1584, 1492, 1451, 1369, 1314, 1269, 1174, 1108, 1025, 937.

Chiral HPLC Conditions: Chiralpak® IC Column, Hexane/IPA: 97/3, flow rate: 1.0 mL/min; t_R (major) = 9.1 min, t_R (minor) = 14.6 min; er = 93.4:6.6.

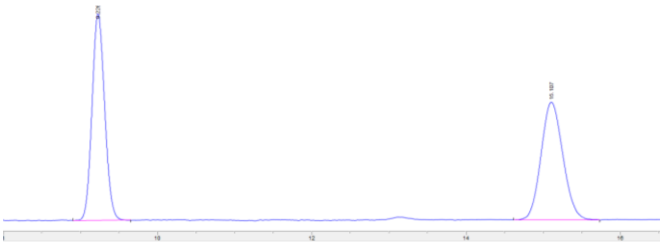

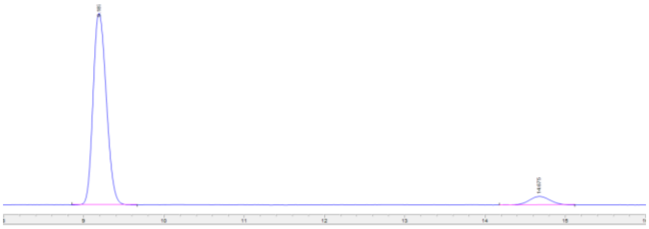

#	Time	Type	Area	Height	Width	Area%	Symmetry
1	9.187	BV R	13042.7	1134.2	0.1387	93.385	0.807
2	14.675	VV R	923.9	50.7	0.2158	6.615	0.931

(*Z*)-3-(4,4,5,5-Tetramethyl-1,3,2-dioxaborolan-2-yl)oct-5-enal (8)

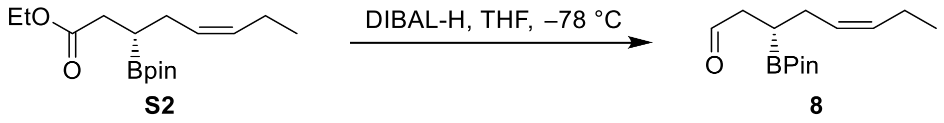

To a solution of ethyl ester **S2** (1.50 g, 5.06 mmol, 1.0 equiv.) in CH_2Cl_2 (111 mL) at –78 °C was added DIBAL-H (1.0 M solution in hexanes, 6.59 mL of a 6.59 mmol, 1.3 equiv.) dropwise over 20 min. The reaction was stirred for 3 h at the same temperature, MeOH (28 mL) was added, followed by saturated aqueous sodium potassium tartarate (28 mL). The organic phase was separated, and the aqueous layer was extracted with CH_2Cl_2 (3 × 30 mL). The combined organic layers were dried over anhydrous Na_2SO_4 and concentrated *in vacuo*. The resulting crude residue was purified by flash column chromatography (SiO_2, pentane:EtOAc, 96:4) to give aldehyde **8** (1.19 g, 93%) as a colorless liquid.

R*f*: 0.5 (EtOAc:Hexanes 10:90).

^{1}H NMR ($CDCl_3$, 400 MHz) δ (ppm) 9.74 (S, 1H), 5.46 – 5.31 (m, 1H), 5.31 – 5.15 (m, 1H), 2.64 – 2.44 (m, 2H), 2.24 – 2.05 (m, 2H), 1.99 (p, *J* = 7.5, 1.5 Hz, 2H), 1.45 – 1.30 (m, 1H), 1.22 (d, *J* = 5.6 Hz, 10H), 0.92 (t, *J* = 7.5 Hz, 3H).

^{13}C NMR ($CDCl_3$, 101 MHz) δ (ppm) 202.7, 132.9, 127.7, 83.3, 45.1, 27.4, 24.7, 24.6, 20.5, 14.2.

$[\alpha]_D^{24}$: −16° (*c* 1.0, $CHCl_3$).

HRMS: (ESI) calculated for $C_{14}H_{25}BO_3Na$ $(M+Na)^+$ requires: 275.1789, found: 275.1798.

IR: (film) ν_{max}/cm^{-1}: 2975, 2932, 1720, 1455, 1379, 1320, 1215, 1142, 1008, 967.

***tert*-Butyl (*Z*)-7-((*S*,*E*)-5-((*S*,*Z*)-3-hydroxyoct-5-en-1-ylidene)-4-oxocyclopent-2-en-1-yl)hept-5-enoate (18)**

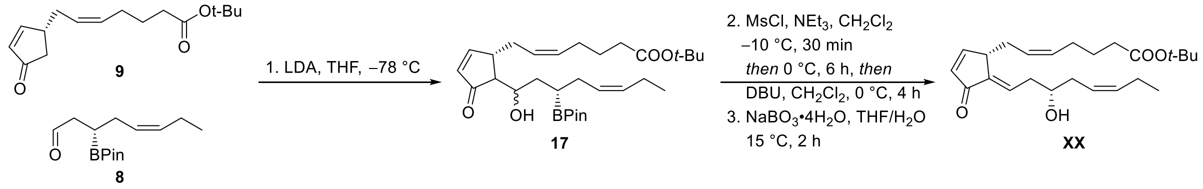

(i). **Aldol reaction**: To a premixed solution of enone **9** (132 mg, 0.499 mmol, 1.0 equiv.) and freshly prepared aldehyde **8** (176 mg, 0.698 mmol, 1.4 equiv.) in THF (21 mL) at −78 °C was added LDA (0.218 M in THF, 4.57 mL, 1.00 mmol, 2.0 equiv.) dropwise over a period of 45 min under an argon atmosphere. After stirring for 20 min at −78 °C, the light-yellow reaction mixture was quenched with a saturated aqueous NH_4Cl solution (10 mL), diluted with EtOAc (15 mL), and allowed to warm to 25 °C. The two phases were separated, and the aqueous layer was extracted with EtOAc (3 × 25 mL). The combined organic layers were washed with brine (15 mL), dried over $MgSO_4$, filtered, and concentrated under reduced pressure. The crude aldol product **17** was directly taken into the next step without further purification.

(ii). **Mesylation**: To a stirred solution of the crude aldol product **17** in CH_2Cl_2 (10 mL) at −10 °C was added Et_3N (303 mg, 2.99 mmol, 6.0 equiv.), and then, slowly and dropwise, methanesulfonyl chloride (172 mg, 1.50 mmol, 3.0 equiv.) under an argon atmosphere. After stirring for 30 min at this temperature, the reaction mixture warmed up to 0 °C and stirred for 6 h. The reaction mixture was quenched with brine (15 mL) and diluted with CH_2Cl_2 (15 mL). The phases were separated, the aqueous layer was extracted with CH_2Cl_2 (3 × 15 mL), and the combined organic layers were washed with saturated aqueous $NaHCO_3$ solution (3 mL) and H_2O (3 mL), dried ($MgSO_4$), filtered, and concentrated under reduced pressure. The crude mesylated product was directly taken into the next step without further purification.

(iii). **Elimination**: 1,8-Diazabicyclo[5.4.0]undec-7-ene (DBU, 457 mg, 3.00 mmol, 6.0 equiv.) was added to a solution of the crude mesylated product in CH_2Cl_2 (10 mL) in a round bottom flask at −10 °C under an argon atmosphere and stirred at 0 °C. After 4 h, the reaction was quenched by adding brine (3 mL) and the reaction mixture was extracted with dichloromethane (3 × 3 mL). The combined organic layers were dried over $MgSO_4$, evaporated under reduced pressure. The crude eliminated product was directly taken into the next step without further purification.

(iv). **Bpin-oxidation**: To a stirred solution of the crude eliminated product in THF:H_2O (1:1, 5.0 mL) was added a $NaBO_3·4H_2O$ (385 mg, 2.50 mmol, 5.0 equiv.) in one portion at 10 °C and the resulting mixture was vigorously stirred for 2 h at 15 °C (open to air). The reaction mixture was diluted with H_2O (5 mL) and extracted with EtOAc (3 × 5 mL). The combined organic phases were dried over anhydrous $MgSO_4$ and concentrated *in vacuo* to a volume of *ca.* 2 mL (not to dryness!). The crude residue was purified by flash column chromatography (76:24; hexane/EtOAc) to give the desired hydroxy compound **18** (89.2 mg, 23%) as a colourless liquid.

R_f: 0.2 (EtOAc:Hexanes 30:70).

^{1}H NMR ($CDCl_3$, 400 MHz) δ (ppm) 7.50 (d, *J* = 6.0 Hz, 1H), 6.65 (t, *J* = 7.8 Hz, 1H), 6.34 (d, *J* = 5.8 Hz, 1H), 5.65 – 5.54 (m, 1H), 5.53 – 5.42 (m, 1H), 5.42 – 5.28 (m, 2H), 3.89 – 3.76 (m, 1H), 3.59 – 3.47 (m, 1H), 2.65 – 2.54 (m, 1H), 2.47 (q, *J* = 7.6 Hz, 2H), 2.35 – 2.14 (m, 5H), 2.12 – 1.95 (m, 4H), 1.72 – 1.53 (m, 3H), 1.43 (s, 9H), 0.97 (t, *J* = 7.4 Hz, 3H).

^{13}C NMR ($CDCl_3$, 101 MHz) δ (ppm) 196.3, 172.9, 161.6, 139.3, 135.7, 134.9, 131.7, 131.5, 125.6, 123.7, 80.2, 70.5, 43.3, 36.5, 34.9, 34.8, 30.2, 28.1, 26.7, 24.8, 20.7, 14.2.

$[\alpha]_D^{24}$: 47° (*c* 0.5, CH_2Cl_2).

HRMS: (ESI) calculated for $C_{24}H_{36}O_4Na$ $(M+Na)^+$ requires: 411.2506, found: 411.2515.

IR: (film) ν_{max}/cm^{-1}: 3441, 2924, 2850, 1727, 1703, 1652, 1579, 1457, 1366, 1255, 1147, 967.

(*Z*)-7-((*S*,*E*)-5-((*S*,*Z*)-3-hydroxyoct-5-en-1-ylidene)-4-oxocyclopent-2-en-1-yl)hept-5-enoic acid (3)

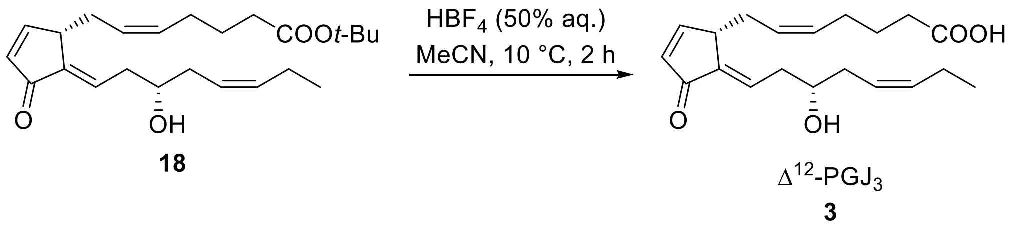

A solution of HBF_4 (50% aq., 0.386 mL, 2.20 mmol, 22 equiv.) was added dropwise to a stirred solution of *tert*-butyl ester **18** (38.8 mg, 0.100 mmol, 1.0 equiv.) in acetonitrile (4.6 mL) at −10 °C. The reaction mixture was stirred for 2 h at 10 °C, and then quenched with brine (3 mL) and extracted with EtOAc (4 × 10 mL). The combined organic layers were dried ($MgSO_4$), and concentrated to a volume of *ca.* 2 mL (not to dryness!). Flash column chromatography (SiO_2, CH_2Cl_2:EtOH, 96:4) yielded the title compound, Δ^{12}-PGJ_3 **3** (24.9 mg, 75%), as a colourless oil.

R_f: 0.6 (CH_2Cl_2:EtOH 90:10).

^{1}H NMR ($CDCl_3$, 500 MHz) δ (ppm) 7.58 (dd, *J* = 6.1, 2.5 Hz, 1H), 6.60 (t, *J* = 7.7 Hz, 1H), 6.36 (dd, *J* = 6.1, 1.8 Hz, 1H), 5.66 – 5.57 (m, 1H), 5.55 – 5.42 (m, 2H), 5.42 – 5.31 (m, 1H), 3.87 (quint, *J* = 6.0 Hz, 1H), 3.52 (ddd, *J* = 9.1, 4.5, 2.3 Hz, 1H), 2.77 – 2.68 (m, 1H), 2.62 – 2.47 (m, 2H), 2.45 – 2.26 (m, 4H), 2.25 – 2.14 (m, 1H), 2.14 – 1.98 (m, 5H), 1.83 – 1.56 (m, 2H), 0.97 (t, *J* = 7.5 Hz, 3H).

^{13}C NMR ($CDCl_3$, 126 MHz) δ (ppm) 196.3, 175.9, 161.7, 139.8, 136.1, 134.8, 131.6, 131.5, 126.1, 123.6, 71.1, 43.8, 36.2, 34.6, 32.7, 30.5, 26.4, 24.5, 20.7, 14.2.

$[\alpha]_D^{24}$: + 113° (*c* 0.46, C_6H_6).

HRMS: (ESI) calculated for $C_{20}H_{28}O_4Na$ $(M+Na)^+$ requires: 355.1880, found: 355.1872.

IR: (film) ν_{max}/cm^{-1}: 2981, 2963, 1737, 1711, 1373, 1237, 1095, 1044, 938.

Analytical data were consistent with those reported in the literature.[5]

5. NMR Spectra

13

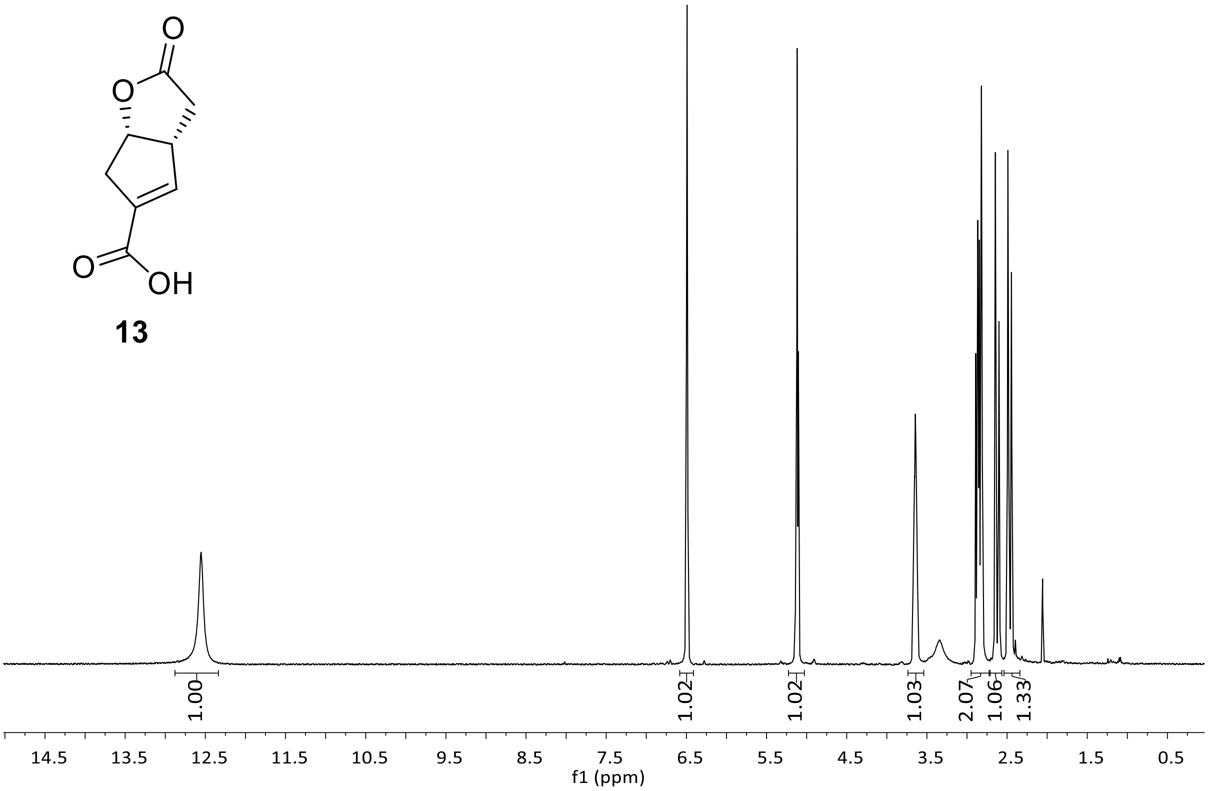

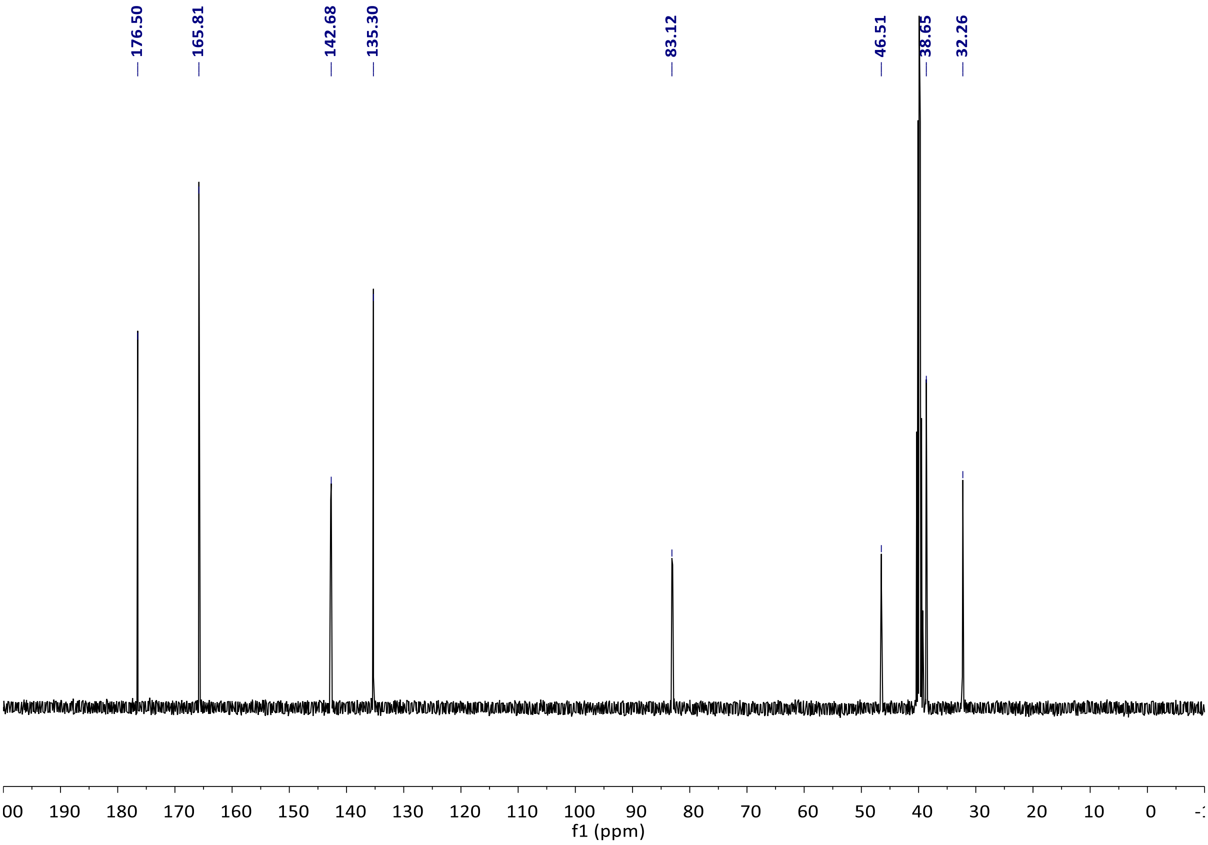

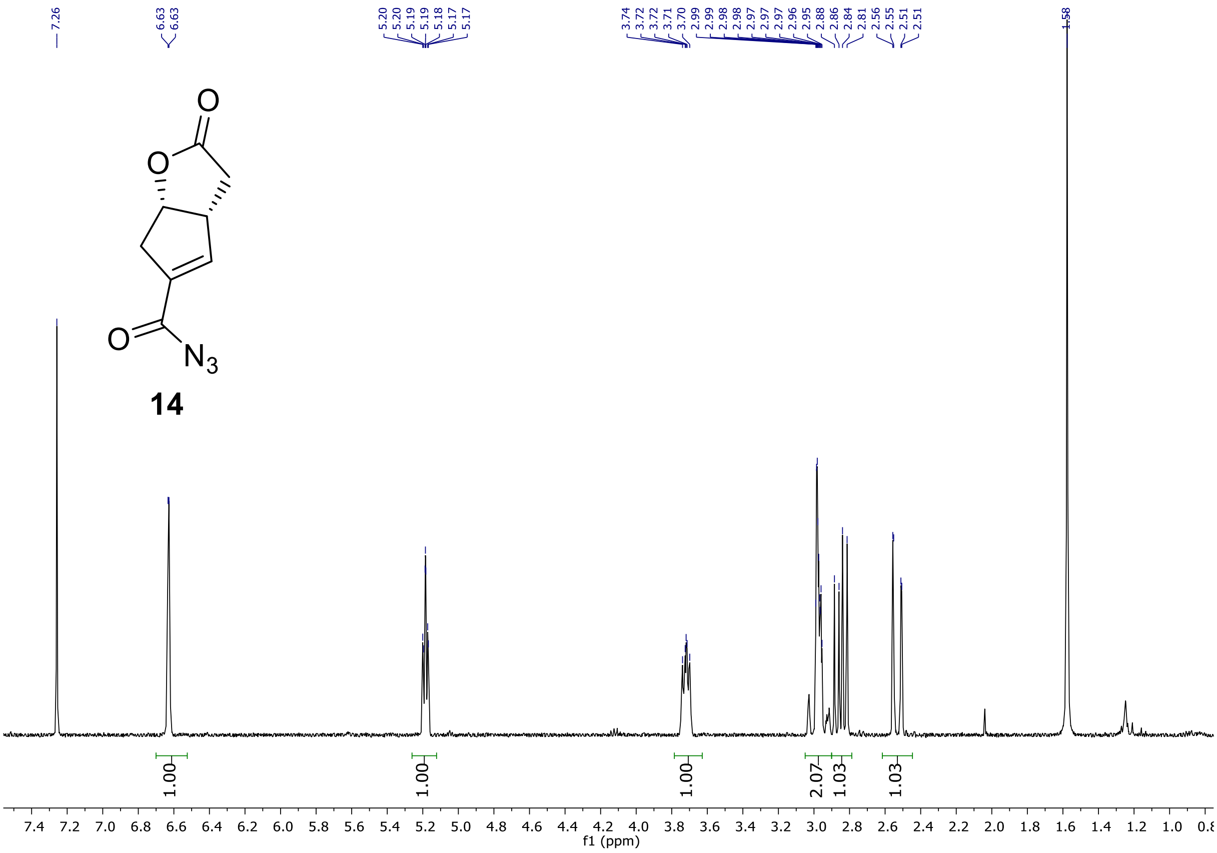

14
7.26
6.63
6.63
5.20
5.20
5.19
5.19
5.18
5.17
5.17
3.74
3.72
3.72
3.71
3.70
2.99
2.99
2.98
2.98
2.97
2.97
2.97
2.96
2.95
2.88
2.86
2.84
2.81
2.56
2.55
2.51
2.51
1.58
1.00
1.00
1.00
2.07
1.03
1.03
7.4 7.2 7.0 6.8 6.6 6.4 6.2 6.0 5.8 5.6 5.4 5.2 5.0 4.8 4.6 4.4 4.2 4.0 3.8 3.6 3.4 3.2 3.0 2.8 2.6 2.4 2.2 2.0 1.8 1.6 1.4 1.2 1.0
f1 (ppm)

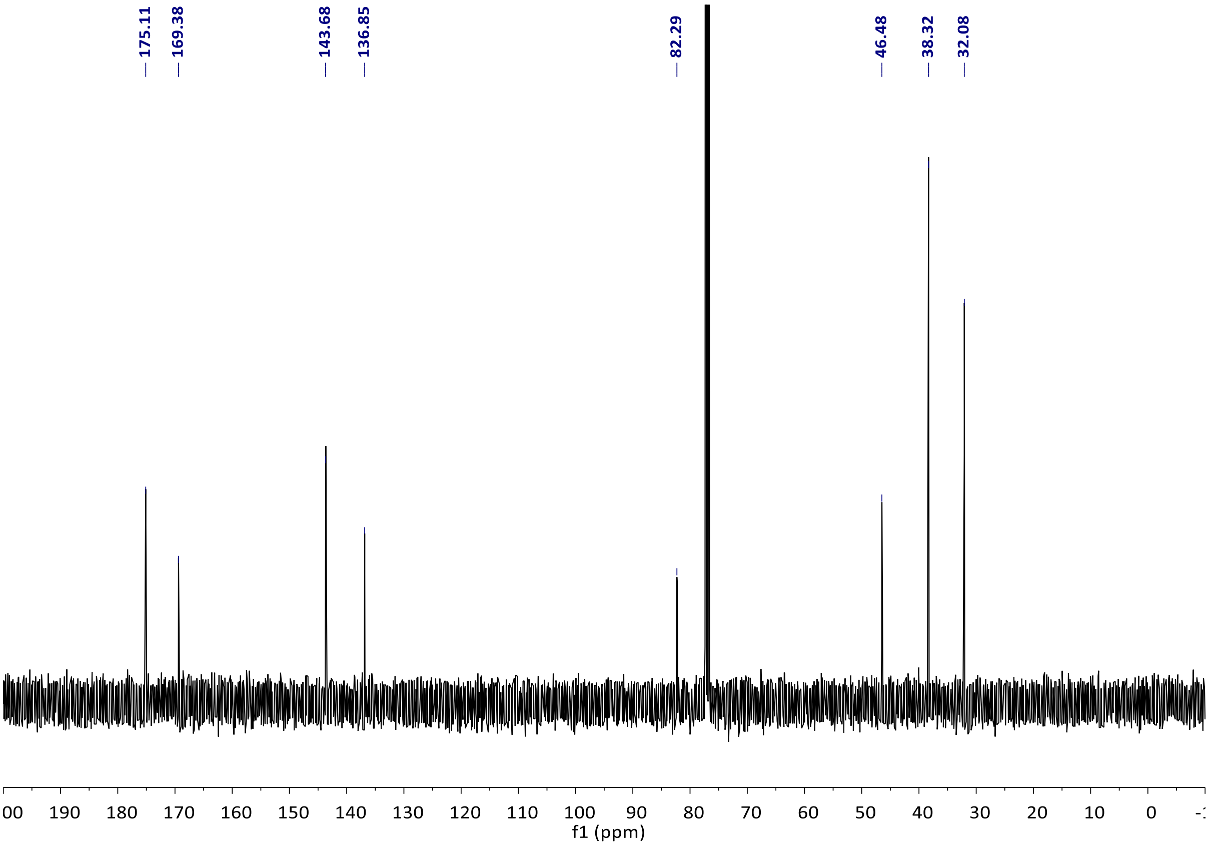

175.11
169.38
143.68
136.85
82.29
46.48
38.32
32.08
190 180 170 160 150 140 130 120 110 100 90 80 70 60 50 40 30 20 10 0
f1 (ppm)

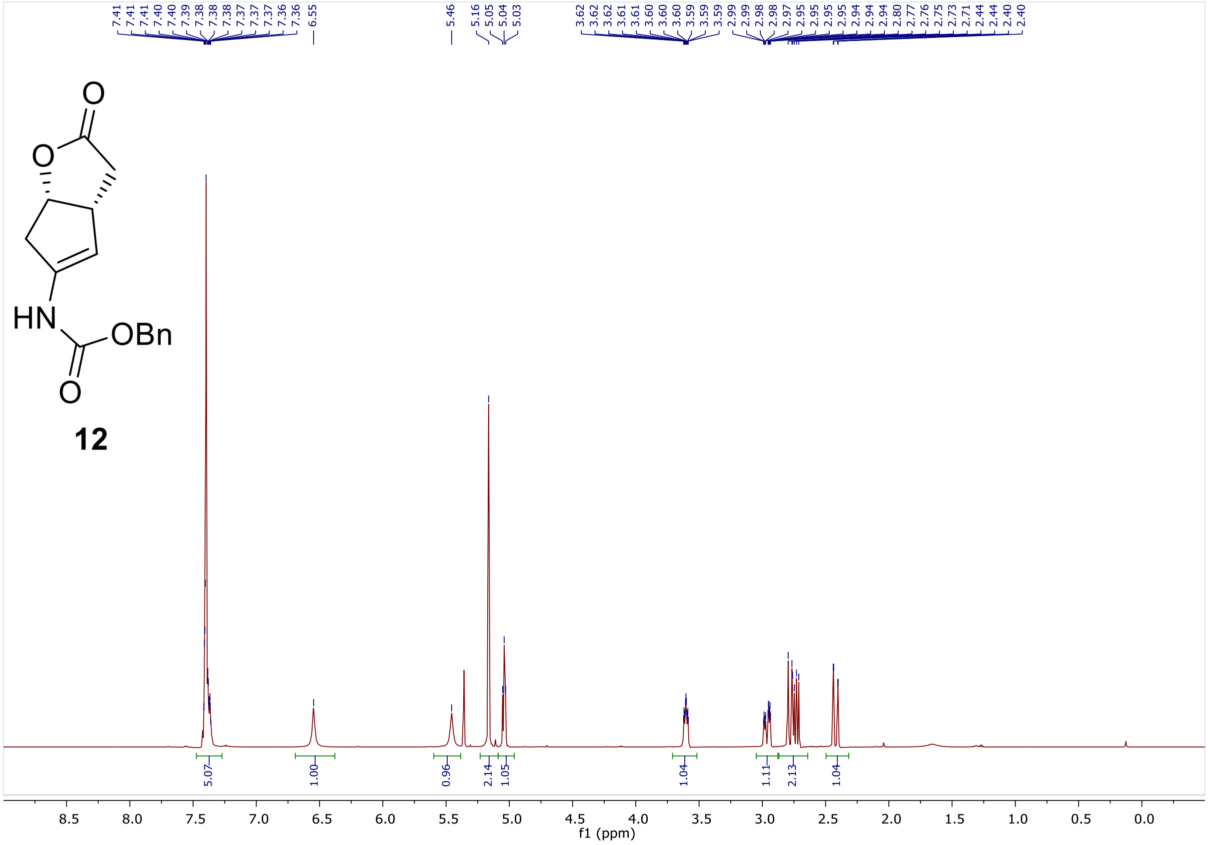
7.41 7.41 7.41 7.40 7.39 7.38 7.38 7.38 7.37 7.37 7.37 7.36 7.36 6.55
5.46 5.16 5.05 5.04 5.03
3.62 3.62 3.62 3.61 3.61 3.60 3.60 3.60 3.59 3.59 3.59 2.99 2.99 2.98 2.98 2.97 2.95 2.95 2.95 2.95 2.94 2.94 2.94 2.94 2.80 2.77 2.76 2.75 2.73 2.71 2.44 2.40 2.40
O
O
HN
OBn
O
12
5.07 1.00 0.96 2.14 1.05 1.04 1.11 2.13 1.04
8.5 8.0 7.5 7.0 6.5 6.0 5.5 5.0 4.5 4.0 3.5 3.0 2.5 2.0 1.5 1.0 0.5 0.0
f1 (ppm)

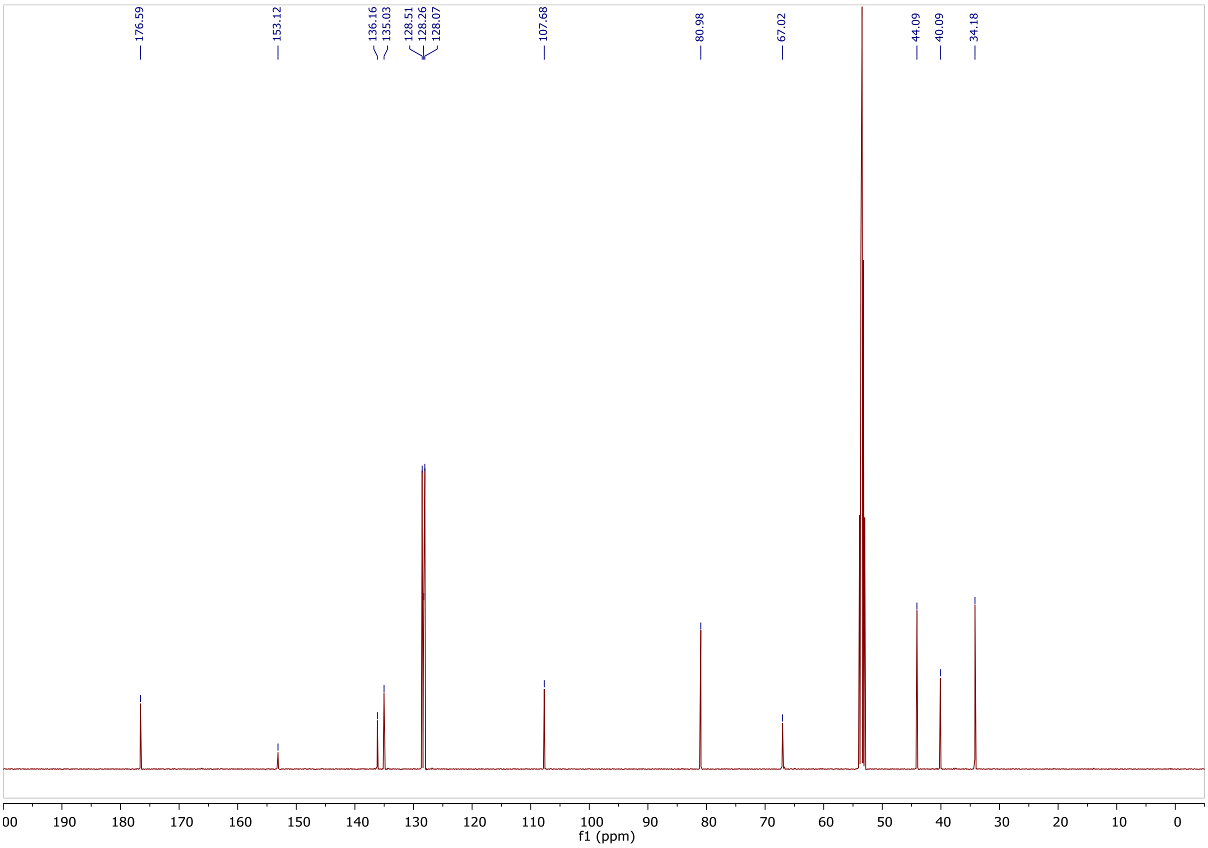
176.59
153.12
136.16 135.03
128.51 128.26 128.07
107.68
80.98
67.02
44.09 40.09 34.18
00 190 180 170 160 150 140 130 120 110 100 90 80 70 60 50 40 30 20 10 0
f1 (ppm)

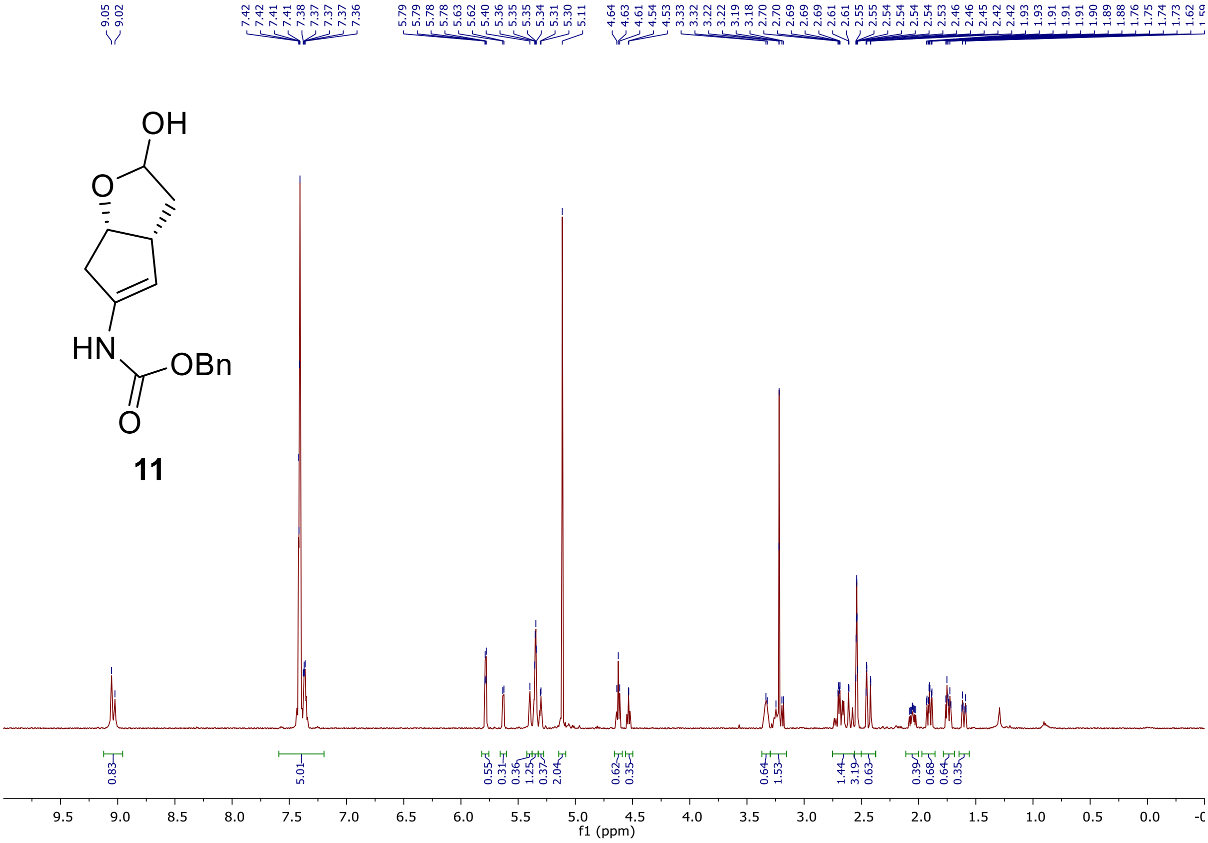
9.05
9.02
7.42
7.42
7.41
7.38
7.37
7.37
7.37
7.36
5.79
5.79
5.78
5.78
5.63
5.62
5.40
5.36
5.35
5.35
5.34
5.31
5.30
5.11
4.64
4.63
4.61
4.54
4.53
3.33
3.32
3.22
3.22
3.19
3.18
2.70
2.70
2.69
2.69
2.69
2.61
2.61
2.55
2.55
2.54
2.54
2.54
2.54
2.53
2.46
2.46
2.45
2.42
2.42
1.93
1.93
1.91
1.91
1.91
1.90
1.89
1.88
1.76
1.74
1.73
1.62
OH
O
HN
OBn
O
11
0.83
5.01
0.55
0.31
0.36
1.25
0.37
2.04
0.62
0.35
0.64
1.53
1.44
3.19
0.63
0.39
0.68
0.64
0.35
9.5 9.0 8.5 8.0 7.5 7.0 6.5 6.0 5.5 5.0 4.5 4.0 3.5 3.0 2.5 2.0 1.5 1.0 0.5 0.0
f1 (ppm)

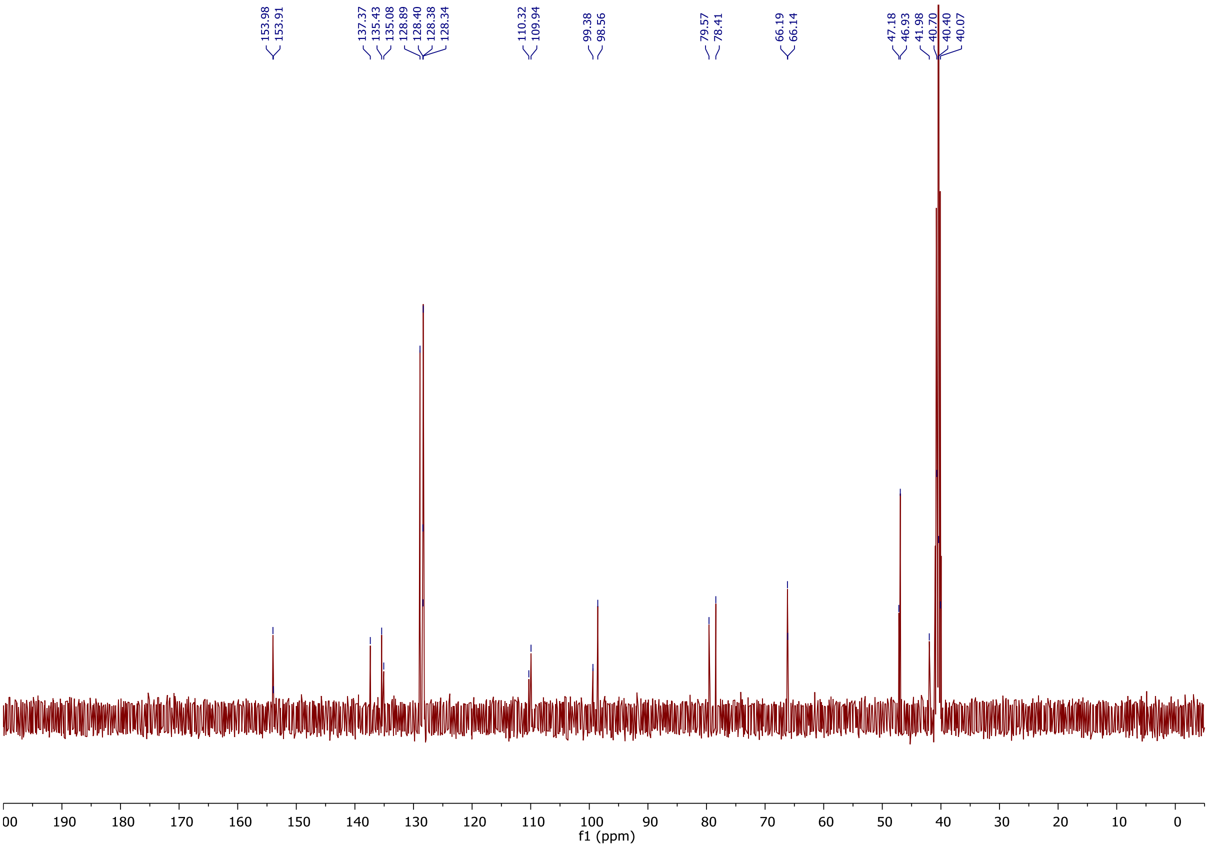
153.98
153.91
137.37
135.43
135.08
128.89
128.40
128.38
128.34
110.32
109.94
99.38
98.56
79.57
78.41
66.19
66.14
47.18
46.93
41.98
40.70
40.40
40.07
190 180 170 160 150 140 130 120 110 100 90 80 70 60 50 40 30 20 10 0
f1 (ppm)

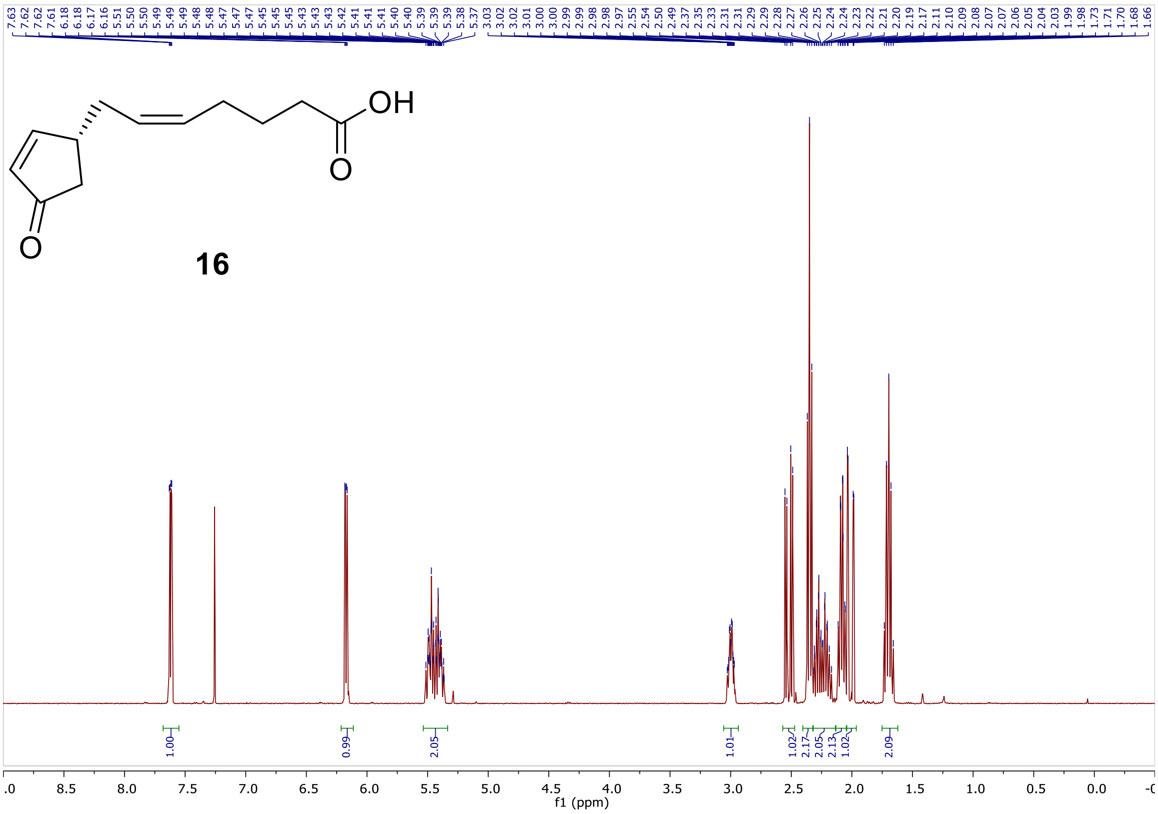
OH
O
O
16
1.00
0.99
2.05
1.01
1.02
2.17
2.05
2.13
1.02
2.09
f1 (ppm)

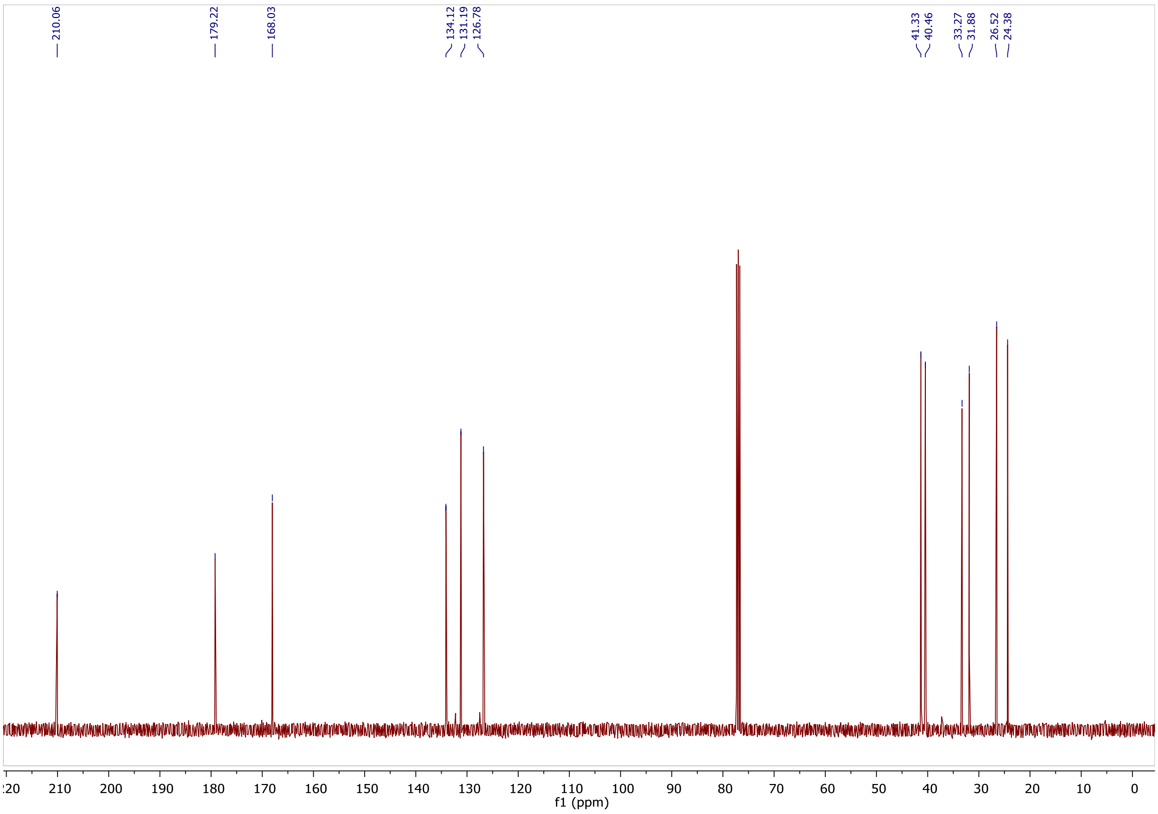
210.06
179.22
168.03
134.12
131.19
126.78
41.33
40.46
33.27
31.88
26.52
24.38
f1 (ppm)

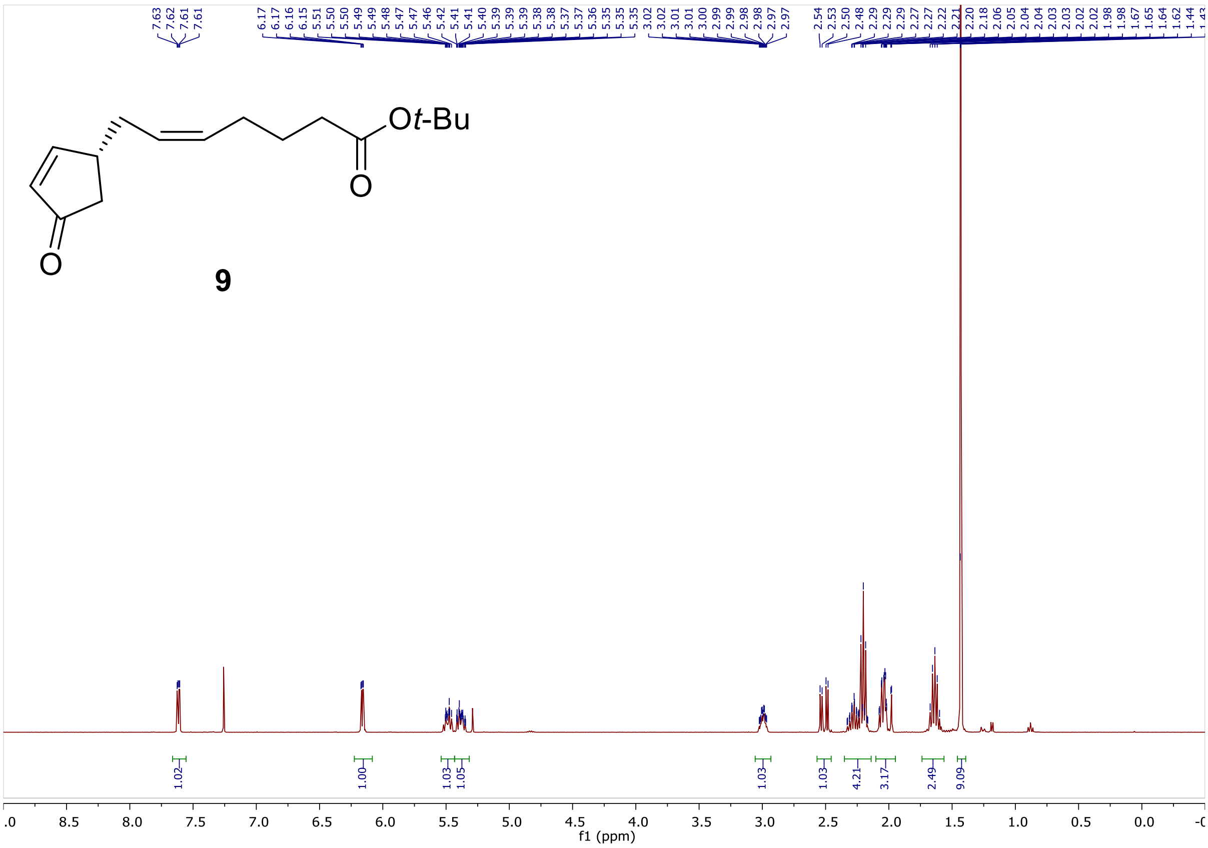

7.63
7.62
7.61
7.61
6.17
6.17
6.16
6.15
5.51
5.50
5.50
5.49
5.48
5.47
5.47
5.46
5.42
5.41
5.41
5.40
5.39
5.39
5.39
5.38
5.37
5.37
5.36
5.35
5.35
5.35
3.02
3.02
3.01
3.01
3.00
2.99
2.99
2.98
2.98
2.97
2.97
2.54
2.53
2.50
2.48
2.29
2.29
2.29
2.27
2.27
2.22
2.21
2.20
2.18
2.06
2.05
2.04
2.04
2.03
2.02
1.98
1.98
1.67
1.65
1.64
1.62
1.44
1.43[illegible]
Ot-Bu
O
O
9
1.02
1.00
1.03
1.05
1.03
1.03
4.21
3.17
2.49
9.09
f1 (ppm)

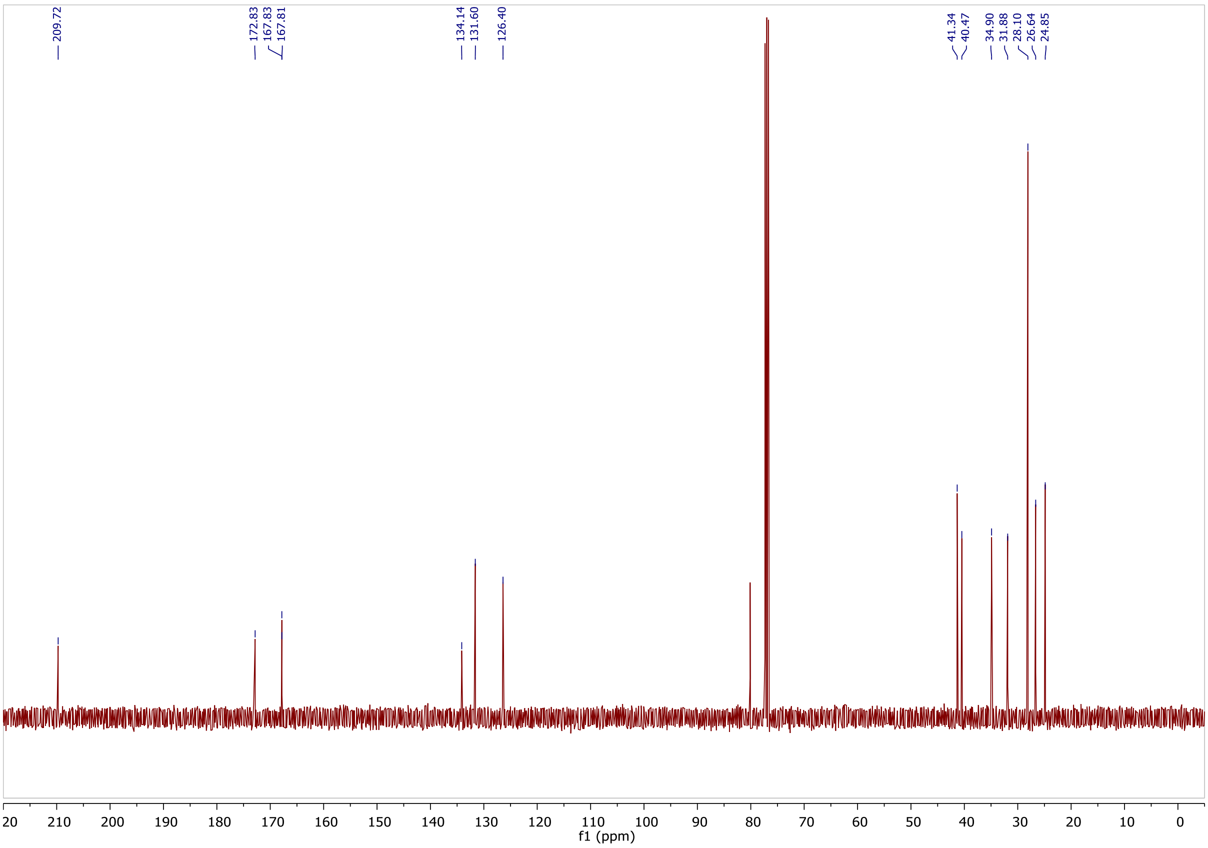

209.72
172.83
167.83
167.81
134.14
131.60
126.40
41.34
40.47
34.90
31.88
28.10
26.64
24.85
f1 (ppm)

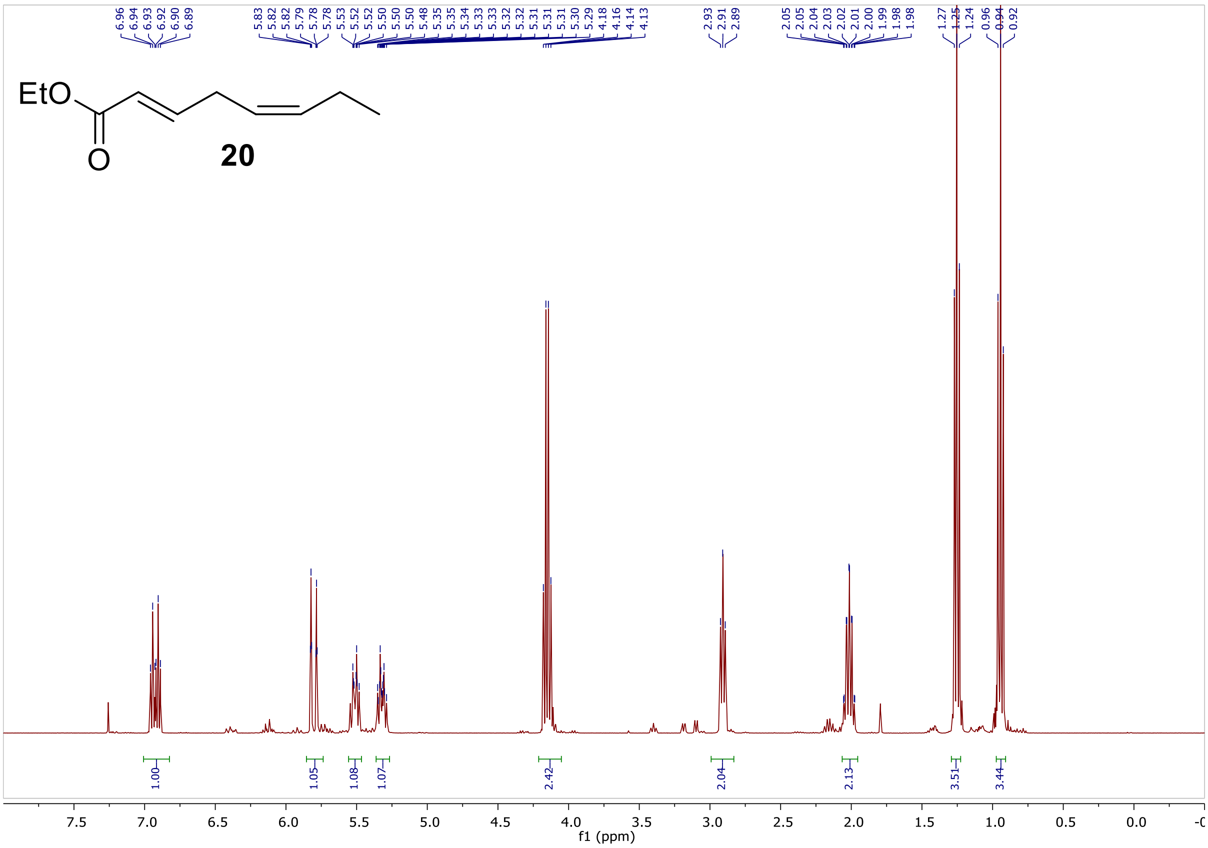
EtO
O
20
f1 (ppm)

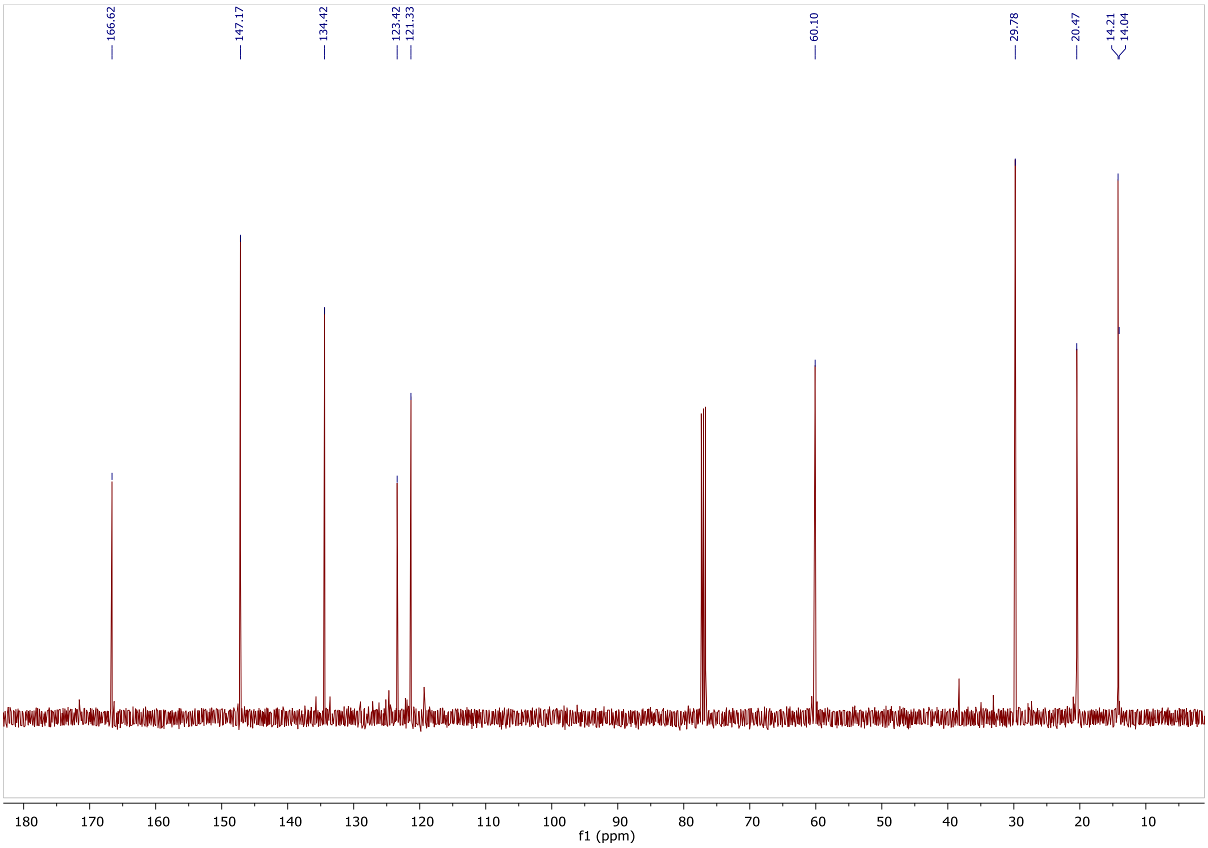
166.62
147.17
134.42
123.42
121.33
60.10
29.78
20.47
14.21
14.04
f1 (ppm)

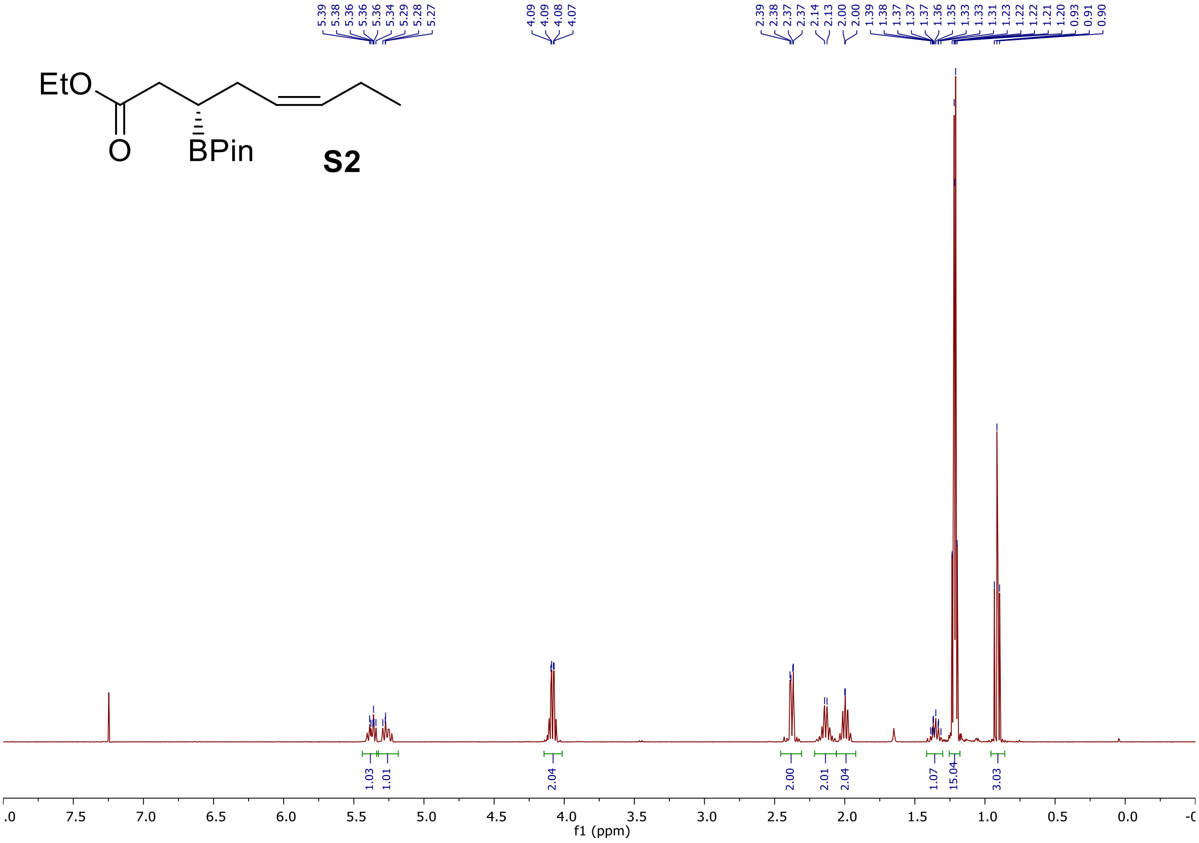

S2

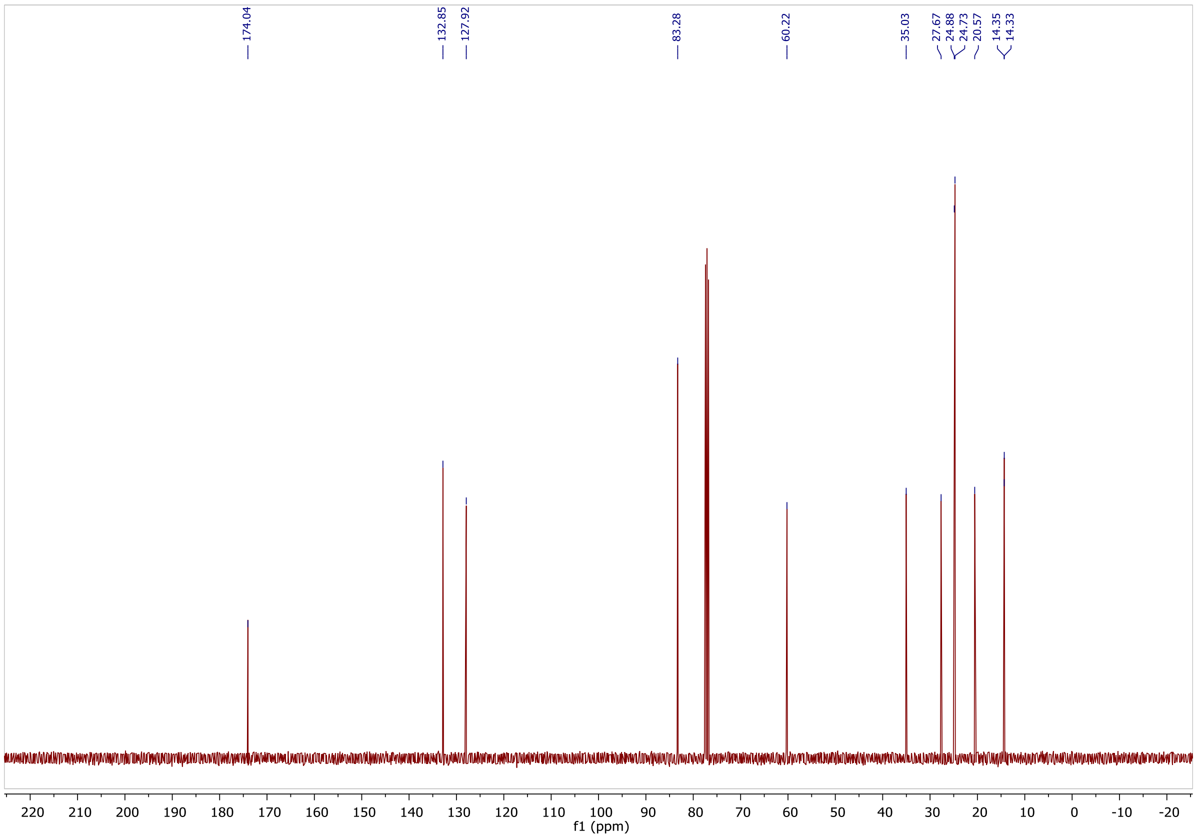

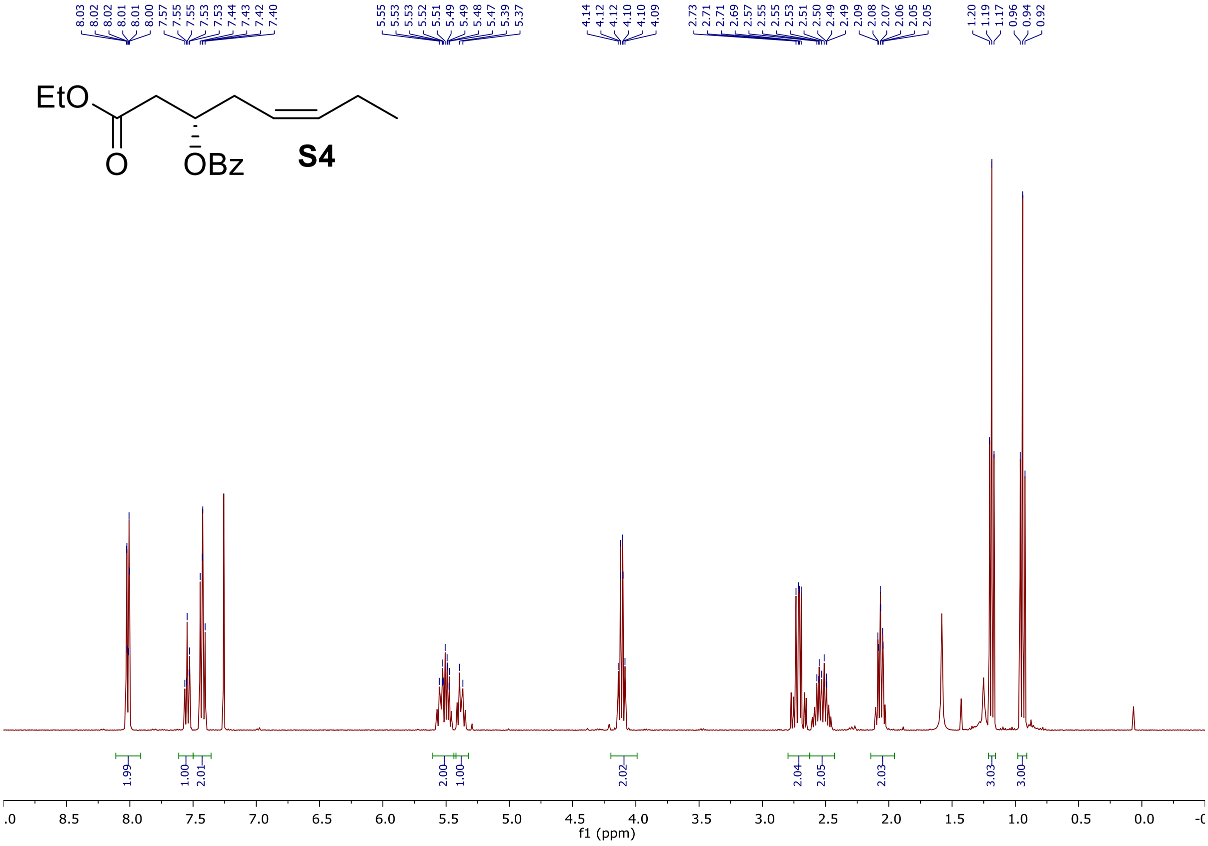
8.03 8.02 8.01 8.01 8.00 7.57 7.55 7.55 7.53 7.53 7.44 7.43 7.42 7.40
5.55 5.53 5.53 5.52 5.51 5.49 5.49 5.48 5.47 5.39 5.37
4.14 4.12 4.10 4.10 4.09
2.73 2.71 2.71 2.69 2.57 2.55 2.55 2.53 2.51 2.50 2.49 2.49 2.09 2.08 2.07 2.06 2.05 2.05
1.20 1.19 1.17 0.96 0.94 0.92
EtO
O
OBz
S4
1.99
1.00
2.01
2.00
1.00
2.02
2.04
2.05
2.03
3.03
3.00
f1 (ppm)

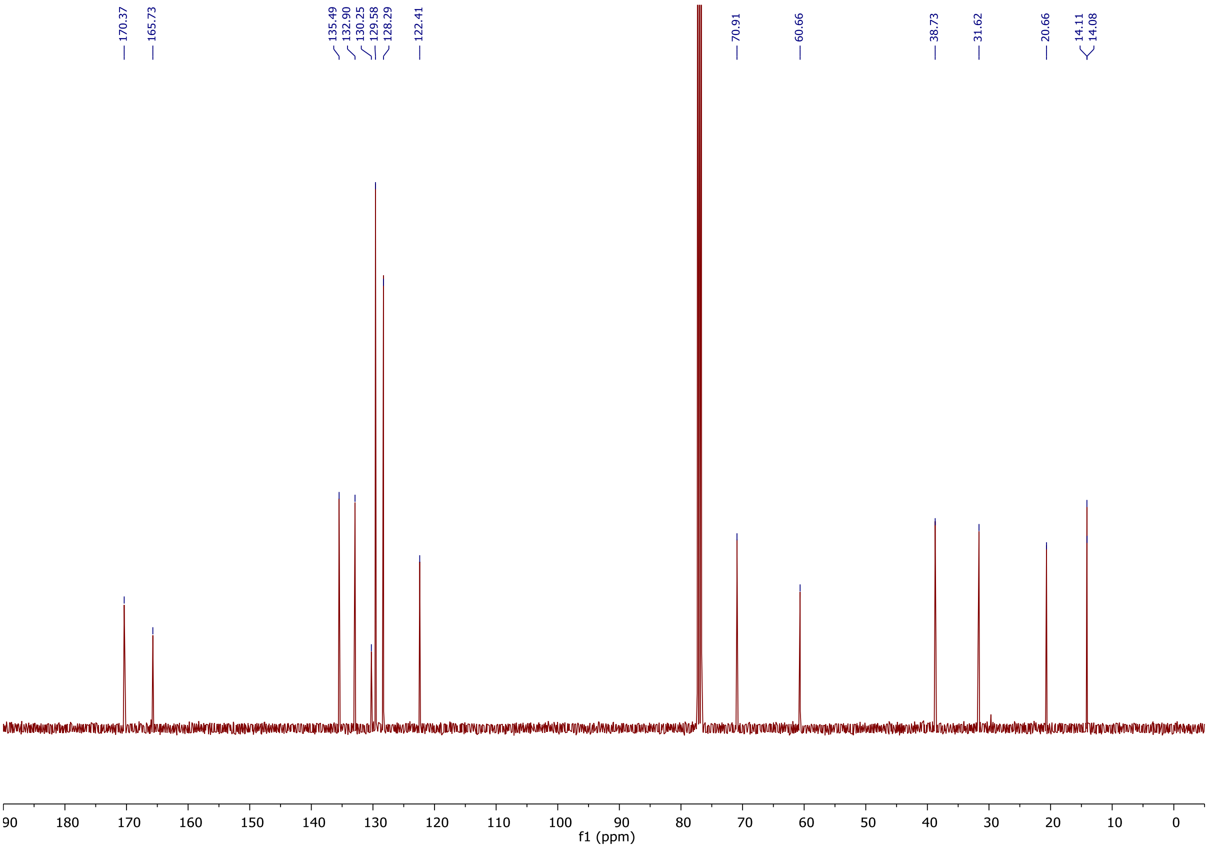
170.37
165.73
135.49 132.90 130.25 129.58 128.29
122.41
70.91
60.66
38.73
31.62
20.66
14.11 14.08
f1 (ppm)

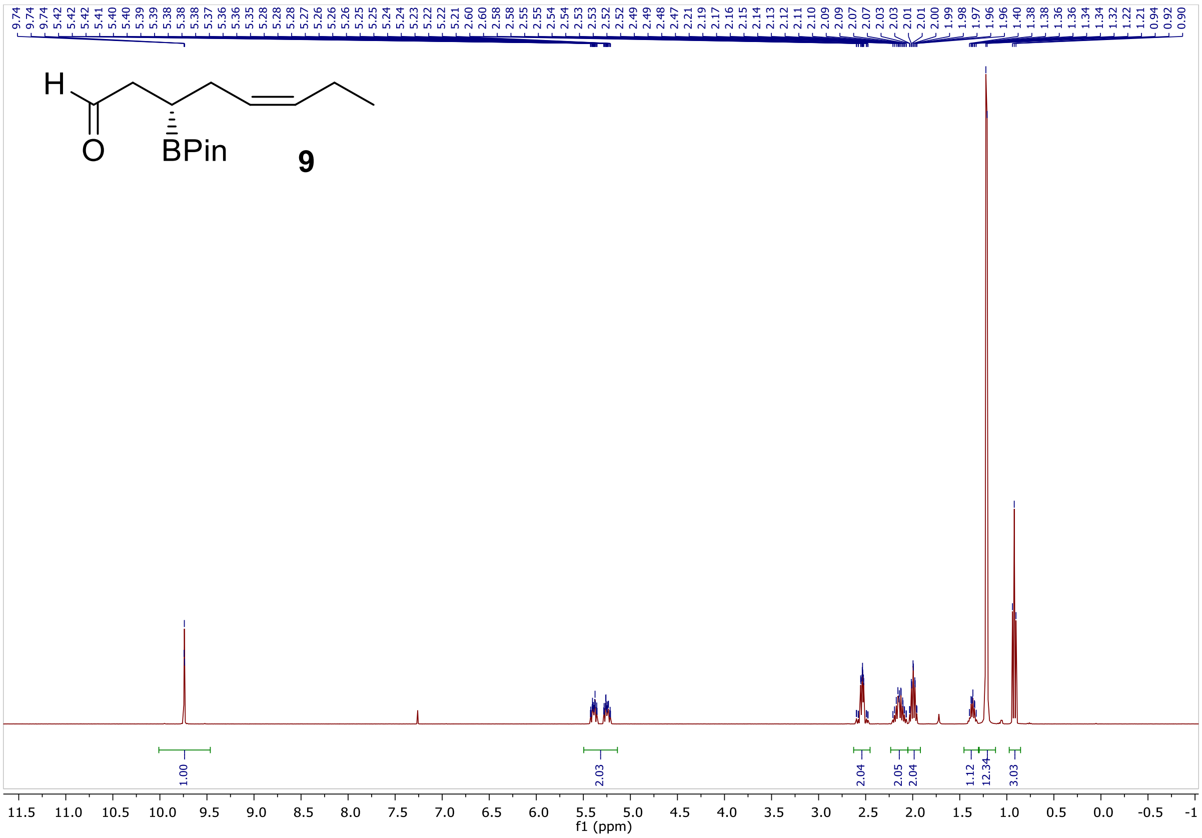
9.74
9.74
9.74
9.74
5.42
5.42
5.41
5.40
5.40
5.39
5.39
5.38
5.38
5.38
5.37
5.36
5.36
5.35
5.28
5.28
5.28
5.27
5.26
5.26
5.26
5.25
5.25
5.24
5.24
5.23
5.22
5.22
5.21
2.60
2.60
2.58
2.58
2.55
2.55
2.54
2.54
2.53
2.53
2.52
2.52
2.49
2.48
2.47
2.21
2.19
2.17
2.16
2.15
2.14
2.13
2.12
2.11
2.10
2.09
2.09
2.07
2.07
2.03
2.03
2.01
2.01
2.00
1.99
1.98
1.97
1.96
1.96
1.40
1.38
1.38
1.36
1.36
1.34
1.34
1.32
1.22
1.21
0.94
0.92
0.90
H
O
BPin
9
1.00
2.03
2.04
2.05
2.04
1.12
12.34
3.03
f1 (ppm)

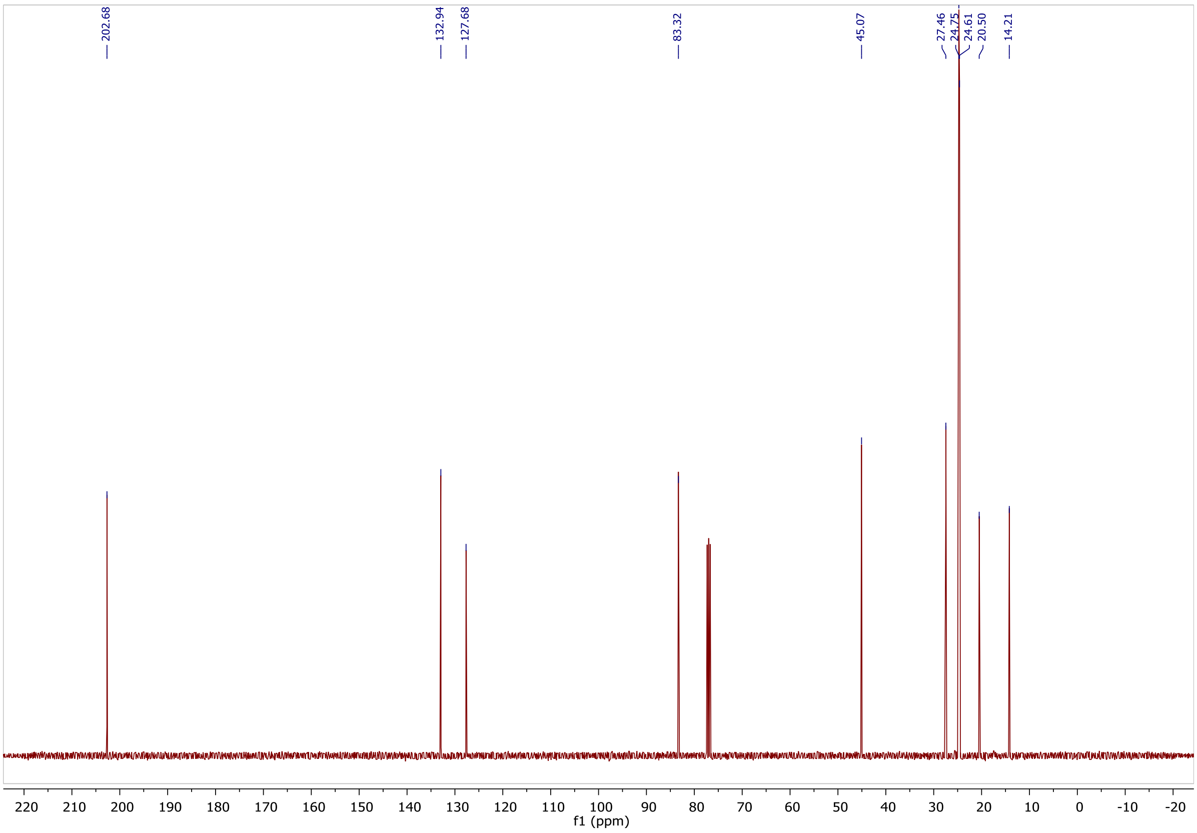
202.68
132.94
127.68
83.32
45.07
27.46
24.75
24.61
20.50
14.21
f1 (ppm)

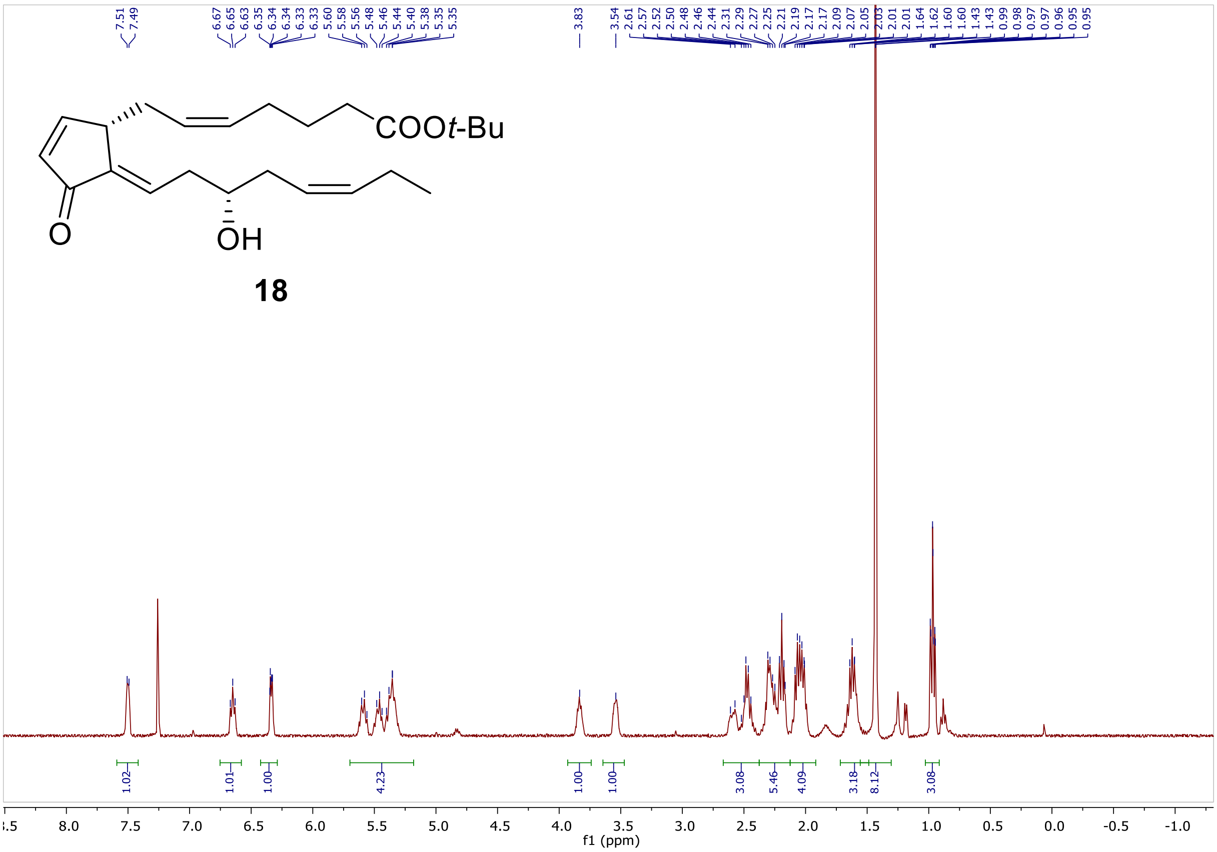
COOt-Bu
O
OH
18
f1 (ppm)

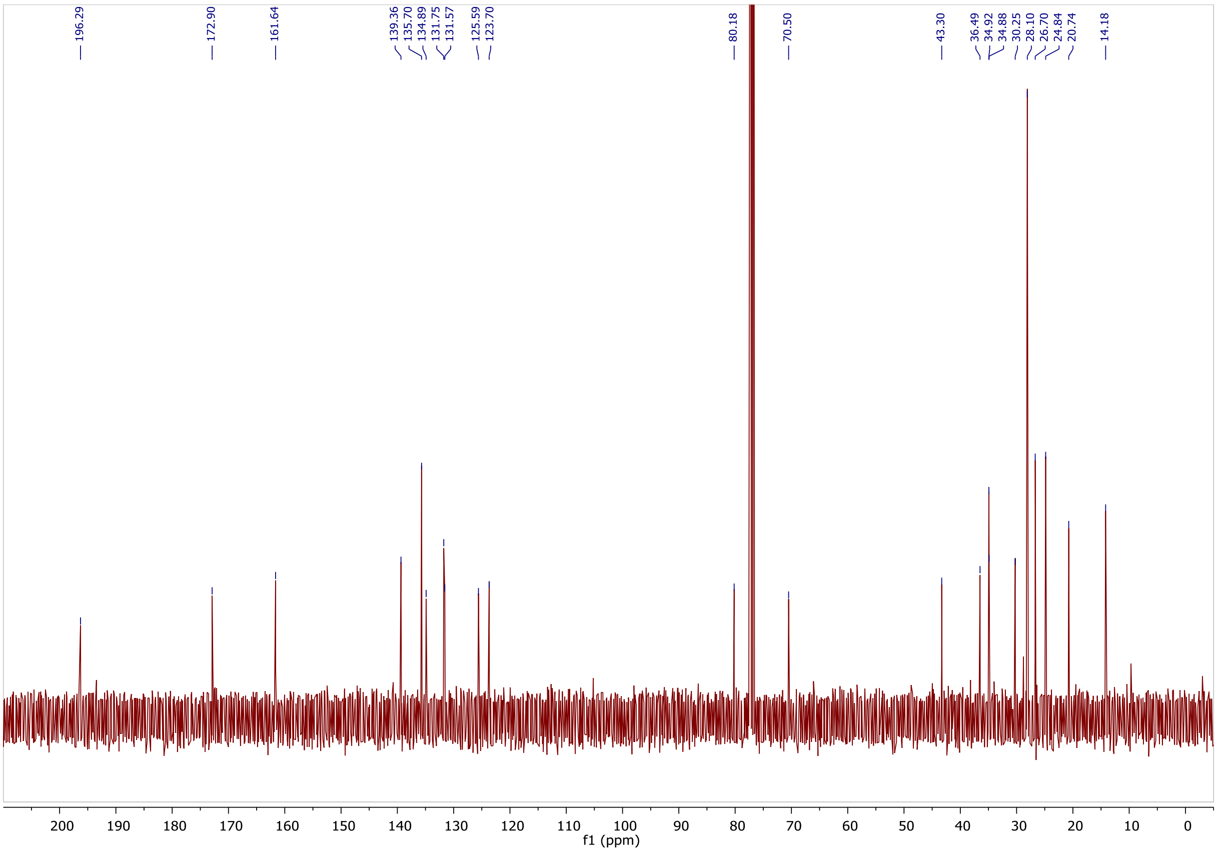
f1 (ppm)

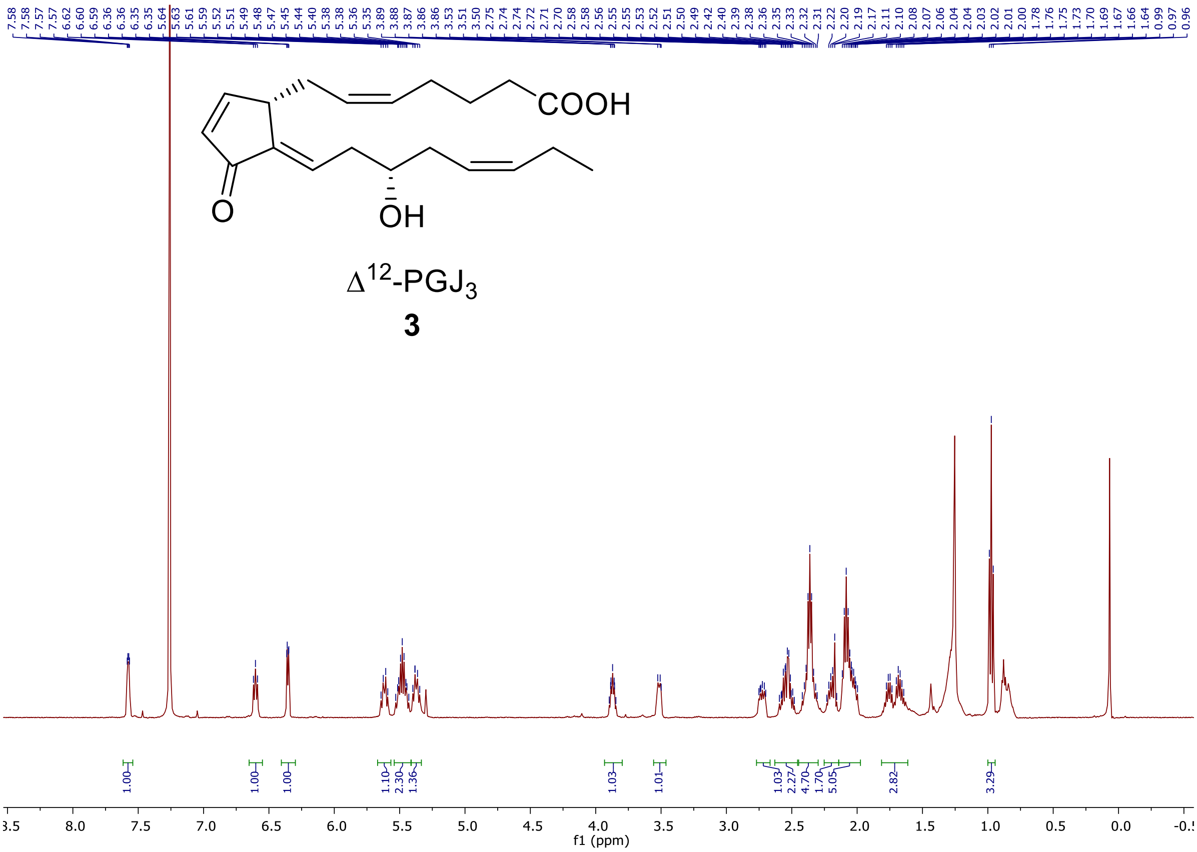

Δ^{12}-PGJ$_3$

3

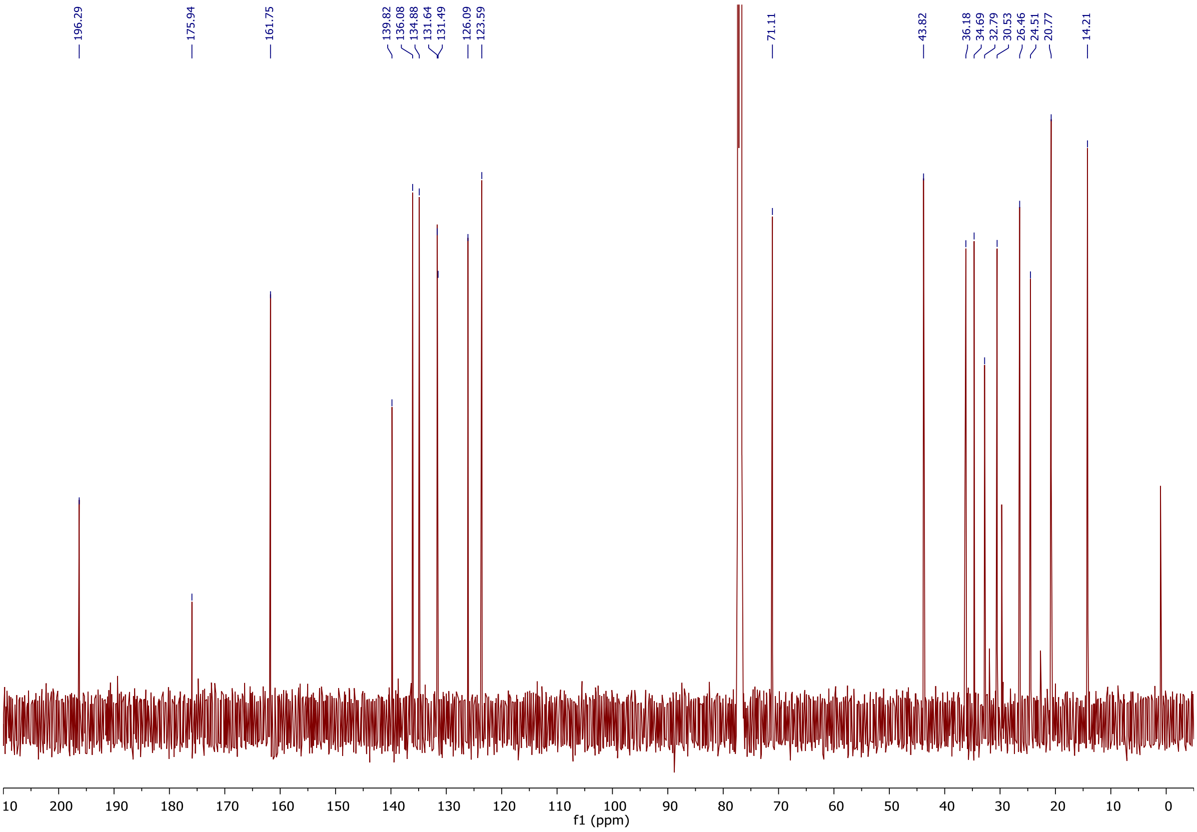

COSY spectrum (CDCl3, 500 MHz/500 MHz) of Δ[12]-PGJ3

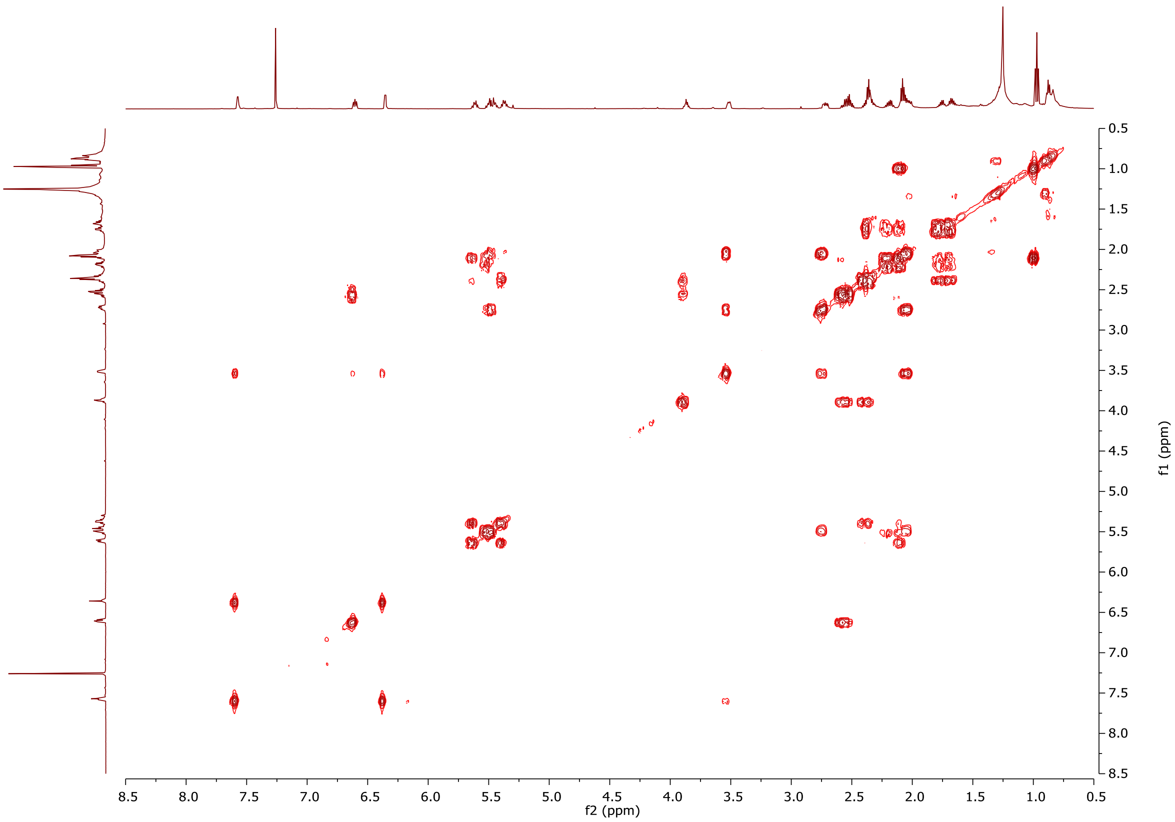

HSQC spectrum (CDCl3, 500 MHz/125 MHz) of Δ[12]-PGJ3

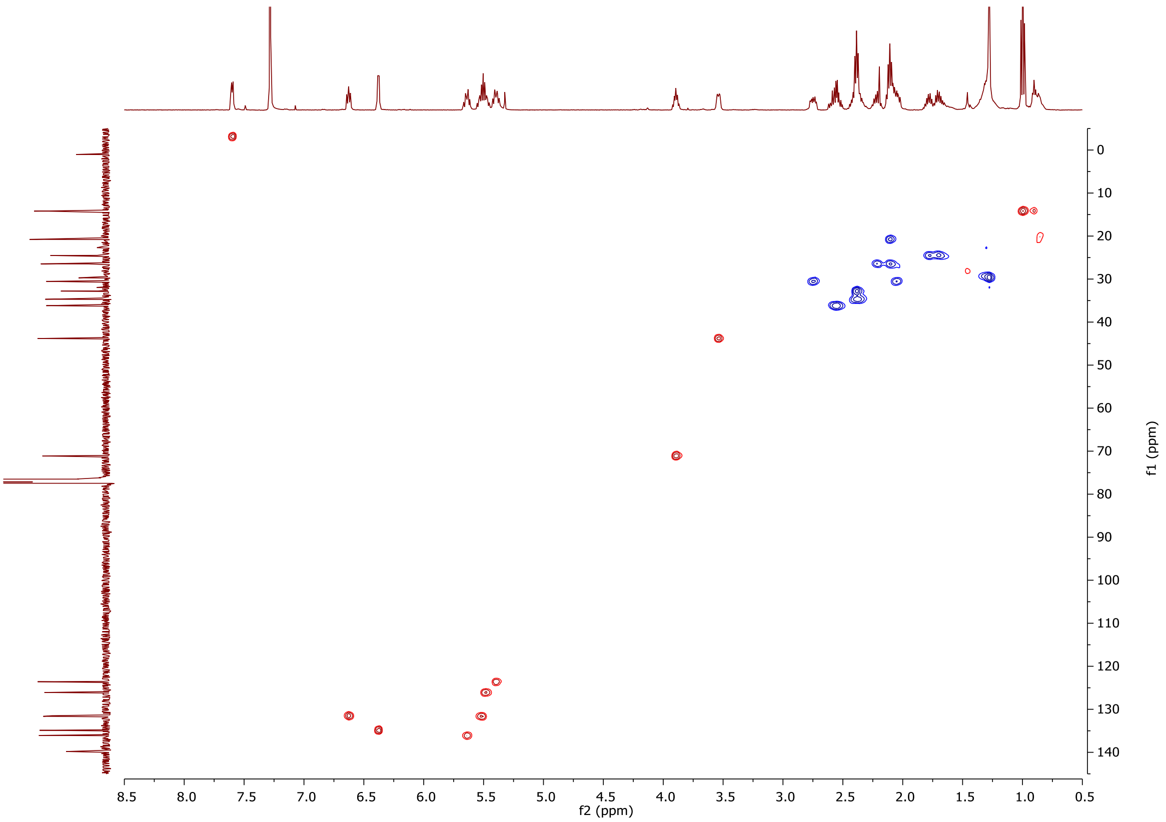

6. References

[1] K. Zumbansen, A. Döhring, B. List, *Adv. Synth. Catal.* **2010**, *352*, 1135-1138.

[2] G. Coulthard, W. Erb, V. K. Aggarwal, *Nature* **2012**, *489*, 278-281.

[3] S. Prevost, K. Thai, N. Schützenmeister, G. Coulthard, W. Erb, V. K. Aggarwal, *Org. Lett.* **2015**, *17*, 504-507.

[4] J. M. Hoover, S. S. Stahl, *J. Am. Chem. Soc.* **2011**, *133*, 16901-16910.

[5] K. C. Nicolaou, K. K. Pulukuri, R. Yu, S. Rigol, P. Heretsch, C. I. Grove, C. R. H. Hale, A. ElMarrouni, *Chem. Eur. J.* **2016**, *22*, 8559-8570.

[6] T. Honda, M. Ohta, H. Mizutani, *J. Chem. Soc., Perkin Trans. 1* **1999**, 23-30.
